# Nitrogen-Containing Flavonoids—Preparation and Biological Activity

**DOI:** 10.1021/acsomega.4c04627

**Published:** 2024-07-29

**Authors:** Martina Hurtová, Daniela Brdová, Bára Křížkovská, Guglielmo Tedeschi, Tomáš Nejedlý, Ondřej Strnad, Simona Dobiasová, Zuzana Osifová, Gabriela Kroneislová, Jan Lipov, Kateřina Valentová, Jitka Viktorová, Vladimír Křen

**Affiliations:** † Institute of Microbiology of the Czech Academy of Sciences, Vídeňská 1083, Prague 142 00, Czech Republic; ‡ Department of Biochemistry and Microbiology, University of Chemistry and Technology Prague, Technická 5, Prague 166 28, Czech Republic; § Institute of Organic Chemistry and Biochemistry of the Czech Academy of Sciences, Flemingovo nám. 542, Prague 160 00, Czech Republic; ∥ Department of Clinical Microbiology and ATB Center, Institute of Medical Biochemistry and Laboratory Diagnostics of the General University Hospital and of The First Faculty of Medicine of Charles University, U Nemocnice 2, Prague 2 128 08, Czech Republic

## Abstract

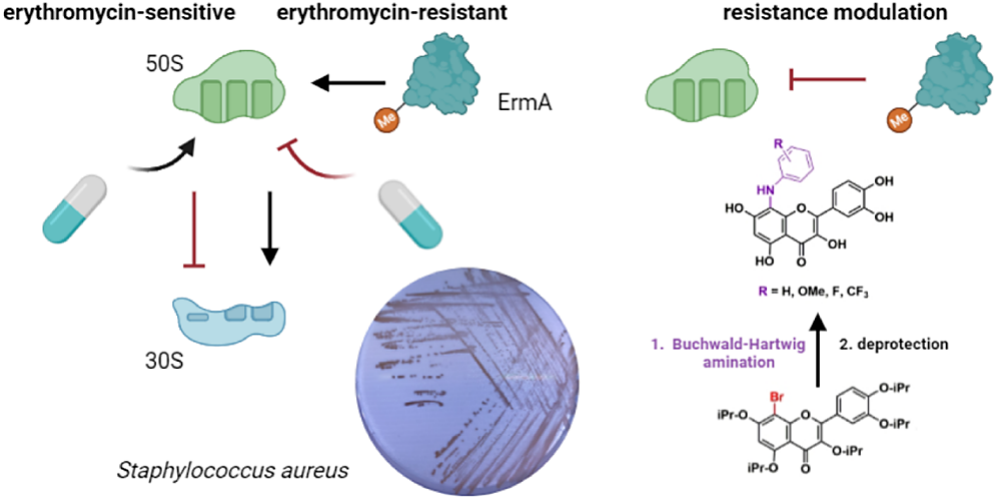

In this work, we report the application of Buchwald–Hartwig amination for the preparation of new derivatives of quercetin and luteolin. Our investigation delves into the impact of aniline moiety on antioxidant, and anti-inflammatory activity, cytotoxicity, and the ability of flavonoids to modulate drug-resistance mechanisms in bacteria. The anti-inflammatory activity disappeared after the introduction of aniline into the flavonoids and the cytotoxicity remained low. Although the ability of quercetin and luteolin to modulate bacterial resistance to antibiotics has already been published, this is the first report on the molecular mechanism of this process. Both flavonoids attenuate erythromycin resistance by suppressing the ribosomal methyltransferase encoded by the *ermA* gene in *Staphylococcus aureus*. Notably, 4-(trifluoromethyl)anilino quercetin emerged as a potent ErmA inhibitor, likely by interacting with the RNA-binding pocket of ErmA. Additionally, both 4-fluoroanilino derivatives effectively impended the staphylococcal efflux system. All the prepared derivatives exhibited superior activity in modulating gentamicin resistance in *S. aureus* compared to the parent compounds. Overall, the incorporation of substituted anilines into the flavonoid core significantly enhanced its ability to combat multidrug resistance in bacteria.

## Introduction

The introduction of new C–N bonds into aromatic compounds holds significant promise in medicinal chemistry and natural product synthesis.^1^ Aromatic amines are integral components found in various biologically active compounds, such as benzodiazepines and cyclin-dependent kinase inhibitors.^2^ However, methods for introducing amino groups into aromatic compounds remain limited. The Ullmann reaction was described in 1903 as a copper-catalyzed coupling of aromatic halides with amines,^3^ followed by a similar Goldberg reaction used for the arylation of amides.^4^ These reactions have limitations, including high reaction temperatures, a narrow scope, and the need for equimolar amounts of copper. Over the years, reaction conditions for Ullmann-type reactions have been optimized to proceed at lower temperatures, and Cu^0^ has been replaced by Cu(I), often in combination with various bidentate N, N- or N, O- ligands such as oxalic acid diamides or amino acids.^5,6^

In 1994, Buchwald^7^ and Hartwig^8^ simultaneously published studies on the Pd-catalyzed cross-coupling reaction of aromatic halides with amines. This reaction has since gained widespread recognition for its versatility, low catalyst loading, and cost-effectiveness, making it a cornerstone method for C–N bond formation in both academia and the pharmaceutical industry over the past three decades.^9^

Flavonoids have garnered significant attention as naturally occurring compounds due to their diverse biological activities and generally low toxicity.^10^ Previously reported synthetic and naturally occurring nitrogen-containing flavonoids exhibited interesting biological activities. For example, azaquercetin and azaluteolin have been reported to act as influenza endonuclease inhibitors.^11^ Rohitukine, a naturally occurring flavonoid substituted at C-8 with *N*-methyl-3-hydroxy-piperidine, showed anticancer, anti-inflammatory, and immunomodulatory activities.^12^ Rohitukine and its synthetic derivatives flavopiridol and riviciclib are currently in various stages of clinical trials as potential anticancer drugs that act as CDK inhibitors and apoptosis inducers.^13^

Furthermore, as recently summarized by Nguyen and Bhattacharya,^14^ quercetin and its derivatives can modulate antibiotic resistance and act synergistically with antibiotics. Since the search for new antimicrobial agents seems to be insufficient in recent decades, the possibility of inhibiting resistance mechanisms and restoring the efficacy of old antibiotics presents a promising approach in the fight against resistant pathogens.^15^ Quercetin restored the efficacy of fluconazole in fluconazole-resistant *Candida tropicalis*,^16^ meropenem in carbapenem-resistant *Escherichia coli* and *Klebsiella pneumoniae*,^17^ amoxicillin in amoxicillin-resistant *Staphylococcus epidermidis*,^18^ levofloxacin, ceftriaxone, gentamicin, tobramycin and amikacin in multidrug-resistant *Pseudomonas aeruginosa*,^19^ florfenicol in *Aeromonas hydrophila*,^20^ colistin and of amikacin in colistin-resistant *Acinetobacter baumannii*.^21^ Quercetin conjugate with pivaloxymethyl restored the activity of ampicillin, cefepime, and vancomycin in multidrug-resistant strains of *Staphylococcus aureus* and *Enterococcus faceium*.^22^ Moreover, both quercetin and luteolin inhibited β-lactamase and acted synergistically with ceftazidime in β-lactamase-producing *Streptococcus pyogenes*,^23^ the three-component mixture of flavonoids (quercetin, morin, rutin) acted synergistically with amoxicillin, ampicillin, cephradine, ceftriaxone, imipenem, and methicillin in methicillin-resistant *S. aureus*(24) and quercetin alone had an additive effect with ampicillin, cephradine, ceftriaxone, imipenem, and methicillin in methicillin-resistant *S. aureus*.^24^ Quercetin also inhibited case in hydrolase P—the virulence factor in *S. aureus*(25) and targeted quorum sensing of *P. aeruginosa*.^19^

The compounds in this study were designed based on our previous findings, which focused on the preparation of halogenated derivatives of flavonoids and the effects of this substitution on biological activity. We have reported that halogenation enhances the synergistic effect of flavonoids with antibiotics,^26^ but only fluorinated flavonoids were absent in this work due to the complicated preparation. Halogenated derivatives were then used as reactive intermediates for Suzuki cross-coupling reactions–allowing the fluorine to be introduced into the flavonoid core via reaction with fluoroboronic acids.^27^ Unfortunately, according to our unpublished results, the products of Suzuki cross-coupling reactions did not show improved biological effects. Therefore, we hypothesized that the introduction of the amino group may lead to such an improvement. Halogenated derivatives can be used as starting material for Buchwald–Hartwig amination or Ullmann-type reactions, which allow the preparation of synthetic, nitrogen-containing, fluorine-substituted flavonoids.

Buchwald–Hartwig amination has so far only been described for 6-bromoflavone, which lacks the −OH groups. Fitzmaurice et al. reported a cross-coupling of 6-bromoflavone and *n*-hexylamine affording 6-hexylamine flavone.^28^ 6-Bromoflavone also reacted with amino acids to form flavone-amino acid hybrids.^29^

The aim of this study was to prepare a library of novel aniline-substituted flavonoids with various substituents, including fluorine, which have the potential to improve the biological activity of flavonoids. The derivatives prepared were subjected to extensive testing, including antioxidant and anti-inflammatory activity, cytotoxicity to human fibroblasts and keratinocytes, and the ability to modulate multidrug resistance in bacteria.

## Results and Discussion

### Chemistry

#### Optimization of Buchwald–Hartwig Amination

A series of alkyl and aryl amines was tested for the coupling, namely: *n*-hexylamine, 4-fluoroaniline, 4-methoxyaniline (*p*-anisidine), aniline, 4-(trifluoromethyl)aniline, 3,5-dimethoxyaniline, 4-fluoroaniline, 4-(trifluoromethoxy)aniline, morpholine, and piperidine. The reaction of 8-iodo-3,3′,4′,5,7-penta-*O*-isopropoxy quercetin with *n*-hexylamine under the previously published conditions (Pd_2_(dba)_3_, BINAP, NaOtBu, toluene)^28^ failed. 8-Bromo-3,3′,4′,5,7-penta-*O*-isopropoxy quercetin prepared using our previously published method^26^ was used under the same reaction conditions with *n*-hexylamine, but this reaction failed as well. The reaction was later optimized with more reactive arylamines such as *p*-anisidine and 4-fluoroaniline (Table 1). Initially, the reaction was conducted with *p*-anisidine using BINAP and various bases in toluene at 100 °C (Entries 1–6, Table 1). Under these reaction conditions, only traces of the product were detected in the HPLC/MS analysis. To improve the reaction efficiency, BINAP was replaced by more sterically hindered ligands such as tBuXPhos or SPhos. Additionally, the use of NaO*t*Bu as the base led to an improved HPLC conversion of 30%. Interestingly, optimization efforts revealed that lower reaction temperatures favored higher conversion rates. Further experimentation without microwave irradiation (Entry 15, Table 1) yielded similar outcomes, albeit requiring a longer reaction time of 16 h.

**Table 1:** Conditions Used for the Optimization of the Reaction Conditions^a^

entry	amine	ligand	base	solvent	T [°C]	time [h]	yield [%]
1	*p*-anisidine	BINAP	Cs_2_CO_3_	toluene	110	16	0
2	*p*-anisidine	BINAP	NaO*t*Bu	toluene	110	2	0
3	*p*-anisidine	BINAP	K_3_PO_4_	toluene	110	16	0
4	*n*-hexylamine	BINAP	K_3_PO_4_	toluene	110	16	0
5	*p*-anisidine	BINAP	HMDS	toluene	100	16	decomposition
6	*p*-anisidine	BINAP^b^	K_2_CO_3_	toluene	80	16	0
7	*p*-anisidine	*t*BuXPhos	K_2_CO_3_	toluene	100	16	0
8	*p*-anisidine	*t*BuXPhos	NaO*t*Bu	toluene	140	16	20
9	*p*-anisidine	*t*BuXPhos	LiHMDS	toluene	140	16	decomposition
10	*p*-anisidine	*t*BuXPhos	NaO*t*Bu	toluene	120	48	30%^c^
11	4-fluoroaniline	*t*BuXPhos	NaO*t*Bu	toluene	120	48	40%^c^
12	4-fluoroaniline	SPhos	NaO*t*Bu	toluene	120	16	40%^c^
13	4-fluoroaniline	*t*BuXPhos	NaO*t*Bu	1,4-dioxane	85	2^d^	40
14	4-fluoroaniline	*t*BuXPhos	NaO*t*Bu	toluene	80	2^d^	40
15	4-fluoroaniline	*t*BuXPhos	NaO*t*Bu	THF	70	16	50
16	4-fluoroaniline	*t*BuXPhos	NaO*t*Bu	THF	70	2^d^	54

a Pd_2_(dba)_3_ was used as a catalyst unless otherwise stated.

b Pd(OAc)_2_ was used as a catalyst.

c Conversion was determined by HPLC analysis.

d The reaction was carried out in a microwave reactor.

**Figure 1:**
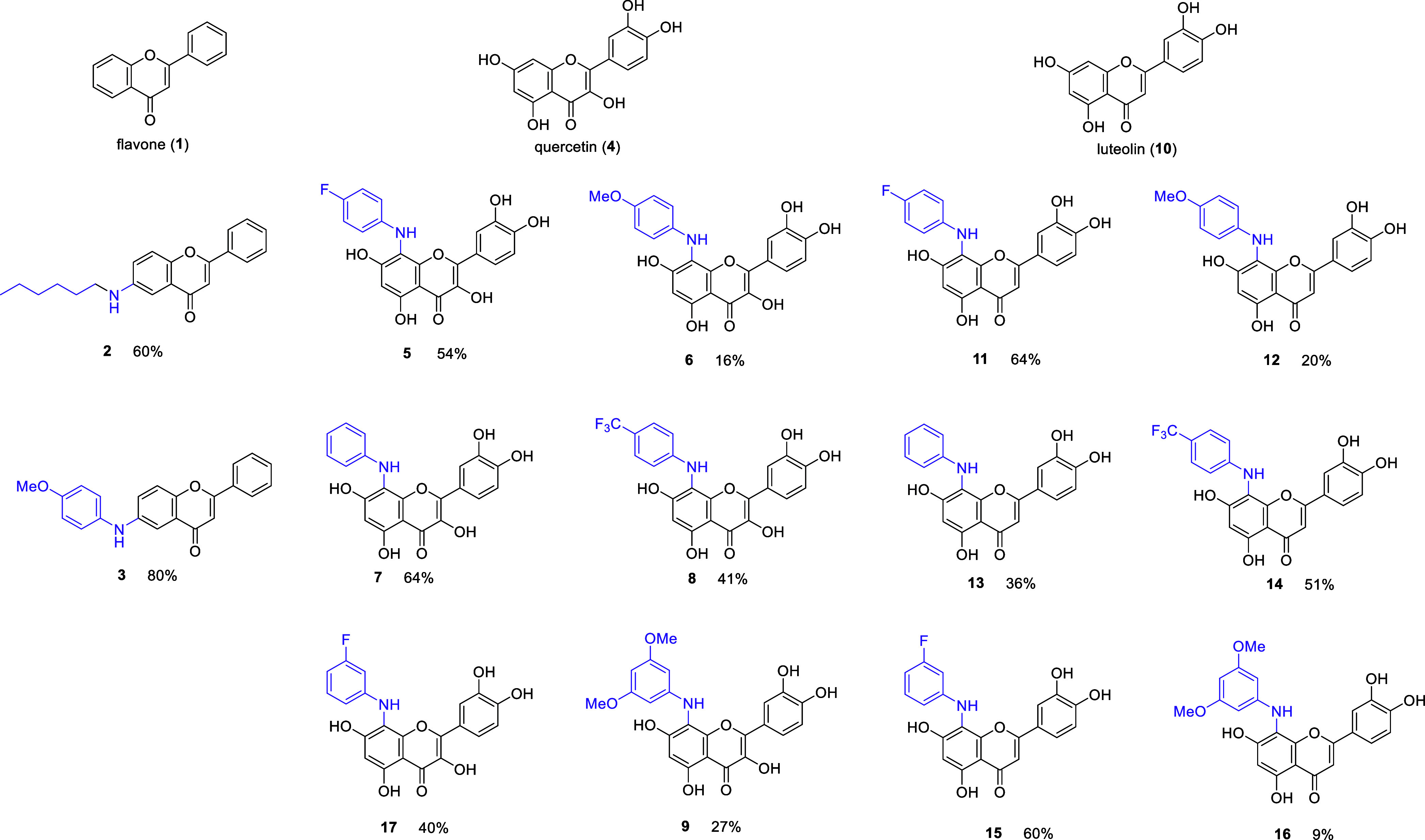
Structures of prepared derivatives and isolated yields.

After successful optimization of the reaction conditions (Entry 16, Table 1), a library of flavone, quercetin, and luteolin derivatives substituted at C-8 with various anilines was prepared using the Buchwald-Hartwig amination. The prepared derivatives and the isolated yields are summarized in Figure 1.

The reaction with 4-(trifluoromethoxy)aniline proved unsuccessful, likely attributed to the strong electron-withdrawing effect of the −OCF_3_ group, which might have hindered the desired reactivity. Similarly, attempts to react with alkylamines such as *n*-hexylamine, morpholine, and piperidine under optimized reaction conditions also failed. This could be attributed to the relatively low nucleophilicity of alkylamines compounded by steric hindrance at C-8 of the flavonoid. In general, the presence of an alkyl group (e.g., prenyl, allyl) in the flavonoid backbone typically enhances biological activity and lipophilicity.^30^ The reaction with *n*-hexylamine was successful only with flavone which lacks OH groups on the A-ring, affording compound **2**.

#### Optimization of Ullmann Reaction

Given the unsuccessful outcomes of the Buchwald-Hartwig reaction, an alternative approach using the Ullmann-type reaction for the introduction of alkylamines was investigated. Previously reported reaction conditions (1 equiv amine, 2.5 equiv K_2_CO_3_, 10% CuI, DMF, 0.1 mL water)^5^ were used for the reaction of 8-bromo flavonoids with *n*-hexylamine, and this reaction was as unsuccessful as a reaction with glycine methyl ester. Other attempts employed a CuI/amino acid-promoted reaction of aryl halogens with amines catalyzed with CuI and amino acids such as l-proline or *N*,*N*-dimethylglycine.^6^ The coupling of 8-bromo-3,3′,4′,5,7-penta-*O*-isopropoxy quercetin with *n*-hexylamine, or morpholine carried out in the presence of CuI, proline, and K_2_CO_3_, did not yield the desired products. All unsuccessful attempts to optimize the Ullmann reaction are summarized in Table S1. Unfortunately, attempts to produce flavonoids substituted at C-8 with alkylamines failed, probably due to activation of the A-ring of the flavonoids and steric hindrance at the C-8 position.

### Biological Activity

All quercetin and luteolin derivatives prepared were screened for biological activity. Cytotoxicity, antioxidant, antibacterial, and anti-inflammatory activities as well as the ability of the prepared derivatives to affect the multidrug resistance of bacteria were investigated and compared with the parent flavonoids.

#### Antioxidant and Anti-Inflammatory Activity

To evaluate the antioxidant activity of the prepared derivatives and the respective parent compounds, cellular antioxidant activity (CAA) was measured in macrophages. Compared to other *in vitro* antioxidant assays, this method provides partial information about the uptake and metabolism of the compounds, because, in this assay, they must first enter the cell to exert antioxidant activity.^31^ The parent compounds quercetin and luteolin showed antioxidant activity with IC_50_ values of 5.85 μM and 4.48 μM, respectively (Table S2). Derivatization of quercetin at C-8 with 4-fluoroaniline (**5**) increased the antioxidant activity of quercetin by 1.7-fold. Among luteolin derivatives, the 8-(4-fluoroanilino) luteolin (**11**) and 8-anilino luteolin (**13**) showed better antioxidant activity than the parent compound. The concentration of these derivatives required to halve the cellular radicals decreased 1.5-fold compared to luteolin. On the other hand, derivatization with 4-(trifluoromethyl)aniline (**8**, **14**) and the 3-fluoroaniline moiety (**15**) significantly decreased the antioxidant activity.

The anti-inflammatory activity of flavonoids was evaluated as their ability to decrease the production of signaling molecules that mediate inflammation, such as nitric oxide (NO), tumor necrosis factor (TNF-α), and interleukin 6 (IL-6), in LPS-stimulated macrophages. Parent compounds showed significant activity in these assays, as reported previously,^32,33^ but the activity of amino derivatives decreased in all assays (Table S3), except for 8-(4-fluoroanilino) luteolin (**11**), 8-anilino luteolin (**13**), and 8-(3-fluoroanilino) luteolin (**15**) which exhibited the same ability to inhibit IL-6 production as luteolin.

#### Cytotoxicity of Flavonoids on Healthy Cells

The cytotoxicity of the prepared derivatives was evaluated by their ability to decrease the viability of immortalized human keratinocytes (HaCaT) and human dermal fibroblasts (HDF). The concentrations of flavonoids that halved the viability of the respective cells are summarized in Table S4. The synthetic modification of the flavonoid scaffold only increased the very low toxicity of quercetin in HaCaT. The IC_50_ values were above 25 μM in all cases, compared to less than 1 μM for doxorubicin.

#### Modulation of Antibiotic-Resistant Phenotype in Bacteria

To evaluate the ability to modulate bacterial resistance, the tested flavonoids themselves cannot exert antimicrobial activity; therefore, the nontoxic concentration was used, as indicated in Table S5. Clinically relevant antibiotics were selected at breakpoint concentrations for susceptibility testing according to the European Committee on Antimicrobial Susceptibility Testing (EUCAST, version 11.0). Erythromycin and gentamicin at breakpoint concentrations (1 mg/L) did not affect the multidrug-resistant clinical strain of *S. aureus* (MRSA). Flavonoids and their derivatives (from 1 μM to the highest nontoxic concentration) were used in combination with the breakpoint concentration of the antibiotics. After coincubation, the minimum inhibitory concentration (MIC) of flavonoids inhibiting the visible growth of bacteria was determined (Table 2).

**Table 2:** Minimum Inhibitory Concentration of Quercetin, Luteolin, and their Derivatives Inhibiting the Visible Growth of *Staphylococcus aureus* MRSA 9 in the Presence of Breakpoint Concentration of Antibiotic (1 mg/L)^a^^b^^c^

	gentamicin		erythromycin	
quercetin (**4**)	>200 ^note 1^		117.3 ± 4.7	
8-(4-fluoroanilino) quercetin (**5**)	45.2 ± 0.6	***	>200	
8-(4-methoxyanilino) quercetin (**6**)	>200 ^note 2^		>200	
8-(anilino) quercetin (**7**)	43.8 ± 1.6	***	>200	
8-(4-(trifluoromethyl)anilino) quercetin (**8**)	8.1 ± 0.6	***	>25 ^note 3^	
8-(3,5-dimethoxyanilino) quercetin (**9**)	>200		>200	
luteolin (**10**)	84.7 ± 1.8		147.3 ± 7.7	
8-(4-fluoroanilino) luteolin (**11**)	7.9 ± 0.5	***	>25 ^note 4^	
8-(4-methoxyanilino) luteolin (**12**)	44.0 ± 1.6	***	76.3 ± 1.9	***
8-(anilino) luteolin (**13**)	24.7 ± 0.8	***	>200	
8-(4-(trifluoromethyl)anilino) luteolin (**14**)	7.6 ± 0.1	***	>15 ^note 5^	
8-(3-fluoroanilino) luteolin (**15**)	12.4 ± 0.3		>200	

a Data are presented as the minimum inhibitory concentration (MIC, μM); average of three repetitions ± standard error of the mean.

b Stars indicate the statistically improved activity when compared to the parent compound (Students t-test, *p < 0.05, ** *p* < 0.005, ***p < 0.0005).

c The notes indicate that the maximum concentration used inhibited bacterial growth to: ^1^57.0 ± 1.2, ^2^50.7 ± 0.3, ^3^57.0 ± 0.6%, ^4^36.3 ± 1.8%, and ^5^37.7 ± 1.3%.

The prepared derivatives showed the ability to revert the gentamicin-resistant phenotype into a sensitive one in MRSA. 8-(4-Trifluoromethyl anilino) quercetin (**8**) reverted the resistant phenotype to a sensitive one at 8.1 μM, as did 8-(4-fluoroanilino) luteolin (**11**) with a MIC of 7.9 μM. In addition, both derivatives of quercetin and luteolin substituted at C-8 by aniline reversed the resistant phenotype with MIC values of 43.83 μM, and 24.66 μM, respectively. The derivative 8-(4-trifluoromethyl anilino) luteolin (**14**) reversed the resistant phenotype at 7.63 μM. On the other hand, 8-(4-methoxyanilino) luteolin (**12**) was the only derivative that was able to revert erythromycin-resistant MRSA into a sensitive one with a MIC of 76.3 μM. This luteolin derivative was more than twice as active as the parent compound.

To our knowledge, i) this is the first study demonstrating the synergistic effect of quercetin or luteolin with erythromycin, ii) a number of the derivatives prepared were more potent than the parent compounds, and iii) the therapeutic indexes have increased significantly considering that the compounds are used at nontoxic concentrations. Future research should aim to contextualize our results within the existing knowledge to better understand the novelty and implications of our findings.

#### Modulation of Bacterial Efflux System

**Figure 2:**
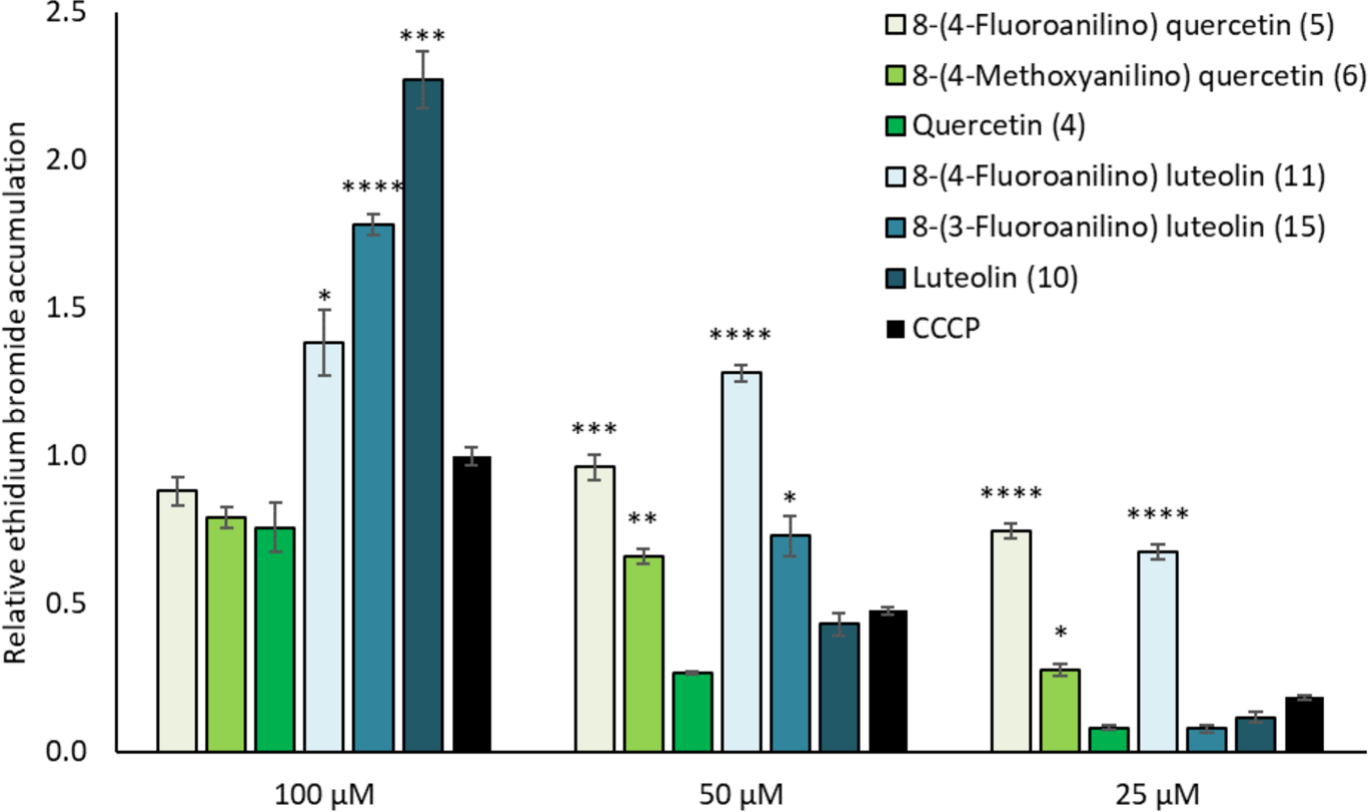
Relative accumulation of ethidium bromide after inhibition of transmembrane efflux pumps by CCCP (comparator, black), quercetin and its derivatives (green), and luteolin and its derivatives (blue). Data are presented as averages of three repetitions with corresponding standard error of the mean. Data are presented as relative to CCCP (100 μM). Statistical significances were performed using a *t*-test comparing compounds with CCCP in corresponding concentration points (*p < 0.05, ** *p* < 0.005, ***p < 0.0005, ****p < 0.00005).

The application of antibiotics often leads to oxidative stress in bacteria, which triggers the efflux pump systems that contribute to antimicrobial resistance.^34^ Therefore, modulation of the efflux system has been proposed as another plausible target of derivatives. The efflux pump inhibitor carbonyl cyanide 3-chlorophenylhydrazone (CCCP) was used as a comparator in our assay. Its action destroys the proton-motive force at the membrane so that the efflux pump cannot transport substrates out of the cell. One such universal substrate is ethidium bromide, the accumulation of which in the cell can be measured using fluorescence. As can be seen in Figure 2, the administration of 25–100 μM CCCP increases the accumulation of ethidium bromide in the cell. Compared to CCCP, both luteolin and quercetin are also efflux inhibitors, but only luteolin was significantly better than CCCP at the same concentration (100 μM). In contrast, the quercetin derivatives were significantly better inhibitors than the comparator CCCP at the lower concentrations tested (25 and 50 μM). At a concentration of 25 μM, 8-(4-fluoroanilino) quercetin (**5**) showed a 75% accumulation of ethidium bromide compared to 100 μM CCCP. Luteolin derivatives and, in particular, 8-(4-fluoroanilino) luteolin (**11**) also showed higher activity than CCCP alone. At a concentration of 25 μM, the activity was comparable to 8-(4-fluoroanilino) quercetin (**5**).

#### Mechanism of Erythromycin-Resistance Modulation in *S. aureus*

Erythromycin is a macrolide antibiotic that inhibits proteosynthesis by binding to the 50S subunit of the ribosome. In *Staphylococcus* species, resistance to erythromycin is associated with the presence of methyltransferases (encoded by *erm* genes) that alter the ribosomal target site. Another typical mechanism of resistance to erythromycin is the presence of an ATP-dependent efflux pump encoded by the *msr(A)* gene or a major facilitator superfamily (MFS) pump encoded by the lmrS gene. Both efflux pumps reduce the concentration of erythromycin in the cell below the therapeutic dose by transporting it across the membrane to the extracellular space. Another erythromycin resistance gene is the *mph(C)* gene, the product of which is a phosphotransferase that alters the structure of macrolide antibiotics. To elucidate the mechanism of erythromycin resistance modulation in *S. aureus*, further tests were performed with the strains MRSA 331 and MRSA 3596, which inactivate erythromycin via a single mechanism (*ermC* and *ermA*, respectively). At the same time, all strains possess the transmembrane efflux pump LmrS. Therefore, if the activity was not strain-specific, it would be necessary to consider the effect of the derivatives on this pump (Table 3).

**Table 3:** Characterization of the Mechanism of Resistance to Erythromycin in Clinical Isolates of *S. aureus*

a gene of erythromycin resistance	MRSA 9	MRSA 331	MRSA 3596
*ermA*	positive	negative	positive
*ermB*	positive	negative	negative
*ermC*	positive	positive	negative
*msrA*	negative	negative	negative
*mphC*	negative	negative	negative
*lmrS*	positive	positive	positive

The mechanism was determined based on the presence/absence of the gene responsible for antibiotic modification in both plasmid and genomic DNA using PCR.

Both quercetin and luteolin showed synergistic activity with erythromycin against the *S. aureus* strain MRSA 3596 (Table 4), which uses the *ermA* gene product to eliminate the antibiotic. The quercetin derivative 8-(4-(trifluoromethyl)anilino) quercetin (**8**) was even 7 times more effective than its parent compound. Due to its lower toxicity, its therapeutic index is equal to 13.94 and 13.32 for the tested HaCaT and HDF cell lines, respectively, which makes it a drug with a suitable therapeutic profile. On the contrary, none of the luteolin derivatives was more effective than the parent compound, only the 8-(4-methoxyanilino) luteolin (**12**) derivative retained the original properties of luteolin.

**Table 4:** Mode of Antimicrobial Action of Quercetin (**4**), Luteolin (**10**), and their Derivatives with Erythromycin Against Methicilin-Resistant *S. aureus* 331 Strain Positive for the Presence of the *ermC* Gene and 3596 Strain Positive for *ermA* Gene Encoding Ribosomal Methyltransferases^a^

	MRSA 331				MRSA 3596			
	MIC [μM]				MIC [μM]			
erythromycin [1 mg/L]	yes	no	FIC	effect	yes	no	FIC	effect
quercetin (**4**)	190.9 ± 0.5	193.1 ± 1.0	0.99 ± 0.01	additive	58.6 ± 1.2	178.1 ± 2.7	0.33 ± 0.01	synergy
8-(4-(trifluoromethyl)anilino) quercetin (**8**)	15.9 ± 0.8	6.9 ± 0.2	2.30 ± 0.03	antagonism	8.4 ± 1.1	37.9 ± 0.1	0.22 ± 0.03	synergy
luteolin (**10**)	31.6 ± 2.5	28.1 ± 0.3	1.12 ± 0.10	indifference	14.3 ± 0.9	51.1 ± 1.3	0.28 ± 0.02	synergy
8-(4-fluoroanilino) luteolin (**11**)	57.4 ± 0.2	57.6 ± 0.2	1.00 ± 0.01	indifference	28.0 ± 0.5	32.9 ± 0.6	0.85 ± 0.03	additive
8-(4-methoxyanilino) luteolin (**12**)	54.8 ± 0.4	54.4 ± 2.1	1.01 ± 0.05	indifference	20.0 ± 0.6	53.6 ± 0.5	0.37 ± 0.01	synergy
8-(4-(trifluoromethyl)anilino) luteolin (**14**)	27.0 ± 1.5	27.0 ± 5.1	1.00 ± 0.25	indifference	29.9 ± 1.4	14.4 ± 0.1	2.07 ± 0.11	antagonism

a Fractional Inhibitory Concentration Index (FIC) < 0.5 indicates synergism, > 0.5–1 indicates additive effects, > 1 to <2 indicates indifference, and ≥2 indicates antagonism.

#### Molecular Docking Analyses

Molecular docking experiments revealed that quercetin, luteolin, and their derivatives share a binding pocket with *S*-adenosyl-l-methionine (SAM), indicating possible competition for the binding site. On the other hand, blind experiments showed a clear change in the docking trend. All tested molecules showed positive docking in the RNA (central) pocket, with a larger volume available. These results have revealed intriguing insights into the role of these molecules.

The low alphafold score confidence for the N-terminal region does not allow a complete interpretation of the binding prediction due to the flexibility of this region. However, the influence of the 8TQNF11 motif could play an interesting role in the N-terminal motions preventing the accommodation of tested molecules within the SAM pocket.

Dynamics and mutations are variables that could strongly prevent the expected docking of the derivatives either in the upper pocket, a phenomenon observed in the ErmB parent possessing the mutation 8SQNF11, or in the RNA pocket. The mentioned mutation is of particular interest for future studies on the importance of this residue for enzyme activity. In addition, the docking positions of all molecules in the central part of the protein, shown in the blind docking, offer reasonable potential for influencing enzymatic activity. This region appears to be involved in the electrostatic interaction with the target RNA, as studied by Goh et al.^35^

As mentioned above, blind docking can exhibit highly variable behavior in binding modes. By constraining the algorithm’s search in the binding pocket, we were able to better interpret the binding modes in the putative binding pocket. The lack of experimental data on the studied system prompted us to validate our docking experiments on a closely related system: ermC (pdbid: 1QAN) studied by Schluckebier et al.^36^

**Figure 3:**
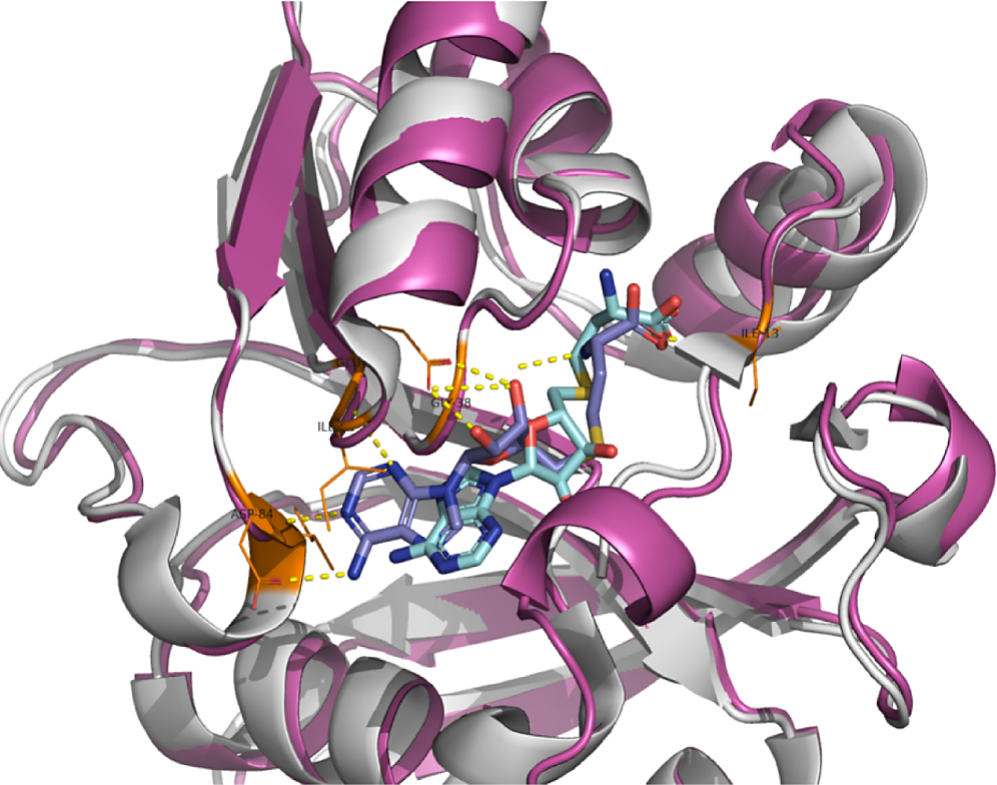
Docking of *S*-adenosyl-methionine into the model of methyltransferase. *S*-Adenosylmethionine (SAM) crystal structure (dark blue) within ermC crystal structure (gray) and docked SAM (light blue) within predicted ermA (violet).

*S*-Adenosylmethionine (SAM) crystal structure (dark blue) within ermC crystal structure (gray) and docked SAM (light blue) within predicted ermA (violet) were superimposed in Figure 3. The observed differences in SAM orientation can be attributed to the presence of water molecules in the crystal, notably HOH-949 (Figure S1). The docking energies for SAM and selected flavonoids are summarized in Table S7.

The catechol groups of quercetin (**4**, green) and luteolin (**10**, purple) point toward the shallow groove and form hydrogen bonds (yellow dashed) with the backbone residues Phe-44 and Lys-41 as well as Nd1-His-43. Hydrophobic interactions, which are crucial for the binding affinity for the selected pocket, are mediated by ring A and ring C with additional H-bonds interacting with Asn-11 and the additional 3-hydroxyl group on the C-ring of quercetin interacting with Glu-59 (Figure S2).

**Figure 4:**
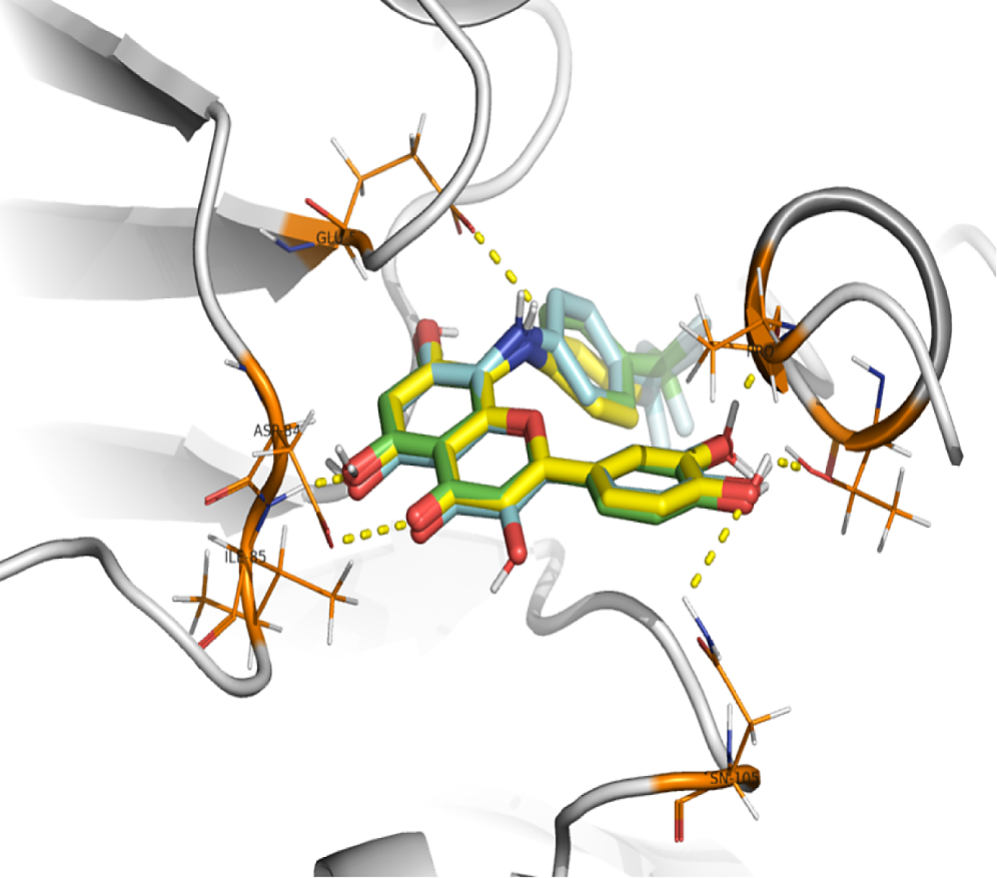
Docking of 8-(4-(trifluoromethyl)anilino)luteolin (**14**, light-blue), 8-(4-(trifluoromethyl)anilino)quercetin (**8**, green) and 8-(4-fluoroanilino)luteolin (**11**, yellow) in the shallow groove of methyltransferase.

Docking of 8-(4-(trifluoromethyl)anilino)luteolin (**14**, light-blue), 8-(4-(trifluoromethyl)anilino)quercetin (**8**, green) and 8-(4-fluoroanilino)luteolin (**11**, yellow) suggested that trifluoromethyl and fluoro substituents are buried in the shallow groove stabilizing the system by replacing H-bonds. Rings A and C occupy positions in the hydrophobic pocket at the end of the beta-sheet, forming H-bonds with Asp-84 and Ile-85, consistent with the adenosine interaction highlighted in the ErmC crystal structure (Figure 3). The catechol group forms an H-acceptor bond with *N*-Asn-105 at the loop connecting the α helix and beta-sheet, as well as an H-donor bond with Pro-6 and an H-donor toward Thr-9 backbone on *N*-terminal (Figure 4).

The docking results have shown the importance of the H-acceptor donor groups within the shallow groove. This was confirmed by the ability of quercetin and luteolin to mimic the stability of SAM-cysteine in the pocket by forming a network of H-bonds. In addition, hydrophobic interactions are mediated by the A and C rings of flavonoids coordinated by residues Asp-84 and Ile-85 providing SAM adenosine activity.

#### Mechanism of Gentamicin-Resistance Modulation in *S. aureus*

Among the mechanisms of resistance to gentamicin previously described in *S. aureus*, the presence of enzymes that alter the structure of aminoglycosides is crucial. These enzymes can transfer nucleotides, phosphates, or acetyls to various −OH or −NH_2_ groups of gentamicin, preventing its subsequent interaction with the cellular target, i.e., the 30S ribosomal subunit. The bifunctional aminoglycoside-modifying enzyme with acetylation and phosphotransferase activities encoded by the gene *aac(6’)/aph(2’)*, 4′,4″ adenyltransferase encoded by the *aadD* gene, and aminoglycoside *O*-phosphotransferase encoded by the gene *aph(3′)* are the most important.^37^ As can be seen in Table 5, the clinical isolate MRSA 9 possesses all three genes that may be responsible for the inactivation of gentamicin. For this reason, further testing continued using strains MRSA 3596 and MRSA 1584, which inactivate gentamicin only by a single mechanism (*aadD* and *aac(6’)-aph(2’’)*, respectively).

**Table 5:** Characterization of the Mechanism of Resistance to Gentamicin in Clinical Isolates of *S. aureus*

antibiotic	gene of resistance	MRSA 9	MRSA 3596	MRSA 1584
gentamicin	*aac(6′)-aph(2″)*	positive	negative	positive
	*aadD*	positive	positive	negative
	*aph(3′)*	positive	negative	negative

The mechanism was determined based on the presence/absence of the gene responsible for antibiotic modification in both plasmid and genomic DNA using qPCR.

All derivatives prepared exhibited synergistic activity against MRSA strain 3596, which uses adenyltransferases (encoded by the *aadD* gene) to eliminate antibiotics (Table S6). Surprisingly, according to the literature, gentamicin is not a substrate of the adenyltransferase,^38^ which was confirmed by evaluating the effect of gentamicin on the adenyltransferase. Therefore, the flavonoids affect the gentamicin resistance of *S. aureus* via an unknown mechanism that is still under investigation.

## Conclusion

In summary, we have prepared a library of novel derivatives of quercetin and luteolin modified at C-8 with various substituted anilines. The anti-inflammatory activity vanished after the introduction of the aniline group, while the cytotoxicity of the prepared derivatives remained low. Among the prepared derivatives, 8-(4-(trifluoromethyl)anilino) quercetin (**8**) and 8-(4-methoxyanilino) luteolin (**14**) showed the ability to modulate erythromycin resistance of *Staphylococcus aureus* by inhibiting ribosomal methyltransferase. Furthermore, the prepared derivatives acted as inhibitors of transmembrane efflux pumps at lower concentrations (25 and 50 μM) than the positive control CCCP and the parent compounds. All derivatives effectively modulated gentamicin resistance in gentamicin-resistant *Staphylococcus aureus*, although the underlying mechanism remains to be elucidated. Our findings underscore the potential of introducing amino groups into flavonoid structures to enhance their ability to combat drug resistance. According to our findings, the introduction of the anilino-substituents led to a certain focusing of the ability to modulate the MDR, which confirms the correctness of our approach. These derivatives hold promise for modulating drug resistance in adjuvant therapy. However, further investigation is needed to fully understand the specificity of these inhibitors against similar enzymes in other bacterial genera. Overall, our study contributes insights into the development of flavonoid-based therapeutics for combating antibiotic resistance, opening avenues for future research in this important field.

## Experimental Section

### General Information

Procedures involving oxygen- or moisture-sensitive materials were performed with anhydrous solvents (*vide infra*) under an argon atmosphere in flame-dried flasks using the Schlenk standard technique. Analytical TLC was performed on Al plates (Silica Gel 60 F_254_; Merck, Darmstadt, Germany). Purification was performed using the preparative HPLC system (Shimadzu, Kyoto, Japan). Preparative HPLC separations were performed using an ASAHIPAK GS-310 column (Shodex, Munich, Germany) at 5 mL/min MeOH (isocratic). NMR analyses were performed using spectrometers Bruker Avance III HD 500 MHz equipped with a cryoprobe (^1^H 500, ^13^C{1H} 126, and ^19^F 470 MHz), and Bruker Avance III 400 MHz (^1^H 400, ^13^C{1H} 100, and ^19^F 376 MHz) instrument (Bruker, Karlsruhe, Germany) in DMSO-*d*_6_, at 25 °C. The signal in DMSO-*d*_6_ was used as a reference (δ_H_ 2.50, δ_C_ 39.52). High-resolution mass spectra (HRMS) were measured using an LTQ Orbitrap XL hybrid mass spectrometer (Thermo Fisher Scientific, Waltham, MA, USA) equipped with an electrospray ion source and operated at the resolution of 100,000. Samples were loop-injected into methanol/water (4:1) at a flow rate of 100 μL/min. 8-Bromo-3,3′,4′,5,7-penta-*O*-isopropoxy quercetin and 8-bromo-3′,4′,5,7-tetra-*O*-isopropoxy luteolin were prepared according to a previously published method for selective bromination.^26^ Commercially available reagents and ligands were purchased from Sigma-Aldrich (Darmstadt, Germany), Alfa Aesar (Ward Hill, MA, USA), Acros Organics (Morris Plains, NJ, USA), and TCI Chemicals (Gurugram, India) and were used without further purification unless otherwise stated.

### General Procedure for Buchwald-Hartwig Amination

The respective brominated flavonoid (1 equiv) was dissolved in dry degassed THF (2 mL) and added to Pd_2_(dba)_3_ (3 mol %), tBuXPhos (6 mol %), and NaO*t*Bu (1.5 equiv) in a microwave vessel equipped with a stir bar under inert gas. The corresponding amine (1.2 equiv) was added. The reaction mixture was irradiated in a microwave reactor at 70 °C for 2 h. The reaction mixture was then allowed to cool to room temperature. The reaction mixture was filtered through a microfilter (PTFE, 0.45 μm), and washed with water (3 × 20 mL) and brine (3 × 20 mL). The combined organic fractions were dried over Na_2_SO_4_, evaporated *in vacuo,* and the residue was dissolved in dry dichloromethane (2 mL). The reaction mixture was cooled to 0 °C and BCl_3_ (1 M, 10 equiv) was added dropwise. The reaction mixture was then heated to 40 °C and stirred for 2 h, then cooled to 0 °C, and an excess of methanol was added. The reaction mixture was evaporated *in vacuo*, and the residue was purified by preparative HPLC chromatography (ASAHIPAK, 5 mL/min, MeOH isocratic) yielding the corresponding product.

#### 6-(Hexylamino)-2-phenyl-4*H*–1-benzopyran-4-one (6-(Hexylamino) flavone, **2**)

A yellow solid (62 mg, 60%). ^1^H NMR (500 MHz, DMSO-*d*_6_) δ 8.08–8.03 (m, 2H, H-2′, 6′), 7.60–7.53 (m, 4H, H-8, H-3′, 4′, 5′), 7.13 (dd, *J* = 9.1, 2.9 Hz, 1H, H-7), 6.94 (d, *J* = 2.9 Hz, 1H, H-5), 6.91 (s, 1H, H-3), 6.07 (t, *J* = 5.4 Hz, 1H, NH), 3.09–2.98 (m, 2H, CH_2_–1″), 1.58 (p, *J* = 7.2 Hz, 2H, CH_2_–2″), 1.44–1.34 (m, 2H, CH_2_–3″), 1.36–1.25 (m, 4H, CH_2_–4″,5″), 0.91–0.85 (m, 3H, CH_3_-6″) ppm. ^13^C{^1^H} NMR (126 MHz, DMSO-*d*_6_) δ 177.2 (C-4), 161.6 (C-2), 147.9 (C-9), 146.9 (C-6), 131.6 (C-1′), 131.4 (C-4′), 129.1 (2C, C-3′, 5′), 126.1 (2C, C-2′, 6′), 124.3 (C-10), 121.1 (C-7), 119.1 (C-8), 105.8 (C-3), 101.1 (C-5), 43.1 (CH_2_–1″), 31.1 (CH_2_–4″), 28.4 (CH_2_–2″), 26.4 (CH_2_–3″), 22.2 (CH_2_–5″), 14.0 (CH_3_-6″) ppm. HRMS (ESI, *m*/*z*) calcd for C_21_H_22_O_2_N [M – H]^−^ 320.16560, found 320.16534.

#### 6-(4-Methoxyanilino)-2-phenyl-4*H*–1-benzopyran-4-one (6-(4-Methoxyanilino) flavone, **3**)

A yellow solid (88.4 mg, 78%). ^1^H NMR (500 MHz, DMSO-*d*_6_) δ 8.23 (s, 1H, NH), 8.09–8.05 (m, 2H, H-2′, 6′), 7.65 (d, *J* = 9.0 Hz, 1H, H-8), 7.62–7.55 (m, 3H, H-3′, 4′, 5′), 7.42 (d, *J* = 2.9 Hz, 1H, H-5), 7.36 (dd, *J* = 9.0, 2.9 Hz, 1H, H-7), 7.14–7.10 (m, 2H, H-2″, 6″), 6.96–6.92 (m, 3H, H-3″, 5″ and H-3), 3.75 (s, 3H, OCH_3_) ppm. ^13^C{^1^H} NMR (126 MHz, DMSO-*d*_6_) δ 177.0 (C-4), 161.9 (C-2), 154.7 (C-4″), 149.1 (C-9), 143.5 (C-6), 135.2 (C-1″), 131.6 (C-4′), 131.5 (C-1′), 129.1 (2C, C-3′, 5′), 126.2 (2C, C-2′, 6′), 124.2 (C-10), 122.7 (C-7), 121.7 (2C, C-2″, 6″), 119.6 (C-8), 114.8 (2C, C-3″, 5″), 106.0 (C-3), 104.8 (C-5), 55.3 (OCH_3_) ppm. HRMS (ESI, *m*/*z*) calcd for C_22_H_16_O_3_N [M – H]^−^ 342.11357, found 342.11322.

#### 2-(3,4-Dihydroxyphenyl)-8-(4-fluoroanilino)-3,5,7-trihydroxy-4*H*–1-benzopyran-4-one (8-(4-Fluoroanilino) quercetin, **5**)

A green solid (57.4 mg, 54%). ^1^H NMR (500 MHz, DMSO-*d*_6_) δ 12.40 (s, 1H, HO-5), 10.60 (s, 1H, HO-7), 9.58 (s, 1H, HO-4′), 9.42 (s, 1H, HO-3), 9.07 (s, 1H, HO-3′), 7.63 (d, 1H, *J* = 2.3 Hz, H-2′), 7.10 (s, 1H, -NH), 6.96 (dd, 1H, *J* = 8.5, 2.2 Hz, H-6′), 6.94–6.88 (m, 2H, H-3″,5″), 6.64 (d, 1H, *J* = 8.5 Hz, H-5′), 6.58–6.52 (m, 2H, H-2″,6″), 6.35 (s, 1H, H-6) ppm. ^13^C{^1^H} NMR (126 MHz, DMSO-*d*_6_) δ 176.1 (C-4), 160.5 (C-7), 157.3 (C-5), 155.0 (d, *J* = 231.9 Hz, C-4″), 151.5 (C-9), 147.6 (C-4′), 146.7 (C-2), 144.8 (C-3′), 144.1 (dm, *J* = 1.8 Hz, C-1″), 135.7 (C-3), 122.0 (C-1′), 119.3 (C-6′), 115.8 (C-2′), 115.2 (C-5′), 115.1 (d, 2C, *J* = 22.1 Hz, C-3″,5″), 114.2 (d, 2C, *J* = 7.5 Hz, C-2″,6″), 108.3 (C-8), 103.3 (C-10), 98.1 (C-6) ppm. ^19^F NMR (470 MHz, DMSO-*d*_6_) δ −128.28 ppm. HRMS (ESI, *m*/*z*) calcd for C_21_H_13_O_7_F [M – H]^−^ 410.06815, found 410.06781.

#### 2-(3,4-Dihydroxyphenyl)-8-(4-methoxyanilino)-3,5,7-trihydroxy-4*H*–1-benzopyran-4-one (8-(4-Methoxyanilino) quercetin, **6**)

A brown solid (11.8 mg, 16%). ^1^H NMR (500 MHz, DMSO-*d*_6_) δ 12.40 (s, 1H, HO-5), 10.50 (s, 1H, HO-7), 9.56 (s, 1H, HO-4′), 9.39 (bs, 1H, HO-3), 9.07 (s, 1H, HO-3′), 7.65 (d, 1H, *J* = 2.2 Hz, H-2′), 6.90 (dd, 1H, *J* = 8.5, 2.3 Hz, H-6′), 6.77 (bs, 1H, NH), 6.75–6.69 (m, 2H, H-3″, 5′′), 6.61 (d, 1H, *J* = 8.5 Hz, H-5′), 6.56–6.51 (m, 2H, H-2″, 6′′), 6.34 (s, 1H, H-6), 3.63 (s, 3H, OCH_3_) ppm. ^13^C{^1^H} NMR (126 MHz, DMSO-*d*_6_) δ 176.1 (C-4), 160.3 (C-7), 157.0 (C-5), 151.7 (C-4″), 151.4 (C-9), 147.6 (C-4′), 146.7 (C-2), 144.7 (C-3′), 141.4 (C-1″), 135.6 (C-3), 122.0 (C-1′), 119.5 (C-6′), 115.8 (C-2′), 115.2 (C-5′), 114.5 (2C, C-2″,6′′), 114.4 (2C, C-3″,5″), 109.2 (C-8), 103.3 (C-10), 98.0 (C-6), 55.4 (OCH_3_) ppm. HRMS (ESI, *m*/*z*) calcd for C_22_H_16_O_8_N [M – H]^−^ 422.08814, found 422.08784.

#### 2-(3,4-Dihydroxyphenyl)-8-(anilino)-3,5,7-trihydroxy-4*H*–1-benzopyran-4-one (8-(Anilino) quercetin, **7**)

A yellow solid (42.7 mg, 64%). ^1^H NMR (500 MHz, DMSO-*d*_6_) δ 12.41 (s, 1H, HO-5), 10.58 (s, 1H, HO-7), 9.56 (bs, 1H, HO-4′or HO-3′), 9.41 (s, 1H, HO-3), 9.07 (bs, 1H, HO-4′or HO-3′), 7.66 (d, *J* = 2.2 Hz, 1H, H-2′), 7.13–7.05 (m, 2H, H-3″, 5″), 6.92 (dd, *J* = 8.5, 2.2 Hz, 1H, H-6′), 6.64–6.54 (m, 4H, H-2″, 6″, 4″, 5′), 6.36 (s, 1H, H-6) ppm. ^13^C{^1^H} NMR (126 MHz, DMSO-*d*_6_) δ 176.6 (C-4), 161.1 (C-7), 157.8 (C-5), 151.2 (C-9), 148.0 (C-4′), 147.2 (C-2), 145.2 (C-3′), 136.1 (C-3), 129.2 (2C, C-3″, 5″), 122.5 (C-1′), 119.9 (C-6′), 117.5 (C-4″), 116.3 (C-2′), 115.7 (C-5′), 113.8 (2C, C-2″, 6″), 108.5 (C-8), 103.8 (C-10), 98.0 (C-6) ppm. HRMS (ESI, *m*/*z*) calcd for C_21_H_14_O_7_N [M – H]^−^ 392.07758, found 392.07719.

#### 2-(3,4-Dihydroxyphenyl)-8-(4-(trifluoromethyl)anilino)-3,5,7-trihydroxy-4*H*–1-benzopyran-4-one (8-(4-(Trifluoromethyl)anilino) quercetin, **8**)

A yellow solid (64 mg, 41%). ^1^H NMR (500 MHz, DMSO-*d*_6_) δ 12.45 (s, 1H, HO-5), 10.79 (bs, 1H, HO-7), 9.58 (bs, 1H, HO-4′), 9.46 (bs, 1H, HO-3), 9.10 (bs, 1H, HO-3′), 7.84 (bs, 1H, NH), 7.66 (d, *J* = 2.2 Hz, 1H, H-2′), 7.40 (dm, *J* = 8.5 Hz, 2H, H-3″, 5″), 6.86 (dd, *J* = 8.5, 2.2 Hz, 1H, H-6′), 6.68 (dm, *J* = 8.3 Hz, 2H, H-2″, 6″), 6.59 (d, *J* = 8.5 Hz, 1H, H-5′), 6.38 (s, 1H, H-6) ppm. ^13^C NMR{^1^H} (126 MHz, DMSO-*d*_6_) δ 176.1 (C-4), 160.6 (C-7), 157.9 (C-5), 151.6 (C-9), 150.8 (C-1″), 147.6 (C-3′), 146.8 (C-2), 144.8 (C-4′), 135.8 (C-3), 126.1 (q, *J* = 3.8 Hz, 2C, C-3″, 5″), 125.3 (q, *J* = 270.1 Hz, CF_3_), 121.9 (C-1′), 119.2 (C-6′), 116.7 (q, *J* = 31.8 Hz, C-4″), 115.8 (C-2′), 115.1 (C-5′), 112.8 (2C, C-2″, 6″), 106.5 (C-8), 103.3 (C-10), 98.2 (C-6) ppm. ^19^F NMR (470 MHz, DMSO-*d*_6_) δ −58.99 ppm. HRMS (ESI, *m*/*z*) calcd for C_22_H_13_O_7_NF_3_ [M – H]^−^ 460.06496, found 460.06474.

#### 2-(3,4-Dihydroxyphenyl)-8-(3-fluoroanilino)-3,5,7-trihydroxy-4*H*–1-benzopyran-4-one (8-(3-Fluoroanilino) quercetin, **17***)*

A yellow solid (80 mg, 40%). ^1^H NMR (500 MHz, DMSO-*d*_6_) δ 12.43 (s, 1H, HO-5), 10.70 (bs, 1H, HO-7), 9.59 (bs, 1H, HO-4′), 9.45 (bs, 1H, HO-3), 9.09 (bs, 1H. HO-3′), 7.65 (d, *J* = 2.2 Hz, 1H, H-2′), 7.46 (bs, 1H, NH), 7.08 (m, 1H, H-5″), 7.01 (dd, *J* = 8.5, 2.3 Hz, 1H, H-6′), 6.64 (d, *J* = 8.5 Hz, 1H, H-5′), 6.42–6.37 (m, 2H, H-4″, 6″), 6.36 (s, 1H, H-6), 6.29 (dt, *J* = 12.1, 2.4 Hz, 1H, H-2″) ppm. ^13^C{^1^H} NMR (126 MHz, DMSO-*d*_6_) δ 176.1 (C-4), 163.3 (d, *J* = 239.6 Hz, C-3″), 160.6 (C-7), 157.7 (C-5), 151.7 (C-9), 149.8 (d, *J* = 10.9 Hz, C-1″), 147.6 (C-4′), 146.7 (C-2), 144.8 (C-3′), 135.7 (C-3), 130.2 (d, *J* = 10.1 Hz, C-5″), 122.0 (C-1′), 119.3 (C-6′), 115.8 (C-2′), 115.2 (C-5′), 109.3 (C-6″), 107.2 (C-8), 103.3 (C-10), 103.1 (d, *J* = 21.4 Hz, C-4″), 99.6 (d, *J* = 25.2 Hz, C-2″), 98.1 (C-6) ppm. ^19^F NMR (470 MHz, DMSO-*d*_6_) δ −128.18 ppm. HRMS (ESI, *m*/*z*) calcd for C_21_H_13_O_7_NF [M – H]^−^ 410.06815, found 410.06790.

#### 2-(3,4-Dihydroxyphenyl)-8-(3,5-dimethoxyanilino)-3,5,7-trihydroxy-4*H*–1-benzopyran-4-one (8-(3,5-Dimethoxyanilino) quercetin, **9**)

A yellow solid (41.4 mg, 27%). ^1^H NMR (500 MHz, DMSO-*d*_6_) δ 12.41 (s, 1H, HO-5), 10.57 (bs, 1H, HO-7), 9.60 (s, 1H, HO-4′), 9.42 (s, 1H, HO-3), 9.09 (s, 1H, HO-3′), 7.69 (d, *J* = 2.2 Hz, 1H, H-2′), 7.11 (bs, 1H, NH), 7.07 (dd, *J* = 8.5, 2.2 Hz, 1H, H-6′), 6.66 (d, *J* = 8.5 Hz, 1H, H-5′), 6.34 (s, 1H, H-6), 5.83 (tm, *J* = 2.2 Hz, 1H, H-4″), 5.76 (dm, *J* = 2.2 Hz, 2H, H-2″, 6″), 3.60 (s, 6H, OCH_3_) ppm. ^13^C{^1^H} NMR (126 MHz, DMSO-*d*_6_) δ 176.1 (C-4), 161.1 (2C, C-3″, 5″), 160.8 (C-7), 157.6 (C-5), 151.8 (C-9), 149.8 (C-1″), 147.6 (C-4′), 146.7 (C-2), 144.8 (C-3′), 135.7 (C-3), 122.1 (C-1′), 119.5 (C-6′), 115.9 (C-2′), 115.2(C-5′), 108.0 (C-8), 103.3 (C-10), 98.0 (C-6), 92.3 (2C, C-2″, 6″), 89.7 (C-4″), 54.8 (2C, OCH_3_) ppm. HRMS (ESI, *m*/*z*) calcd for C_23_H_18_O_9_N [M – H]^−^ 452.09870, found 452.09827.

#### 2-(3,4-Dihydroxyphenyl)-8-(4-fluoroanilino)-5,7-dihydroxy-4*H*–1-benzopyran-4-one (8-(4-Fluoroanilino) luteolin, **11**)

A yellow solid (95 mg, 64%). ^1^H NMR (500 MHz, DMSO-*d*_6_) δ 12.89 (s, 1H, HO-5), 10.70 (bs, 1H), 9.89 (bs, 1H), 9.13 (bs, 1H) (HO-3′, HO-4′, HO-7), 7.15 (d, *J* = 2.3 Hz, 1H, 2′), 7.15 (bs, 1H, -NH), 7.00 (dd, *J* = 8.4, 2.3 Hz, 1H, H-6′), 6.92 (m, 2H, H-3″, 5″), 6.70 (d, *J* = 8.4 Hz, 1H, H-5′), 6.65 (s, 1H, H-3), 6.62–6.54 (m, 2H, H-2″, 6″), 6.36 (s, 1H, H-6) ppm. ^13^C{^1^H} NMR (126 MHz, DMSO-*d*_6_) δ 182.0 (C-4), 163.7 (C-2), 160.8 (C-7), 158.1 (C-5), 155.0 (d, *J* = 232.0 Hz, C-4″), 152.6 (C-9), 149.7 (C-4′), 145.6 (C-3′), 143.9 (d, *J* = 1.4 Hz, C-1″), 121.5 (C-1′), 118.8 (C-6′), 115.5 (C-5′), 115.1 (d, *J* = 22.4 Hz, 2C, C-3″, 5″), 114.2 (d, *J* = 7.5 Hz, 2C, C-2″, 6″), 113.7 (C-2′), 108.8 (C-8), 104.0 (C-10), 102.6 (C-3), 98.7 (C-6) ppm. ^19^F NMR (470 MHz, DMSO-*d*_6_) δ −128.2 ppm. HRMS (ESI, *m*/*z*) calcd for C_21_H_13_O_6_NF [M – H]^−^ 394.07324, found 394.07290.

#### 2-(3,4-Dihydroxyphenyl)-8-(4-methoxyanilino)-5,7-dihydroxy-4*H*–1-benzopyran-4-one (8-(4-Methoxyanilino) luteolin, **12**)

A yellow solid (50 mg, 33%). ^1^H NMR (500 MHz, DMSO-*d*_6_) δ 12.86 (s, 1H, HO-5), 10.56 (bs, 1H), 9.97 (bs, 1H), 9.16 (bs, 1H) (OH-7, OH-3′, OH-4′), 7.17 (d, *J* = 2.3 Hz, 1H, H-2′), 6.94 (dd, *J* = 8.4, 2.3 Hz, 1H, H-6′), 6.81 (s, 1H, NH), 6.72 (m, 2H, H-3″, 5″), 6.68 (d, *J* = 8.4 Hz, 1H, H-5′), 6.63 (s, 1H, H-3), 6.56 (m, 2H, H-2″, 6″), 6.35 (s, 1H, H-6), 3.63 (s, 3H, OCH_3_) ppm. ^13^C{^1^H} NMR (126 MHz, DMSO-*d*_6_) δ 182.0 (C-4), 163.7 (C-2), 160.7 (C-7), 157.7 (C-5), 152.4 (C-9), 151.7 (C-4″), 149.7 (C-4′), 145.6 (C-3′), 141.3 (C-1″), 121.5 (C-1′), 118.9 (C-6′), 115.4 (C-5′), 114.6 (2C, C-2″, 6″), 114.4 (2C, C-3″, 5″), 113.7 (C-2′), 109.7 (C-8), 103.9 (C-10), 102.5 (C-3), 98.7 (C-6), 55.4 (OCH_3_) ppm. HRMS (ESI, *m*/*z*) calcd for C_22_H_16_O_7_N [M – H]^−^ 406.09323, found 406.09286.

#### 2-(3,4-Dihydroxyphenyl)-8-(anilino)-5,7-dihydroxy-4*H*–1-benzopyran-4-one (8-(Anilino) luteolin, **13**)

A yellow solid (51.1 mg, 36%). ^1^H NMR (500 MHz, DMSO-*d*_6_) δ 12.89 (bs, 1H), 10.69 (bs, 1H), 9.97 (bs, 1H), 9.14 (bs, 1H) (OH-5, OH-7, OH-3′, OH-4′), 7.95 (bs, 1H, NH), 7.18 (d, *J* = 2.3 Hz, 1H, H-2′), 7.08 (m, 2H, H-3″, 5″), 6.96 (dd, *J* = 8.4, 2.3 Hz, 1H, H-6′), 6.68 (d, *J* = 8.4 Hz, 1H, H-5′), 6.64 (s, 1H, H-3), 6.62 (dm, 1H, H-4″), 6.59 (m, 2H, H-2″, 6″), 6.38 (s, 1H, H-6) ppm. ^13^C{^1^H} NMR (126 MHz, DMSO-*d*_6_) δ 182.0 (C-4), 163.8 (C-2), 161.0 (C-7), 158.1 (C-5), 152.8 (C-9), 149.7 (C-4′), 147.4 (C-1″), 145.6 (C-3′), 128.8 (2C, C-3″, 5″), 121.5 (C-1′), 118.8 (C-6′), 117.1 (C-4″), 115.5 (C-5′), 113.7 (C-2′), 113.3 (2C, C-2″, 6″), 108.6 (C-8), 103.9 (C-10), 102.5 (C-3), 98.7 (C-6) ppm. HRMS (ESI, *m*/*z*) calcd for C_21_H_14_O_6_N [M – H]^−^ 376.08266, found 376.08234.

#### 2-(3,4-Dihydroxyphenyl)-8-(4-(trifluoromethyl)anilino)-5,7-dihydroxy-4*H*–1-benzopyran-4-one (8-(4-(Trifluoromethyl)anilino) luteolin, **14**)

Dark gray petals (85 mg, 51%). ^1^H NMR (500 MHz, DMSO-*d*_6_) δ 12.94 (bs, 1H, HO-5), 10.88 (bs, 1H, HO-7), 9.99 (bs, 1H, HO-4′), 9.16 (bs, 1H, HO-3′), 7.89 (s, 1H, NH), 7.45–7.37 (m, 2H, H-3″, 5″), 7.19 (d, *J* = 2.3 Hz, 1H, H-2′), 6.92 (dd, *J* = 8.4, 2.3 Hz, 1H, H-6′), 6.70 (m, 2H, H-2″, 6″), 6.67 (s, 2H, H-3) 6.67 (d, *J* = 8.4 Hz, H-5′), 6.39 (s, 1H, H-6) ppm. ^13^C{^1^H} NMR (126 MHz, DMSO-*d*_6_) δ 182.0 (C-4), 163.7 (C-2), 160.9 (C-7), 158.7 (C-5), 152.8 (C-9), 150.7 (C-1″), 149.8 (C-4′), 145.7 (C-3′), 126.2 (q, *J* = 3.9 Hz, 2C, C-3″, 5″), 125.3 (q, *J* = 270.0 Hz, CF_3_), 121.4 (C-1′), 118.7 (C-6′), 116.8 (q, *J* = 31.8 Hz, C-4″), 115.5 (C-5′), 113.7 (C-2′), 112.8 (2C, C-2″, 6″), 107.0 (C-8), 104.0 (C-10), 102.7 (C-3), 98.8 (C-6) ppm. ^19^F NMR (470 MHz, DMSO-*d*_6_) δ −59.00 ppm. HRMS (ESI, *m*/*z*) calcd for C_22_H_13_O_6_NF_3_ [M – H]^−^ 444.07004, found 444.06974.

#### 2-(3,4-Dihydroxyphenyl)-8-(3-fluoroanilino)-5,7-dihydroxy-4*H*–1-benzopyran-4-one (8-(3-Fluoroanilino) luteolin, **15**)

A yellow solid (89 mg, 60%). ^1^H NMR (500 MHz, DMSO-*d*_6_) δ 12.92 (s, 1H, HO-5), 10.79 (bs, 1H), 9.99 (bs, 1H), 9.16 (bs, 1H) (HO-7, HO-3′, HO-4′), 7.51 (s, 1H, NH), 7.19 (d, *J* = 2.3 Hz, 1H, H-2′), 7.09 (dm, 1H, H-5″), 7.05 (dd, *J* = 8.4, 2.3 Hz, 1H, H-6′), 6.71 (d, *J* = 8.4 Hz, 1H, H-5′), 6.67 (s, 1H, H-3), 6.41 (m, 2H, H-4″, 6″), 6.37 (s, 1H, H-6), 6.31 (dm, *J* = 12.0, 2.3 Hz, 1H, H-2″) ppm. ^13^C{^1^H} NMR (126 MHz, DMSO-*d*_6_) δ 182.0 (C-4), 163.7 (C-2), 163.3 (d, *J* = 239.8 Hz, C-3″), 160.9 (C-7), 158.5 (C-5), 152.8 (C-9), 149.7 (C-4′), 149.7 (d, *J* = 10.9 Hz, C-1″), 145.7 (C-3′), 130.2 (d, *J* = 10.1 Hz, C-5″), 121.5 (C-1′), 118.8 (C-6′), 115.5 (C-5′), 113.7 (C-2′), 109.3 (d, *J* = 1.7 Hz, C-6″), 107.7 (C-8), 104.0 (C-10), 103.1 (d, *J* = 21.3 Hz, C-4″), 102.6 (C-3), 99.7 (d, *J* = 25.1 Hz, C-2″), 98.8 (C-6) ppm. ^19^F NMR (470 MHz, DMSO-*d*_6_) δ −113.71 ppm. HRMS (ESI, *m*/*z*) calcd for C_21_H_15_O_6_NF [M + H]^+^ 396.08779, found 396.08757.

#### 2-(3,4-Dihydroxyphenyl)-8-(3,5-dimethoxyanilino)-5,7-dihydroxy-4*H*–1-benzopyran-4-one (8-(3,5-Dimethoxyanilino) luteolin, **16**)

A brown solid (15 mg, 9%). ^1^H NMR (500 MHz, DMSO-*d*_6_) δ 12.89 (s, 1H, HO-5), 10.76 (s, 1H), 10.00 (s, 1H) 9.15 (s, 1H) (OH-7, OH-3′, OH-4′), 7.24 (d, *J* = 2.3 Hz, 1H, H-2′), 7.16 (s, 1H, NH), 7.09 (dd, *J* = 8.4, 2.3 Hz, 1H, H-6′), 6.73 (d, *J* = 8.4 Hz, 1H, H-5′), 6.65 (s, 1H, H-3), 6.35 (s, 1H, H-6), 5.83 (tm, *J* = 2.2 Hz, 1H, H-4″), 5.77 (dm, *J* = 2.1 Hz, 2H, H-2″, 6″), 3.61 (s, 6H, −CH_3_) ppm. ^13^C{^1^H} NMR (126 MHz, DMSO-*d*_6_) δ 182.0 (C-4), 163.7 (C-2), 161.1 (C-7), 161.1 (C-3″, 5″), 158.3 (C-5), 152.9 (C-9), 149.7 (C-1″), 149.7 (C-4′), 145.6 (C-3′), 121.5 (C-1′), 118.9 (C-6′), 115.5 (C-5′), 113.8 (C-2′), 108.5 (C-8), 103.9 (C-10), 102.6 (C-3), 98.7 (C-6), 92.3 (2C, C-2″,6″), 89.8 (C-4″), 54.8 (OCH_3_) ppm. HRMS (ESI, *m*/*z*) calcd for C_23_H_18_O_8_N [M – H]^−^ 436.10379, found 436.10345.

### Biological Activity

2,2′-Azobis(2-methylpropionamidine) dihydrochloride (AAPH); 2′,7′-dichlorofluorescin diacetate (DCFH-DA); antibiotic antimycotic solution; Cefotaxime; Colistin; Dulbecco’s Modified Eagle’s Medium (DMEM); Erythromycin; Fetal Bovine Serum (FBS); Griess reagent; l-glutamine; lipopolysaccharides from *Escherichia coli* O111:B4 (LPS); MEM medium (Eagle’s Minimum Essential Media, no phenol red); Mueller Hinton Broth (MH); phosphate buffer saline (PBS); resazurin sodium salt; and trypsin/EDTA solution were purchased from Sigma-Aldrich (St. Louis, Missouri, USA).

### Cell Lines and Bacterial Strains

Murine macrophages (Raw, 264.7, Sigma-Aldrich), human keratinocytes (HaCaT, C0055C, Thermo Fisher Scientific, Waltham, MA, USA); human dermal fibroblasts (HDF, 106–05A, Sigma-Aldrich) were cultivated in DMEM supplemented with 10% FBS, 2 mM l-glutamine, and 1× antibiotic antimycotic solution. The cells were cultivated in a CO_2_ incubator (5% CO_2_, 37 °C, Thermo Fisher Scientific, Brno, Czech Republic) and passaged twice a week with trypsin/EDTA solution according to a standardized protocol (Table 6). The human cell cultures were periodically authenticated using short tandem repeat (STR) profiling and checked for mycoplasma presence.

**Table 6:** For the Genotypic Characterization of *Staphylococcus aureus* Strains, the Following Set of Primers and Methods was Used

gene	primer sequence (5′–3′)
*ermA*	GAAGCGGTAAACCCCTCTGA
	TCGCAAATCCCTTCTCAACGA
*ermB*	CCGAACACTAGGGTTGCTCT
	CATCTGTGGTATGGCGGGTA
*ermC*	ATCGGCTCAGGAAAAGGGC
	TTGGAAATTATCGTGATCAACAAGT
*msrA*	GAAGACATGCGTGACGTTTCA
	TCGTTCTTTCCCCACCACTC
*lmrS*	ATACTTAGCGGCGATGGGGA
	ATAAGTACGCCTGCACCCAT
*mphC*	TGGACTGAAGCAACCCACTC
	CGCCGATTCTCCTGATTCCA

Bacterial strains originated from the General University Hospital (Prague, Czech Republic) and their phenotypical characterization determined by the disc diffusion method according to EUCAST was as follows:

MRSA 9 resistant to PEN, OXA, MET, ERY, CLI, GEN, CIP, CMP, TET, AMI, TOB

MRSA 3596 resistant to PEN, OXA, MET, ERY, CLI, GEN, CIP, TET, RIF, AMI, TOB

MRSA 331 resistant to PEN, OXA, MET, ERY, CLI, CIP, CMP

MSSA 2 resistant to PEN (methicillin-sensitive penicillin-resistant *Staphylococcus aureus*)

where the abbreviations refer to antibiotics as follows: penicillin (PEN), oxacillin (OXA), methicillin (MET), erythromycin (ERY), clindamycin (CLI), gentamicin (GEN), ciprofloxacin (CIP), chloramphenicol (CMP), tetracyclin (TET), rifampicin (RIF), amikacin (AMI), tobramycin (TOB).

Antibiotic-sensitive strain of *S. aureus* CCM 4223 was purchased from the Czech Collection of Microorganisms (CCM, Brno, Czech Republic).

The overnight culture was diluted in MH broth to the final concentration of 2 × 10^8^ CFU/ml. Cell pellets were harvested by centrifugation (8000 × *g*, 5 min) and stored at −20 °C. Genomic DNA was isolated using PureLink Genomic DNA Mini Kit (Invitrogen, USA) with the addition of lysostaphin (100 mg/L) according to the manufacturer’s instructions. Likewise, plasmid DNA was isolated using the QIAprep Spin Miniprep Kit (Qiagen, Germany) with the addition of lysostaphin (100 mg/L) according to the manufacturer’s instructions.

The presence of the investigated genes in DNA was verified using the polymerase chain reaction (CFX96 Real-Time PCR Detection System, BIO-RAD, USA). The iQ SYBR Green Supermix was used in combination with the designed primers listed above. As part of the PCR method, mixtures of plasmid and genomic DNA were prepared for each strain. For the reaction itself, the starting concentration of the DNA mix was 1 μg/mL. The reaction was performed for each gene in duplicates. For each master mix, a nontemplate control was also measured, which had to be negative.

### Cellular Antioxidant Capacity

Cellular antioxidant capacity was evaluated as previously described.^31,39^ Briefly, 100 μL of RAW 264.7 cells with a density corresponding to 1 × 10^6^ cells/mL were split into 96-well plates. After 24 h, cells were washed with PBS, and DMEM supplemented with DCFH-DA (0.0125 mg/mL) was added to each well together with the tested samples in the concentration range of 1.5–200 μM. After 1 h incubation in a CO_2_ incubator, the medium was replaced with AAPH solution (0.16 mg/mL in PBS), and fluorescence was immediately recorded (ex./em. 485/540 nm) for 2 h in 5 min intervals.

### Anti-Inflammatory Activity

The anti-inflammatory activity of the tested flavonoids was determined as the ability to reduce the production of nitric oxide (NO), tumor necrosis factor (TNF-α), and interleukin 6 (IL-6) by RAW 264.7 stimulated by LPS, as previously described.^39,40^ Briefly, 1 × 10^6^ cells/mL was seeded into the 96-well plate (100 μL/well). After 48 h, LPS (100 ng/mL) and the samples (3–100 μM) were added to the MEM medium. After 24 h, the medium was mixed with Griess reagent (0.04 g/mL) at a 1:1 ratio. The absorbance was measured after 15 min at 540 nm. Cell viability was determined using the resazurin assay.

To determine TNF-α levels, cells were precultivated with the tested compounds for 24 h. After 2 h of incubation with LPS, the medium was diluted 1:10 with ELISA diluent. To determine the level of IL-6, cells were cultivated for 6 h with LPS and the tested compounds. Cytokine production was determined using an uncoated ELISA performed according to the manufacturer’s instructions.

### Cytotoxicity

100 μL of cell suspension containing 10^5^ cells/mL was pipetted into a 96-well plate. After 24 h incubation, the tested compounds were added in the concentration range 12.5–200 μM. After 72 h incubation, the cell viability was tested by standard resazurin assay.^41^ Briefly, the cells were washed with PBS and incubated with 100 μL of resazurin solution (0.025 mg/mL) for 2 h. The fluorescence was measured at a wavelength of 560 nm excitation/590 nm emission.

### Susceptibility of Antibiotic-Resistant Bacteria

Sensitization of multidrug-resistant clinical bacterial strains was performed according to ref^15^. Antibiotic cutoff concentration was chosen according to the European Committee on Antimicrobial Susceptibility Testing (EUCAST, Clinical breakpoints–bacteria, ver. 11.0). The 200 μM concentration of the derivatives was chosen as the highest test concentration. The absorbance detected at 600 nm was then used to estimate the number of viable cells after 24 h of incubation at 37 °C and 120 rpm.

### Ethidium Bromide Accumulation Assay

The accumulation assay was performed according to previously published methods with minor modifications.^15,42^ Briefly, a bacterial culture of *S. aureus* MSSA 2 cultured overnight in the presence of colistin (4 mg/L) to induce oxidative stress and activate efflux system^34^ was diluted in PBS to OD_600 nm_ = 0.6. The suspension was centrifuged (13,000 × *g*; 3 min), resuspended in fresh PBS, and distributed into the wells of a black 96-well microtiter plate (ThermoFisher Scientific). Samples were diluted with EtBr solution (4 μg/mL). 50 μL of the mixture was added to 50 μL of the bacterial suspension. The reaction was monitored using SpectraMax iD5Multi-Mode Microplate Reader (ex/em = 530/600 nm) every 60 s for 60 min.

### Molecular Docking Analyses

AutoDock Tools was also employed to generate the necessary grid for conducting molecular docking with VinaDock 1.2.^43^ First, the search for the optimal binding pose focused on the region based on a priori knowledge, for exploring ligand–receptor interactions. Subsequently, blind molecular docking was performed to investigate optional pockets of potential interest.

The protein MLS_B (also known as ERM_B) was predicted using the artificial intelligence program AlphaFold. Following the studies by Lee et al.,^44^ the N-terminal portion (NTERM) was identified as the region of primary interest for the activities of proteins encoded by ERMs. The following approach was used to construct the spatial grid box the region of interest was identified between amino acids 8TQNF11, delimited within the region defined as the X-motif. Validation of this area was performed by docking the molecule *S*-adenosyl-l-methionine (SAM), and the results were subsequently compared with those obtained by cocrystallization of SAM with the MLS protein encoded by the ERM C gene (PDB: 1QAN).

For visualization purposes and to calculate the electron density function, PyMOL Molecular Graphics System, Version 2.0 by Schrödinger, LLC, was employed.

### Statistical Analysis

Experiments were performed with the respective number of replicates indicated in each figure/table. MIC was determined as IC_70_. Both IC_70_ and IC_50_ values were calculated using the following nonlinear regression in the software GraphPad Prism:

Y= Bottom + (Top – Bottom)/(1 + ((Top – Bottom)/((Top + Baseline)/2 – Bottom) – 1) * (Absolute IC_50_/X) ^ HillSlope)

where IC_70_ was calculated by the transformation of best-fit parameters defined as X.

Data are presented as mean values of replicates with the standard error of the mean (SEM). Statistical significance was tested with a Students *t*-test.

## Supporting Information Available

The results of optimization of Ullmann reaction, cellular antioxidant and anti-inflammatory activity, cytotoxicity, antibacterial activity, and molecular docking, ^1^H, ^13^C{^1^H} NMR data, HPLC, and HRMS analyses is available free of charge on the ACS Publications Web site (PDF)
