# Supplementary material for: Nitrogen-Containing Flavonoids—Preparation and Biological Activity
Source: ACS Omega. 2024 Jul 29;9(32):34938–50. doi: 10.1021/acsomega.4c04627 (PMC11325505; doi:10.1021/acsomega.4c04627)
Supplement: Supplementary file 1 — ao4c04627_si_001.pdf [file ao4c04627_si_001.pdf]

*Supporting Information*

# Nitrogen-Containing Flavonoids – Preparation and Biological Activity

Martina Hurtová*,[†] Daniela Brdová,[‡] Bára Křížkovská,[‡] Guglielmo Tedeschi,[‡] Tomáš Nejedlý,[‡] Ondřej Strnad,[‡] Simona Dobiasová,[‡] Zuzana Osifová,[¶] Gabriela Kroneislová,[‡,§] Jan Lipov,[‡] Kateřina Valentová,[†] Jitka Viktorová,[‡] Vladimír Křen[†]

[†]Institute of Microbiology of the Czech Academy of Sciences, Vídeňská 1083, 142 00 Prague, Czech Republic

[‡]Department of Biochemistry and Microbiology, University of Chemistry and Technology Prague, Technická 5, 166 28 Prague, Czech Republic

[¶]Institute of Organic Chemistry and Biochemistry of the Czech Academy of Sciences, Flemingovo nám. 542, 160 00 Prague, Czech Republic

[§]Department of Clinical Microbiology and ATB Center, Institute of Medical Biochemistry and Laboratory Diagnostics of the General University Hospital and of The First Faculty of Medicine of Charles University, U Nemocnice 2, 128 08 Prague 2, Czech Republic

# Contents

**1. CHEMISTRY** .......... 5

**Table S1**. An overview of unsuccessful (no reaction) methods used for the Ullman reaction.. 5

**2. BIOLOGICAL ACTIVITY** .......... 6

**Table S2**. Cellular antioxidant activity (CAA) activity of quercetin, luteolin, and their derivatives. .......... 6

**Table S3**. Anti-inflammatory activity of quercetin, luteolin, and their derivatives .......... 7

**Table S4**. Cytotoxicity of the prepared derivatives .......... 8

**Table S5**. Antibacterial activity of quercetin (4), luteolin (10), and their derivatives .......... 9

**Table S6**. Mode of antimicrobial action of quercetin, luteolin and their derivatives .......... 10

**Table S7**. Docking into the model of ribosomal methyltransferase .......... 11

**Figure S1**. The observed differences in SAM orientation. .......... 12

**Figure S2**. Docking of quercetin (**4**) and luteolin (**10**) into methyltrasferase. .......... 12

**3. NMR SPECTRA** .......... 13

**Figure S3**. $^{1}$H NMR spectrum of 6-hexylamino flavone (**2**). .......... 13

**Figure S4**. $^{13}$C{$^{1}$H} APT NMR spectrum of 6-hexylamino flavone (**2**) .......... 13

**Figure S5**. $^{1}$H NMR spectrum of 6-(4-methoxyanilino) flavone (**3**). .......... 14

**Figure S6**. $^{13}$C{$^{1}$H} APT NMR spectrum of 6-(4-methoxyanilino) flavone (**3**) .......... 14

**Figure S7**. $^{1}$H NMR spectrum of 8-(4-fluoroanilino) quercetin (**5**) .......... 15

**Figure S8**. $^{13}$C{$^{1}$H} APT NMR spectrum of 8-(4-fluoroanilino) quercetin (**5**) .......... 15

**Figure S9**. $^{19}$F NMR spectrum of 8-(4-fluoroanilino) quercetin (**5**). .......... 16

**Figure S10**. $^{1}$H NMR spectrum of 8-(4-methoxyanilino) quercetin (**6**) .......... 17

**Figure S11**. $^{13}$C{$^{1}$H} APT NMR spectrum of 8-(4-methoxyanilino) quercetin (**6**). .......... 17

**Figure S12**. $^{1}$H NMR spectrum of 8-(anilino) quercetin (**7**). .......... 18

**Figure S13**. $^{13}$C{$^{1}$H} APT NMR spectrum of 8-(anilino) quercetin (**7**). .......... 18

**Figure S14**. $^{1}$H NMR spectrum of 8-(4-(trifluoromethyl)anilino) quercetin (**8**). .......... 19

**Figure S15**. $^{13}$C{$^{1}$H} APT NMR spectrum of 8-(4-(trifluoromethyl)anilino) quercetin (**8**). .. 19

**Figure S16**. $^{19}$F NMR spectrum of 8-(4-(trifluoromethyl)anilino) quercetin (**8**). .......... 20

**Figure S17**. $^{1}$H NMR spectrum of 8-(3-fluoroanilino) quercetin (**17**) .......... 21

**Figure S18**. $^{13}$C{$^{1}$H} APT NMR spectrum of 8-(3-fluoroanilino) quercetin (**17**). .......... 21

**Figure S19**. $^{19}$F NMR spectrum of 8-(3-fluoroanilino) quercetin (**17**). .......... 22

**Figure S20**. $^{1}$H NMR spectrum of 8-(3,5-dimethoxyanilino) quercetin (**9**). .......... 23

**Figure S21**. $^{13}$C{$^{1}$H} APT NMR spectrum of 8-(3,5-dimethoxyanilino) quercetin (**9**). .......... 23

**Figure S22**. $^{1}$H NMR spectrum of 8-(4-fluoroanilino) luteolin (**11**). .......... 24

**Figure S23**. $^{13}$C{$^{1}$H} APT NMR spectrum of 8-(4-fluoroanilino) luteolin (**11**) .......... 24

**Figure S24**. $^{19}$F NMR spectrum of 8-(4-fluoroanilino) luteolin (**11**). .......... 25

**Figure S25**. $^{1}$H NMR spectrum of 8-(4-methoxyanilino) luteolin (**12**)................................. 26
**Figure S26**. $^{13}$C{$^{1}$H} APT NMR spectrum of 8-(4-methoxyanilino) luteolin (**12**)................ 26
**Figure S27**. $^{1}$H NMR spectrum of 8-(anilino) luteolin (**13**). ................................................ 27
**Figure S28**. $^{13}$C{$^{1}$H} APT NMR spectrum of 8-(anilino) luteolin (**13**) ................................ 27
**Figure S29**. $^{1}$H NMRspectrum of 8-(4-(trifluoromethyl)anilino) luteolin (**14**)...................... 28
**Figure S30**. $^{13}$C{$^{1}$H} APT NMR spectrum of 8-(4-(trifluoromethyl)anilino) luteolin (**14**).... 28
**Figure S31**. $^{19}$F NMR spectrum of 8-(4-(trifluoromethyl)anilino) luteolin (**14**). ................... 29
**Figure S32**. $^{1}$H NMR spectrum of 8-(3-fluoroanilino) luteolin (**15**). .................................... 30
**Figure S33**. $^{13}$C{$^{1}$H} APT NMR spectrum of 8-(3-fluoroanilino) luteolin (**15**). ................... 30
**Figure S34**. $^{19}$F NMR spectrum of 8-(3-fluoroanilino) luteolin (**15**). ................................... 31
**Figure S35**. $^{1}$H NMR spectrum of 8-(3,5-dimethoxyanilino) luteolin (**16**)........................... 32
**Figure S36**. $^{13}$C{$^{1}$H} APT NMR spectrum of 8-(3,5-dimethoxyanilino) luteolin (**16**).......... 32
**4. HPLC ANALYSES** ........................................................................................ 33
**Figure S37**. HPLC chromatogram of 6-hexylamino flavone (**2**)........................................... 33
**Figure S38**. HPLC chromatogram of 8-(4-methoxyanilino) flavone (**3**) ............................... 33
**Figure S39**. HPLC chromatogram of 8-(4-fluoroanilino) quercetin (**5**)................................. 34
**Figure S40**. HPLC chromatogram of 8-(4-methoxyanilino) quercetin (**6**) ............................. 34
**Figure S41**. HPLC chromatogram of 8-(anilino) quercetin (**7**)............................................. 35
**Figure S42**. HPLC chromatogram of 8-(4-(trifluoromethyl)anilino) quercetin (**8**) ................ 35
**Figure S43**. HPLC chromatogram of 8-(3-fluoromethylanilino) quercetin (**17**).................... 36
**Figure S44**. HPLC chromatogram of 8-(3,5-dimethoxyanilino) quercetin (**9**) ...................... 36
**Figure S45**. HPLC chromatogram of 8-(4-fluoroanilino) luteolin (**11**) ................................. 37
**Figure S46**. HPLC chromatogram of 8-(4-methoxyanilino) luteolin (**12**) ............................. 37
**Figure S47**. HPLC chromatogram of 8-(anilino) luteolin (**13**)............................................... 38
**Figure S48**. HPLC chromatogram of 8-(4-(trifluoromethyl)anilino) luteolin (**14**)................ 38
**Figure S49**. HPLC chromatogram of 8-(3-fluoroanilino) luteolin (**15**) ................................. 39
**Figure S50**. HPLC chromatogram of 8-(3,5-dimethoxyanilino) luteolin (**16**)........................ 39
**5. HRMS**............................................................................................................ 40
**Figure S51**. HRMS (ESI$^-$) analysis of 6-hexylamino flavone (**2**) .......................................... 40
**Figure S52**. HRMS (ESI$^-$) analysis of 6-(4-methoxyanilino) flavone (**3**).............................. 40
**Figure S53**. HRMS (ESI$^-$) analysis of 8-(4-fluoroanilino) quercetin (**5**) ............................... 41
**Figure S54**. HRMS (ESI$^-$) analysis of 8-(4-methoxyanilino) quercetin (**6**) ........................... 41
**Figure S55**. HRMS (ESI$^-$) analysis of 8-(anilino) quercetin (**7**)............................................. 42
**Figure S56**. HRMS (ESI$^-$) analysis of 8-(4-(trifluoromethyl)anilino) quercetin (**8**) ............... 42
**Figure S57**. HRMS (ESI$^-$) analysis of 8-(3-fluoroanilino) quercetin (**17**) ............................. 43
**Figure S58**. HRMS (ESI$^-$) analysis of 8-(3,5-dimethoxyanilino) quercetin (**9**) ..................... 43

**Figure S59**. HRMS (ESI$^-$) analysis of 8-(4-fluoroanilino) luteolin (**11**). ................................ 44
**Figure S60**. HRMS (ESI$^-$) analysis of 8-(4-methoxyanilino) luteolin (**12**)............................. 44
**Figure S61**. HRMS (ESI$^-$) analysis of 8-(anilino) luteolin (**13**) .............................................. 45
**Figure S62**. HRMS (ESI$^-$) analysis of 8-(4-(trifluoromethyl)anilino) luteolin (**14**)................ 45
**Figure S63**. HRMS (ESI$^-$) analysis of 8-(3-fluoroanilino) luteolin (**15**)................................. 46
**Figure S64**. HRMS (ESI$^-$) analysis of 8-(3,5-dimethoxyanilino) luteolin (**16**)....................... 46

## 1. CHEMISTRY

**Table S1.** An overview of unsuccessful (no reaction) methods used for the Ullman reaction of halogen flavonoids with alkyl amines and amino acids. 8-Iodo-3,3′,4′,5,7-penta-*O*-isopropoxy quercetin (8-I) and 8-bromo-3,3′,4′,5,7-penta-*O*-isopropoxy quercetin (8-Br) were used as starting materials.

| Starting material | Amine | CuI [eq] | t [h] | T [°C] | Solvent | Aminoacid | Method |
|---|---|---|---|---|---|---|---|
| 8-I | glycine methyl ester | 0.1 | 16 | 100 | DMF | x | A |
| 8-I | *n*-hexylamine | 0.1 | 16 | 100 | DMF | x | A |
| 8-Br | x | 0.1 | 16 | 90 | DMF | alanine | A |
| 8-Br | x | 0.3 | 16 | 90 | DMSO | proline | A |
| 8-I* | *n*-hexylamine | 0.1 | 2 | 100 | DMF | x | A |
| 8-Br** | *n*-hexylamine | 0.3 | 16 | 90 | DMSO | proline | B |
| 8-Br | *n*-hexylamine | 0.3 | 16 | 90 | DMSO | alanine | B |
| 8-Br | *n*-hexylamine | 0.3 | 16 | 90 | DMSO | glycine | B |
| 8-Br | morpholine | 0.2 | 16 | 90 | DMSO | proline | B |
| 8-Br | morpholine | 0.2 | 16 | 90 | DMSO | *N*,*N*-dimethylglycine | B |

**Methods**: [A] 1.2 equiv of amine, CuI, 2.5 equiv of $K_2CO_3$

[B] 1.2 equiv amine, 0.25 equiv amino acid, CuI, 2.5 equiv of $K_2CO_3$

* reaction was carried out under microwave irradiation

** a product substituted with proline was observed

## 2. BIOLOGICAL ACTIVITY

**Table S2.** Cellular antioxidant activity (CAA) activity of quercetin, luteolin, and their derivatives.

| | $IC_{50}$ [µM] | *t*-test |
|---|---|---|
| Quercetin (**4**) | 5.9 ± 0.4 | |
| 8-(4-Fluoroanilino) quercetin (**5**) | 3.4 ± 0.2 | ** |
| 8-(4-Methoxyanilino) quercetin (**6**) | 7.5 ± 0.7 | |
| 8-(Anilino) quercetin (**7**) | 5.5 ± 0.4 | |
| 8-(4-(Trifluoromethyl)anilino) quercetin (**8**) | 11.5 ± 0.3 | °°° |
| 8-(3,5-Dimethoxyanilino) quercetin (**9**) | 5.7 ± 1.0 | |
| Luteolin (**10**) | 4.5 ± 0.5 | |
| 8-(4-Fluoroanilino) luteolin (**11**) | 2.9 ± 0.2 | * |
| 8-(4-Methoxyanilino) luteolin (**12**) | 3.4 ± 0.1 | |
| 8-(Anilino) luteolin (**13**) | 3.1 ± 0.1 | * |
| 8-(4-(Trifluoromethyl)anilino) luteolin (**14**) | > 12.5 | °°° |
| 8-(3-Fluoroanilino) luteolin (**15**) | 6.7 ±0.2 | °° |

Data are presented as the concentration (µM) that halved the antioxidant activity [$IC_{50}$]; average of four repetitions ± standard error of the mean. Stars indicate the statistically improved activity when compared to the parent compound (Student´s *t*-test, *p<0.05, ** p<0.005, ***p<0.005). Rings indicate the statistically decreased activity when compared to the parent compound (Student´s *t*-test, °p<0.05, °° p<0.005, °°°p<0.005).

**Table S3.** Anti-inflammatory activity of quercetin, luteolin, and their derivatives.

| | NO production | | IL-6 production | | TNFα production | |
|---|---|---|---|---|---|---|
| | $IC_{50}$ [µM] | *t*-test | $IC_{50}$ [µM] | *t*-test | $IC_{50}$ [µM] | *t*-test |
| Quercetin (**4**) | 9.3 ± 0.6 | | 4.7 ± 0.2 | | 55.1 ± 2.1 | |
| 8-(4-Fluoroanilino) quercetin (**5**) | 37.4 ± 1.6 | °°° | 14.6 ± 0.8 | °°° | > 50 | °°° |
| 8-(4-Methoxyanilino) quercetin (**6**) | 52.3 ± 2.3 | °°° | 32.3 ± 2.3 | °°° | > 50 | °°° |
| 8-(Anilino) quercetin (**7**) | 34.4 ± 0.2 | °°° | 12.9 ± 0.2 | °°° | > 100 | °°° |
| 8-(4-(Trifluoromethyl)anilino) quercetin (**8**) | 53.3 ± 0.8 | °°° | 35.1 ± 1.6 | °°° | > 50 | °°° |
| 8-(3,5-Dimethoxyanilino) quercetin (**9**) | 26.6 ± 0.4 | °°° | > 50 | °°° | > 50 | °°° |
| Luteolin (**10**) | 13.3 ± 0.2 | | 7.3 ± 0.2 | | 25.9 ± 0.3 | |
| 8-(4-Fluoroanilino) luteolin (**11**) | > 25 | °°° | 7.2 ± 0.3 | | 28.4 ± 0.2 | °°° |
| 8-(4-Methoxyanilino) luteolin (**12**) | 48.3 ± 1.3 | °°° | 15.3 ± 0.5 | °°° | > 50 | °°° |
| 8-(Anilino) luteolin (**13**) | 29.3 ± 0.1 | °°° | 7.3 ± 0.6 | | > 25 | °°° |
| 8-(4-(Trifluoromethyl)anilino) luteolin (**14**) | > 25 | °°° | 12.4 ± 0.4 | °°° | > 25 | °°° |
| 8-(3-Fluoroanilino) luteolin (**15**) | 31.8 ±0.9 | °°° | 8.0 ± 0.6 | | > 25 | °°° |

Data are presented as the concentration (µM) that halved the anti-inflammatory activity [$IC_{50}$]; average of four repetitions ± standard error of the mean. Stars indicate the statistically improved activity when compared to the parent compound (Student´s *t*-test, *p<0.05, ** p<0.005, ***p<0.005). Rings indicate the statistically decreased activity when compared to the parent compound (Student´s *t*-test, °p<0.05, °° p<0.005, °°°p<0.005).

**Table S4.** Cytotoxicity of the prepared derivatives is measured as the ability of flavonoids to halve the viability of immortalized human keratinocytes (HaCaT) and human dermal fibroblasts (HDF).

| | HaCaT | | HDF | |
|---|---|---|---|---|
| | $IC_{50}$ [µM] | *t*-test | $IC_{50}$ [µM] | *t*-test |
| Quercetin (**4**) | 126.7 ± 3.4 | | 80.0 ± 1.5 | |
| 8-(4-Fluoroanilino) quercetin (**5**) | 37.6 ± 3.4 | °°° | 95.9 ± 6.1 | |
| 8-(4-Methoxyanilino) quercetin (**6**) | 45.2 ± 3.5 | °°° | 79.9 ± 1.5 | |
| 8-(Anilino) quercetin (**7**) | 102.5 ± 1.5 | °°° | 114.8 ± 1.2 | *** |
| 8-(4-(Trifluoromethyl) anilino) quercetin (**8**) | 117.1 ± 0.1 | °° | 111.9 ± 0.5 | *** |
| 8-(3,5-Dimethoxyanilino) quercetin (**9**) | 56.9 ± 1.9 | °°° | 76.0 ± 3.0 | |
| Luteolin (**10**) | 34.7 ± 1.0 | | 42.9 ± 3.4 | |
| 8-(4-Fluoroanilino) luteolin (**11**) | 76.9 ± 7.7 | ** | 51.9 ± 3.7 | |
| 8-(4-Methoxyanilino) luteolin (**12**) | 59.3 ± 6.3 | * | 35.5 ± 1.5 | |
| 8-(Anilino) luteolin (**13**) | 41.4 ± 3.7 | | 58.3 ± 2.7 | * |
| 8-(4-(Trifluoromethyl)anilino) luteolin (**14**) | 26.8 ± 1.6 | ° | 29.9 ± 1.1 | ° |
| 8-(3-Fluoroanilino) luteolin (**15**) | 26.0 ± 5.8 | | 30.9 ± 2.1 | ° |
| **Doxorubicin** | **0.42 ± 0.03** | | **0.19 ± 0.01** | |

Data are presented as the concentration (µM) that halved the viability [$IC_{50}$]; average of three repetitions ± standard error of the mean. Stars indicate the statistically improved activity when compared to the parent compound (Student´s *t*-test, *p<0.05, ** p<0.005, ***p<0.005). Rings indicate the statistically decreased activity when compared to the parent compound (Student´s *t*-test, °p<0.05, °° p<0.005, °°°p<0.005).

**Table S5.** Antibacterial activity of quercetin (**4**), luteolin (**10**), and their derivatives against antibiotic-sensitive (CCM 4223) and multidrug-resistant (MRSA 9) *S. aureus*. The derivatives produced showed no antibacterial activity against *P. aeruginosa* at concentrations below 200 µM.

| | *S. aureus* CCM 4223 | | *S. aureus* MRSA 9 | |
|---|---|---|---|---|
| | $IC_{50}$ [µM] | *t*-test | $IC_{50}$ [µM] | *t*-test |
| Quercetin (**4**) | > 200 | | > 200 | |
| 8-(4-Fluoroanilino) quercetin (**5**) | 28.0 ± 0.1 | *** | 60.7 ± 2.4 | *** |
| 8-(4-Methoxyanilino) quercetin (**6**) | 44.0 ± 0.1 | *** | 138.0 ± 1.2 | *** |
| 8-(Anilino) quercetin (**7**) | 32.7 ± 0.7 | *** | 70.0 ± 2.0 | *** |
| 8-(4-(Trifluoromethyl)anilino) quercetin (**8**) | 39.3 ± 1.8 | *** | 35.3 ± 0.7 | *** |
| 8-(3,5-Dimethoxyanilino) quercetin (**9**) | > 200 | | > 200 | |
| Luteolin (**10**) | > 200 | | > 200 | |
| 8-(4-Fluoroanilino) luteolin (**11**) | 42.0 ± 1.2 | *** | 38.0 ± 1.2 | *** |
| 8-(4-Methoxyanilino) luteolin (**12**) | 54.0 ± 0.1 | *** | 112.0 ± 1.2 | *** |
| 8-(Anilino) luteolin (**13**) | 50.0 ± 1.2 | *** | 51.3 ± 1.3 | *** |
| 8-(4-(Trifluoromethyl)anilino) luteolin (**14**) | 15.3 ± 0.7 | *** | 24.0 ± 0.1 | *** |
| 8-(3-Fluoroanilino) luteolin (**15**) | 28.0 ± 2.2 | *** | 56.9 ± 1.9 | *** |

Data are presented as the concentration (µM) that halved the antibacterial activity [$IC_{50}$]; average of three repetitions ± standard error of the mean. Stars indicate the statistically improved activity when compared to the parent compound (Student´s *t*-test, *p<0.05, ** p<0.005, ***p<0.0005).

**Table S6.** Mode of antimicrobial action of quercetin, luteolin and their derivatives with gentamicin against methicilin-resistant *Staphylococcus aureus* 3596 strain positive for the presence of the *aadD* gene encoding 4′,4″-adenyltransferase and the strain 1584 positive for the presence of the *aac(6')/aph(2')* gene encoding bifunctional aminoglycoside-modifying enzyme with acetylation and phosphotransferase activities.

| | **MRSA 3596** | | | | **MRSA 1584** | | | |
|---|---|---|---|---|---|---|---|---|
| | **MIC [µM]** | | **FIC** | **effect** | **MIC [µM]** | | **FIC** | **effect** |
| **Gentamicin [1 mg/L]** | **yes** | **no** | | | **yes** | **no** | | |
| **Quercetin** (**4**) | **54.5 ± 0.6** | **178.1 ± 2.7** | **0.31 ± 0.01** | **synergy** | **55.6 ± 1.1** | **88.6 ± 0.3** | **0.63 ± 0.01** | **additive** |
| 8-(4-Fluoroanilino) quercetin (**5**) | > 200 | > 200 | | | 28.2 ± 0.5 | 29.2 ± 0.1 | 0.97 ± 0.02 | additive |
| 8-(Anilino) quercetin (**7**) | > 200 | > 200 | | | 31.9 ± 2.5 | 29.9 ± 1.2 | 1.07 ± 0.13 | indifferent |
| 8-(4-(Trifluoromethyl)anilino) quercetin (**8**) | 5.7 ± 0.1 | 37.9 ± 0.1 | 0.15 ± 0.01 | synergy | 23.9 ± 0.1 | 24.5 ± 0.4 | 0.97 ± 0.02 | additive |
| **Luteolin** (**10**) | **6.7 ± 0.1** | **51.1 ± 1.3** | **0.13 ± 0.01** | **synergy** | **7.6 ± 1.0** | **15.2 ± 1.4** | **0.50 ± 0.11** | **additive** |
| 8-(4-Fluoroanilino) luteolin (**11**) | > 200 | > 200 | | | 15.7 ± 0.9 | 24.1 ± 0.7 | 0.65 ± 0.06 | additive |
| 8-(4-Methoxyanilino) luteolin (**12**) | 31.4 ± 1.0 | 91.0 ± 0.9 | 0.34 ± 0.01 | synergy | 47.1 ± 0.8 | 72.9 ± 2.4 | 0.65 ± 0.03 | additive |
| 8-(Anilino) luteolin (**13**) | 23.2 ± 0.4 | 93.0 ± 0.6 | 0.25 ± 0.01 | synergy | 49.9 ± 8.1 | 70.9 ± 0.3 | 0.70 ± 0.12 | additive |
| 8-(4-(Trifluoromethyl)anilino) luteolin (**14**) | > 200 | > 200 | | | 26.1 ± 1.6 | 15.3 ± 1.3 | 1.71 ± 0.26 | indifferent |
| 8-(3-Fluoroaniline) luteolin (**15**) | 5.3 ± 0.1 | 25.7 ± 0.4 | 0.21 ± 0.1 | synergy | 27.6 ± 0.5 | 28.6 ± 0.1 | 0.96 ± 0.02 | additive |

Fractional Inhibitory Concentration Index (FIC) < 0.5 indicates synergism, > 0.5-1 indicates additive effects, > 1 to < 2 indicates indifference, and ≥ 2 indicates antagonism.

**Table S7**. Docking of *S*-adenosyl-methionine, quercetin (**4**), luteolin (**10**), and their selected derivatives into the model of ribosomal methyltransferase (encoded by the *ermC* gene). The table illustrates the best free energy of binding score *ΔG* (kcal/mol) value.

| | *ΔG* [kcal/mol] |
|---|---|
| | SAM-pocket |
| *S*-Adenosyl-methionine | -4.534 |
| Quercetin (**4**) | -6.862 |
| Luteolin (**10**) | -6.639 |
| 8-(4-(Trifluoromethyl)anilino) quercetin (**8**) | -6.002 |
| 8-(4-Fluoroanilino) luteolin (**11**) | -4.897 |
| 8-(4-(Trifluoromethyl)anilino) luteolin (**14**) | -4.108 |

"/" means that there isn't any binding pose in that pocket.

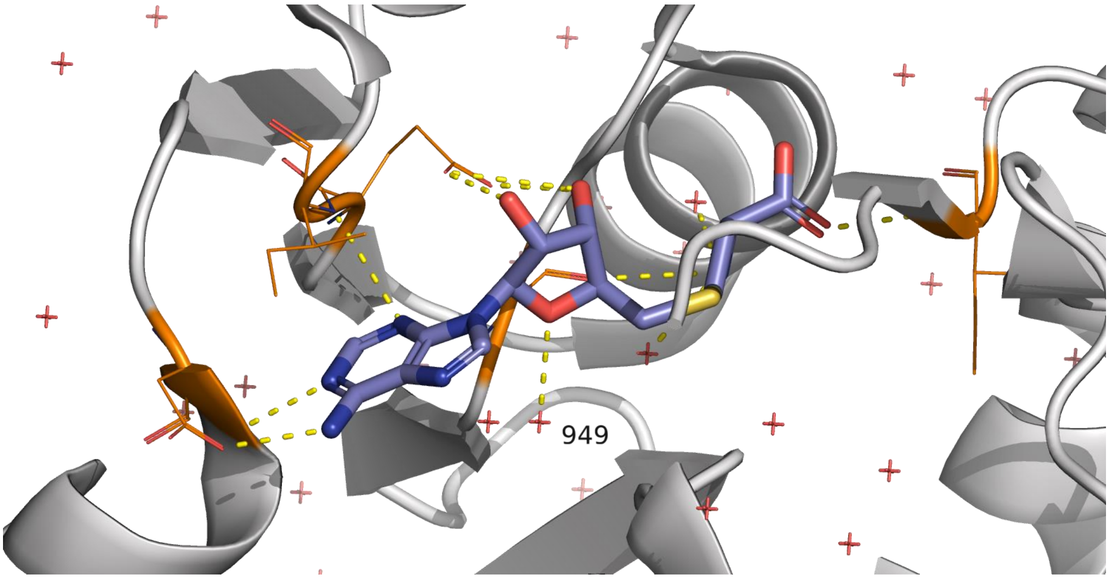


**Figure S1.** The observed differences in SAM orientation are attributed to the presence of water HOH-949.

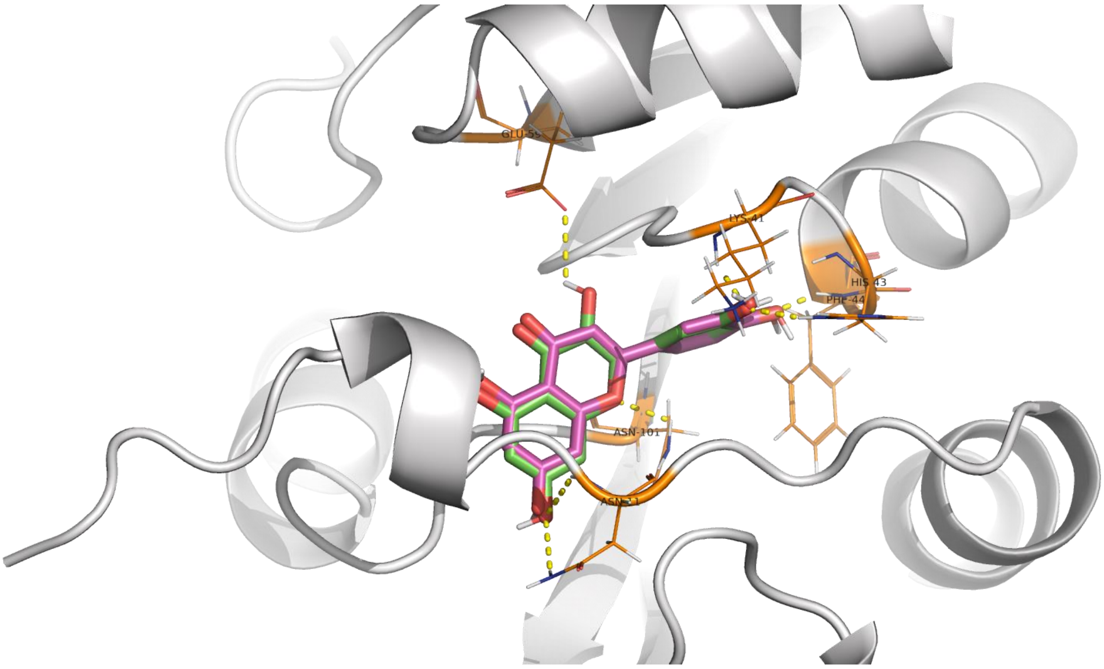


**Figure S2.** Docking of quercetin (**4**) and luteolin (**10**) into methyltrasferase.

## 3. NMR SPECTRA

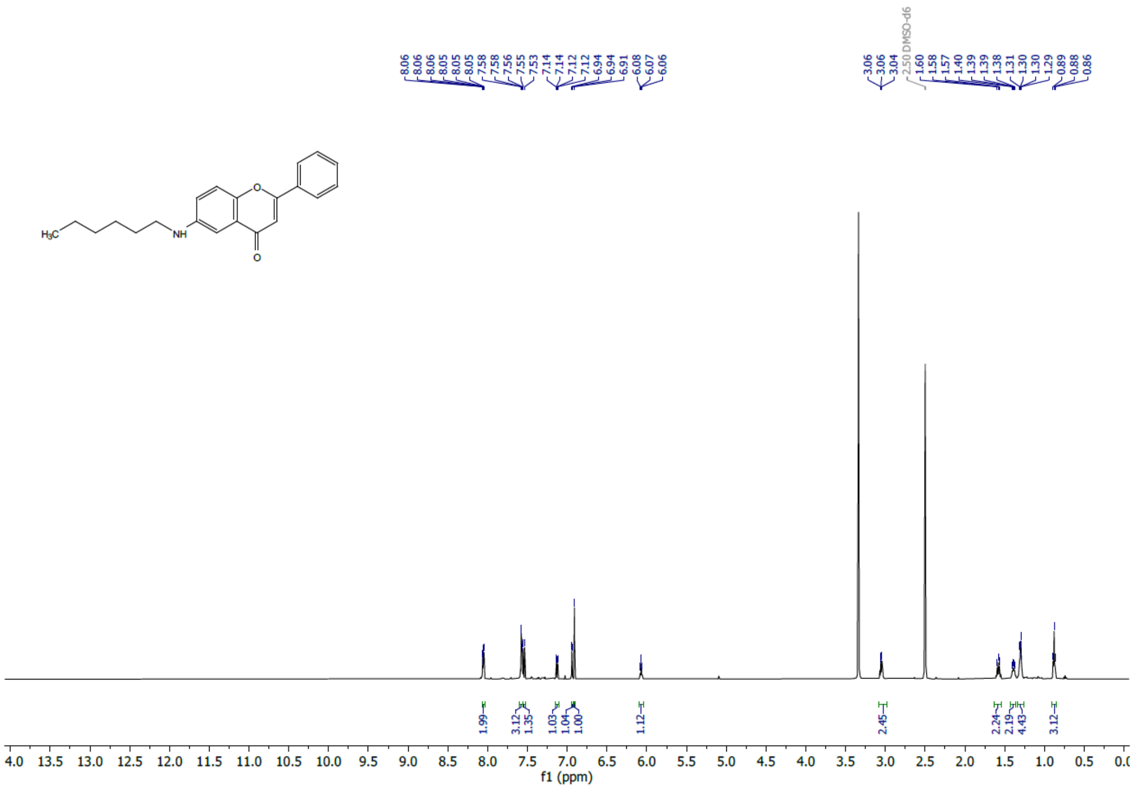

**Figure S3.** $^{1}H$ NMR (500 MHz, DMSO-$d_6$, 25 °C) spectrum of 6-hexylamino flavone (**2**).

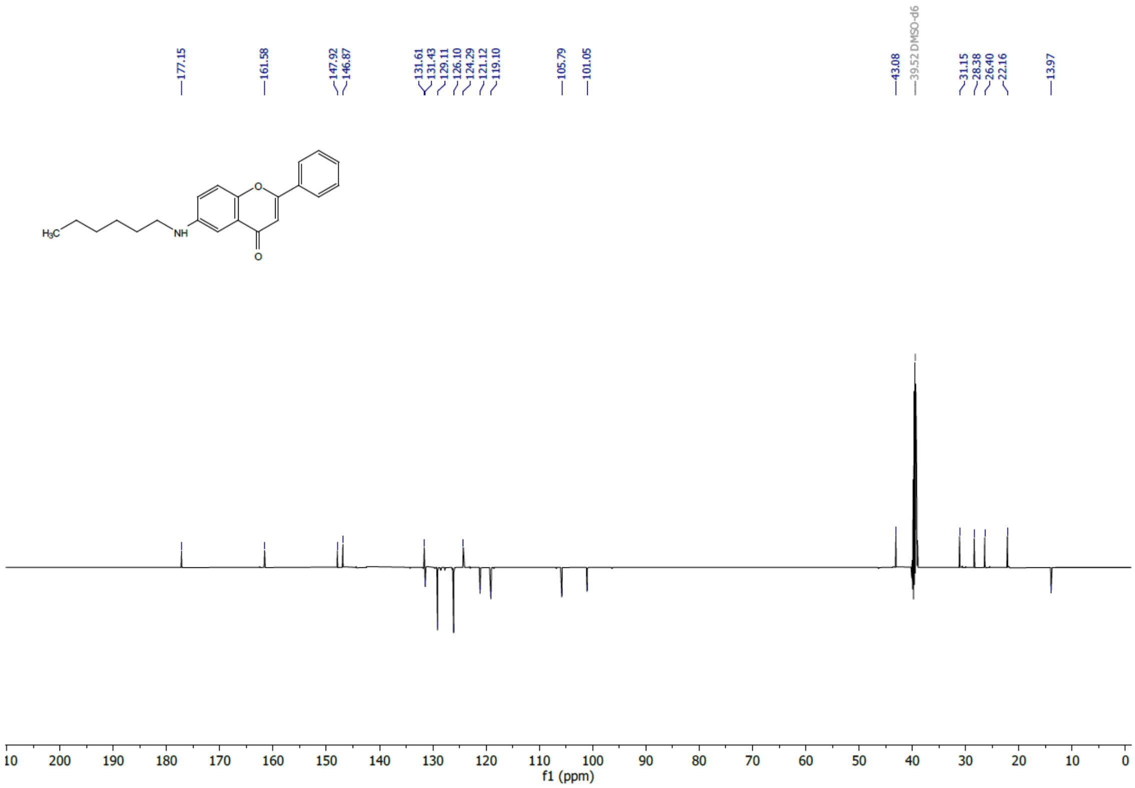

**Figure S4.** $^{13}C\{^{1}H\}$ APT NMR (126 MHz, DMSO-$d_6$, 25 °C) spectrum of 6-hexylamino flavone (**2**).

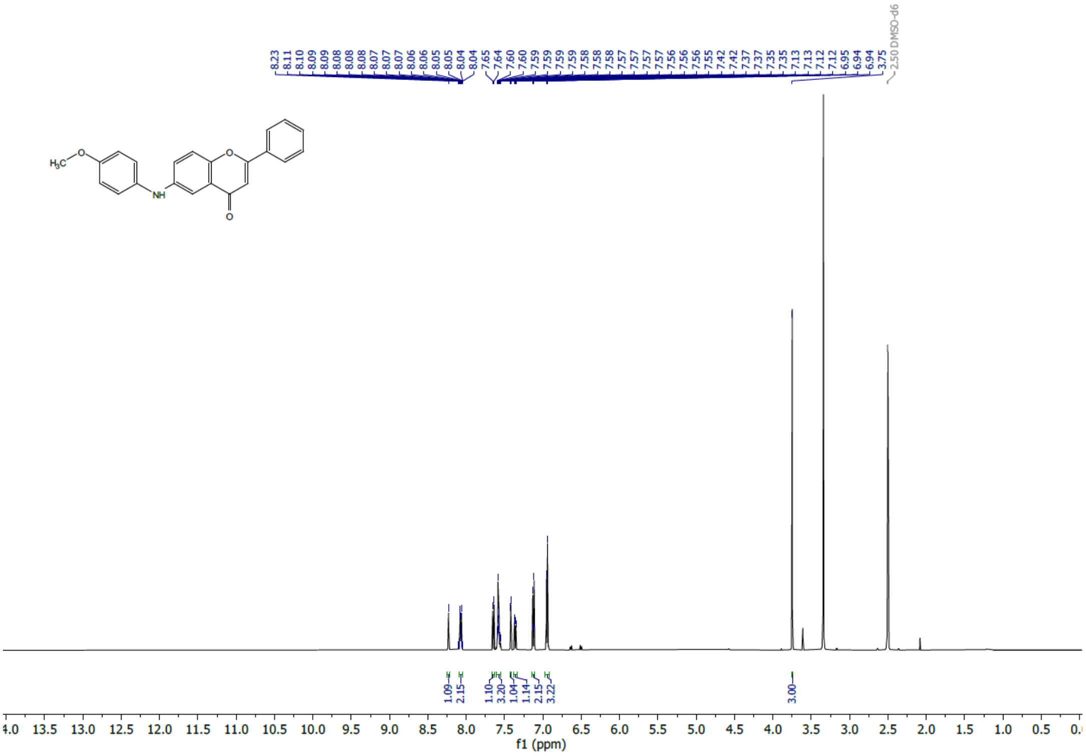

**Figure S5.** $^{1}H$ NMR (500 MHz, DMSO-$d_6$, 25 °C) spectrum of 6-(4-methoxyanilino) flavone (**3**).

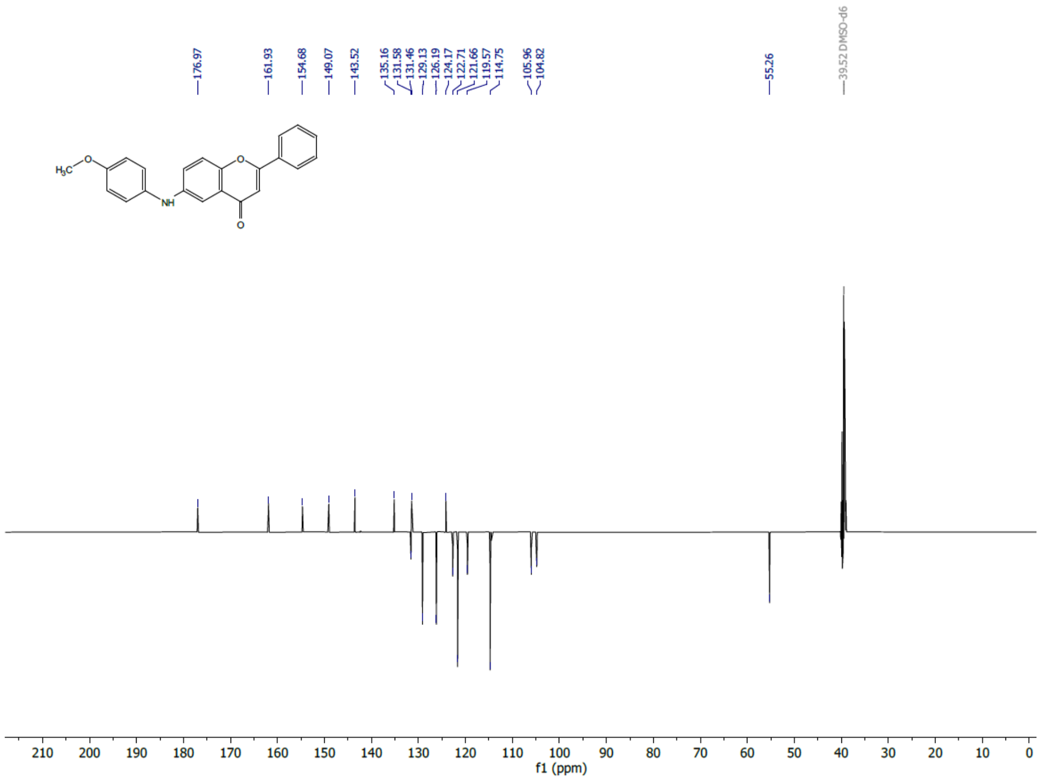

**Figure S6.** $^{13}C\{^{1}H\}$ APT NMR (126 MHz, DMSO-$d_6$, 25 °C) spectrum of 6-(4-methoxyanilino) flavone (**3**).

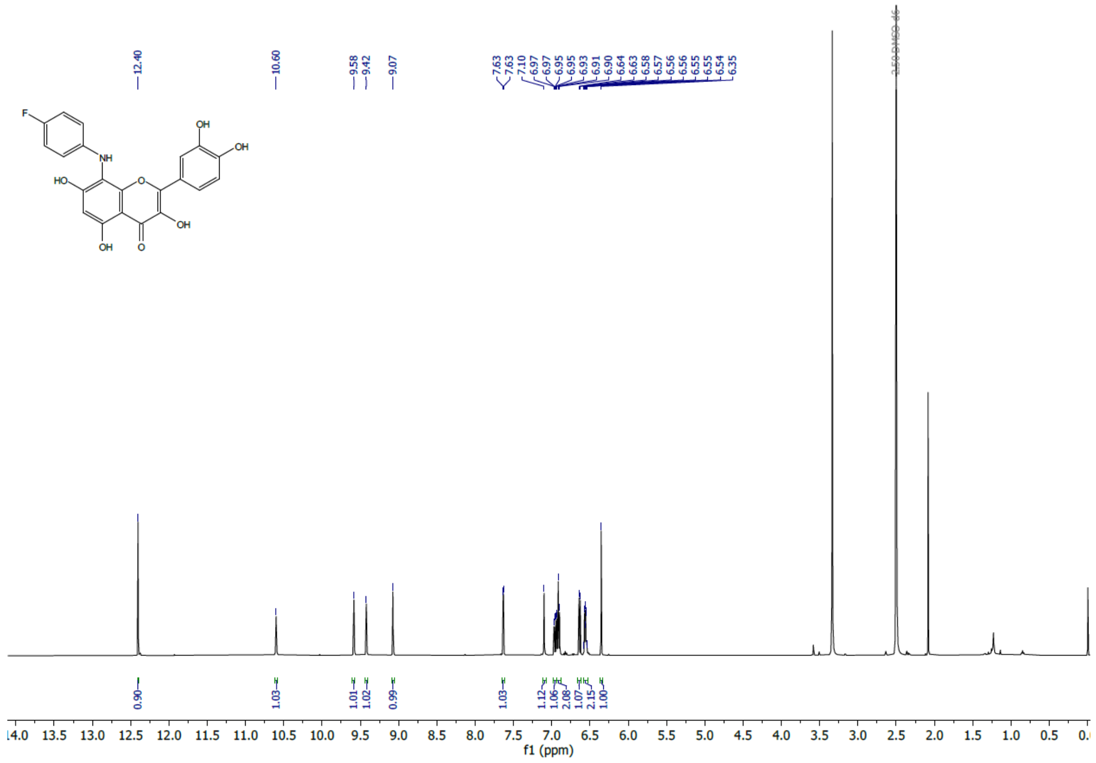

**Figure S7.** $^{1}$H NMR (500 MHz, DMSO-$d_6$, 25 °C) spectrum of 8-(4-fluoroanilino) quercetin (**5**).

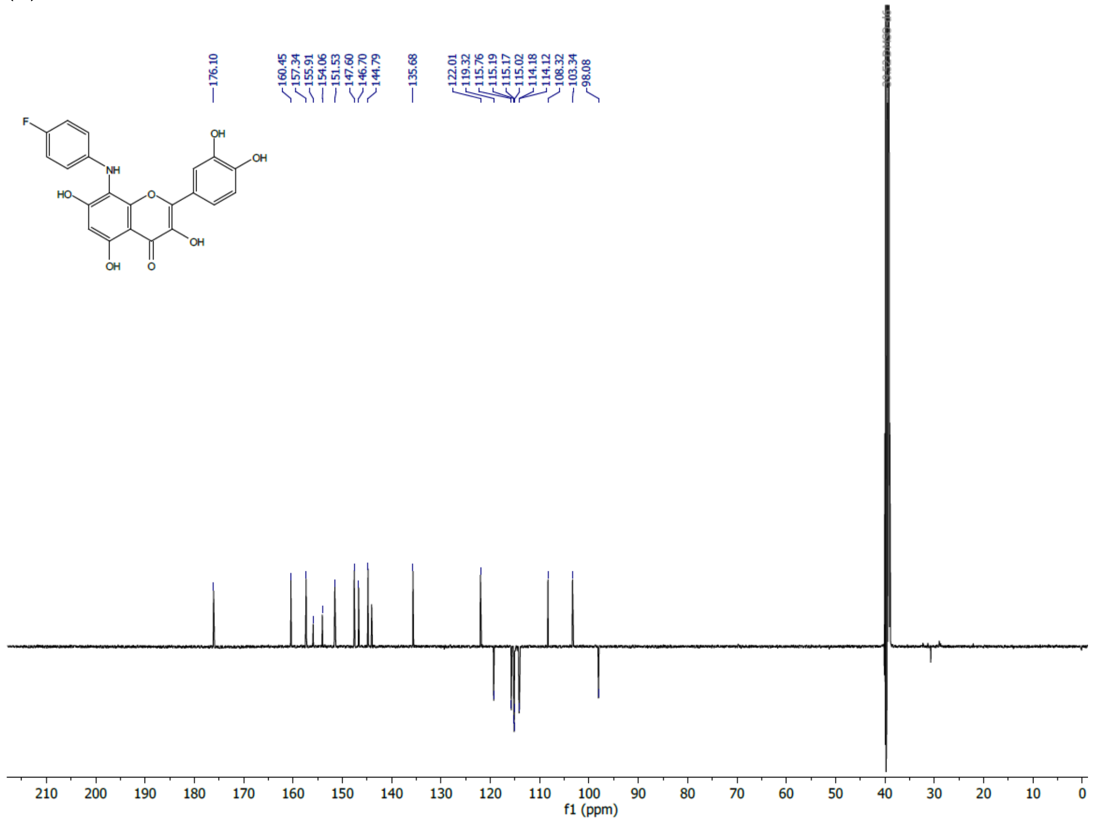

**Figure S8.** $^{13}$C{$^{1}$H} APT NMR (126 MHz, DMSO-$d_6$, 25 °C) spectrum of 8-(4-fluoroanilino) quercetin (**5**).

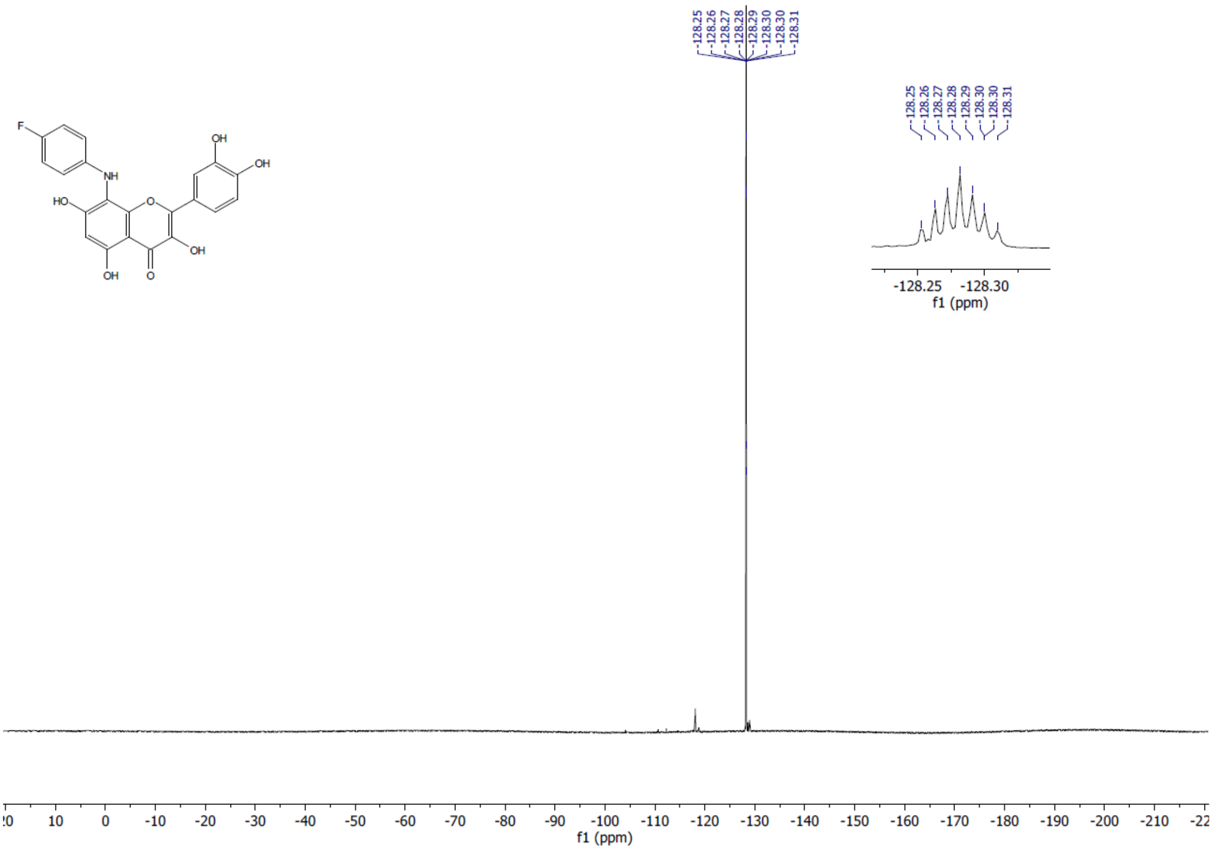


**Figure S9.** $^{19}$F NMR (470 MHz, DMSO-$d_6$, 25 °C) spectrum of 8-(4-fluoroanilino) quercetin (**5**).

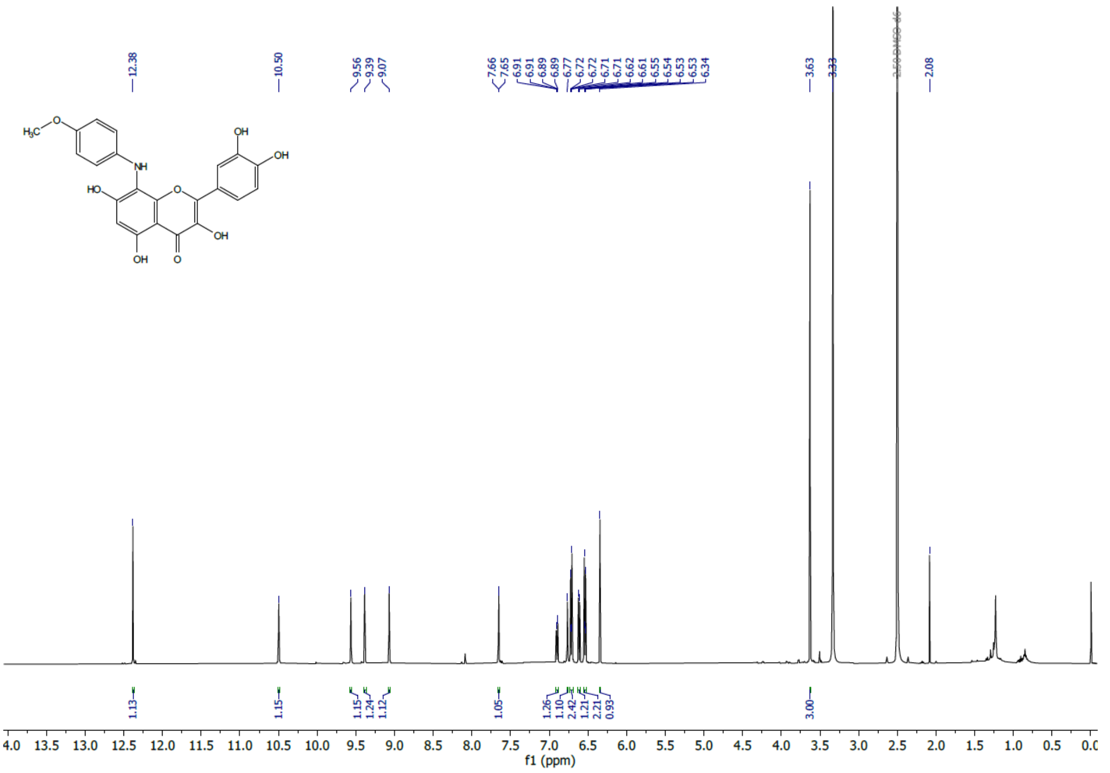

**Figure S10.** $^{1}$H NMR (500 MHz, DMSO-$d_6$, 25 °C) spectrum of 8-(4-methoxyanilino) quercetin (**6**).

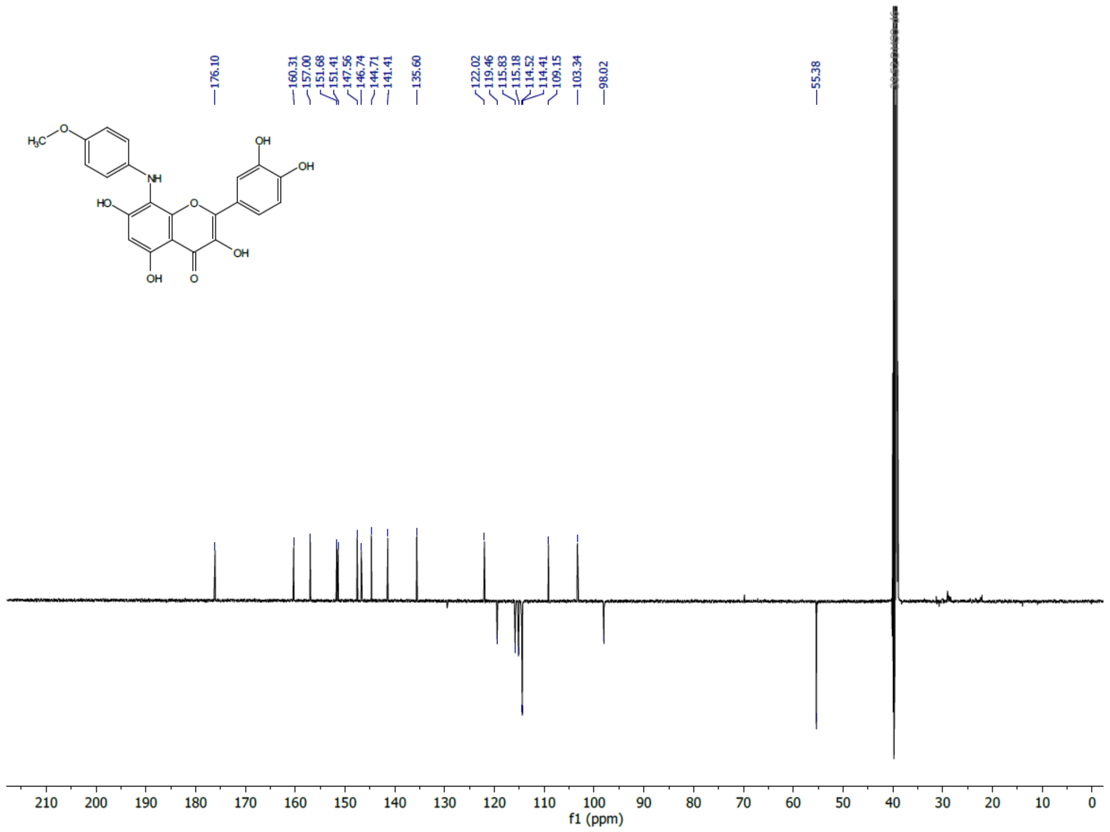

**Figure S11.** $^{13}$C{$^{1}$H} APT NMR (126 MHz, DMSO-$d_6$, 25 °C) spectrum of 8-(4-methoxyanilino) quercetin (**6**).

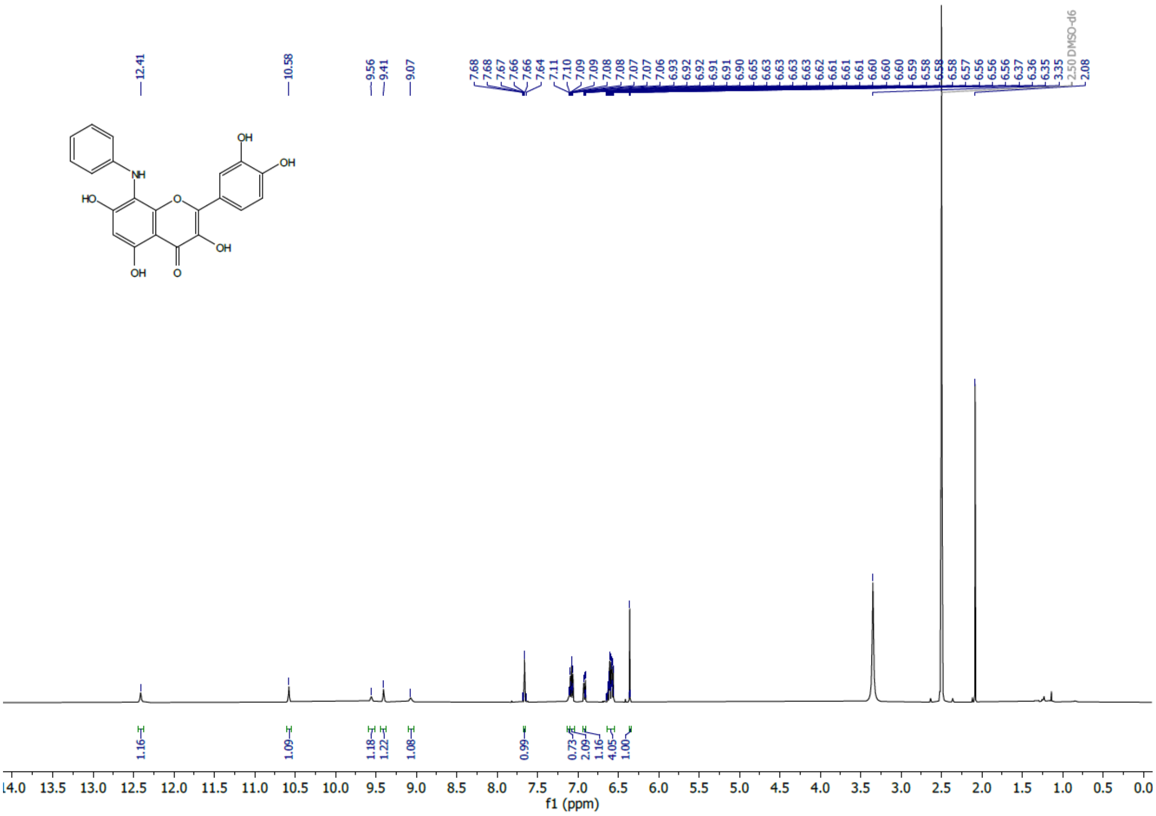

**Figure S12.** $^{1}H$ NMR (500 MHz, DMSO-$d_6$, 25 °C) spectrum of 8-(anilino) quercetin (**7**).

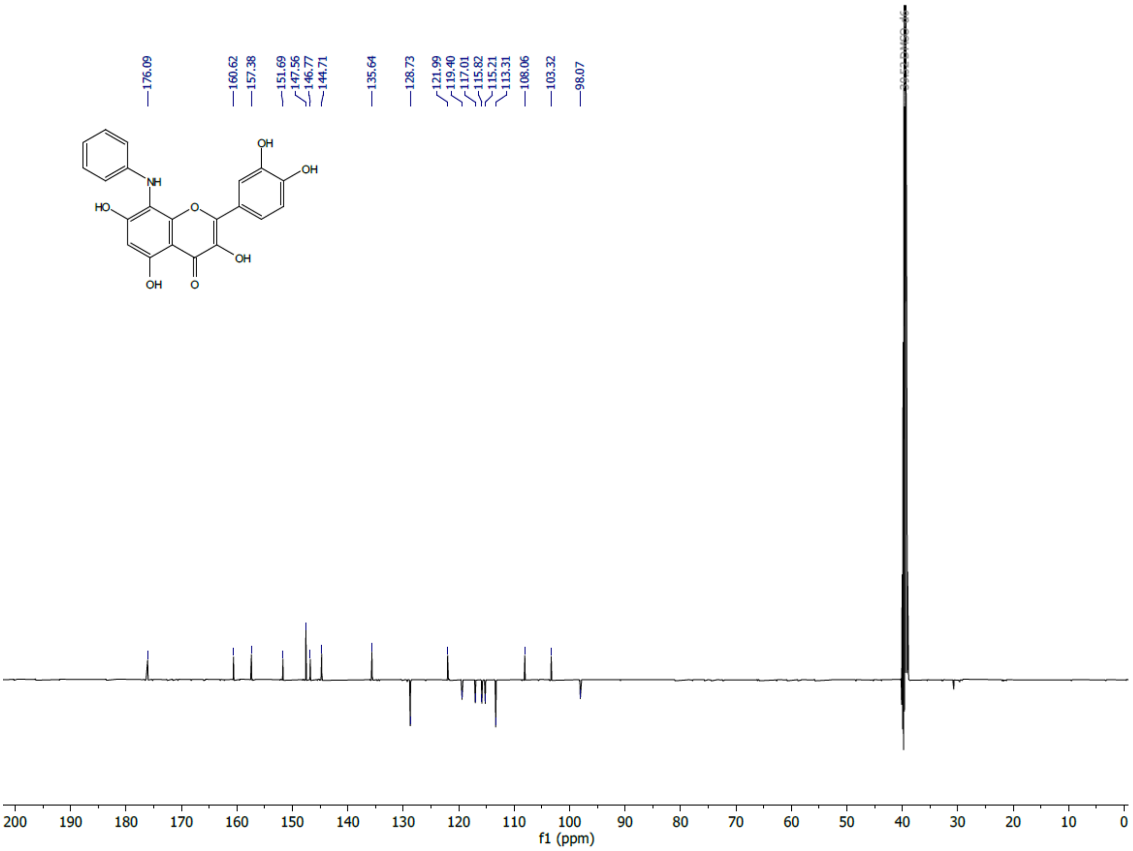

**Figure S13.** $^{13}C\{^{1}H\}$ APT NMR (126 MHz, DMSO-$d_6$, 25 °C) spectrum of 8-(anilino) quercetin (**7**).

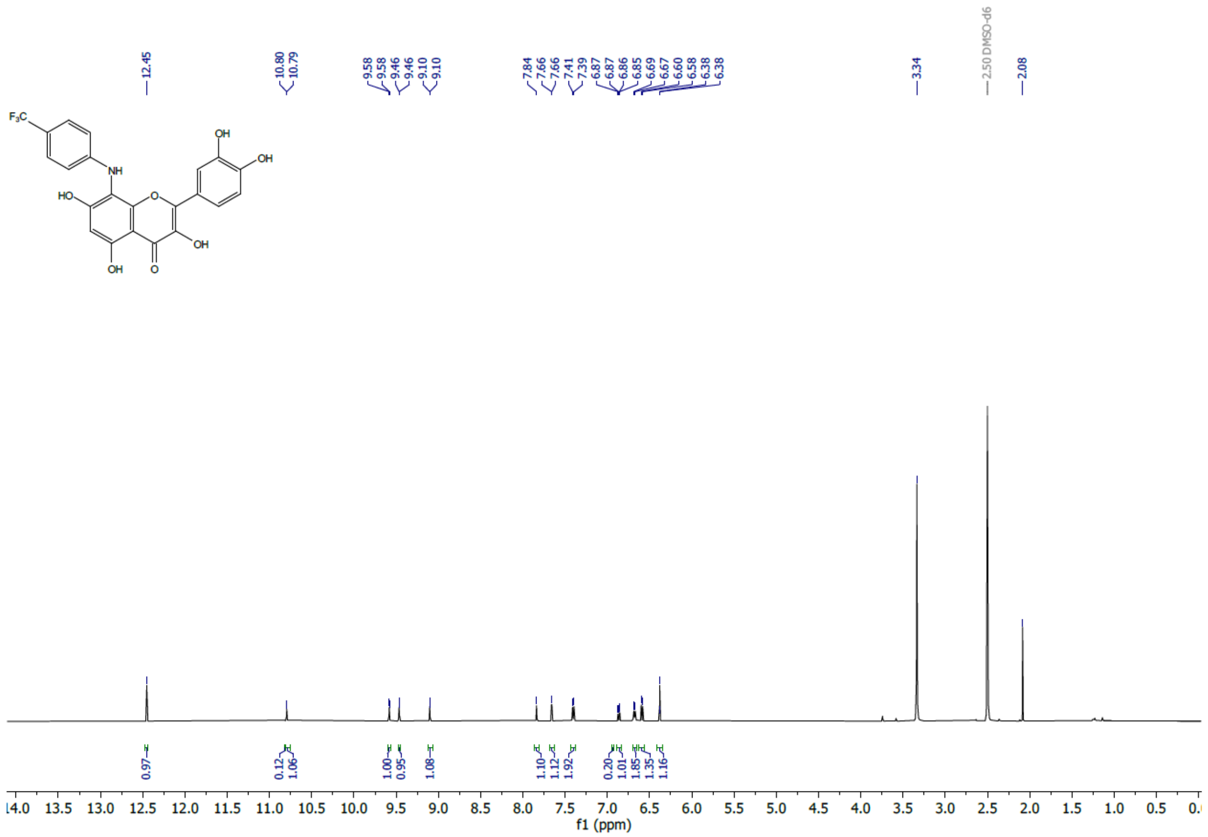

**Figure S14.** $^1$H NMR (500 MHz, DMSO-$d_6$, 25 °C) spectrum of 8-(4-(trifluoromethyl)anilino) quercetin (**8**).

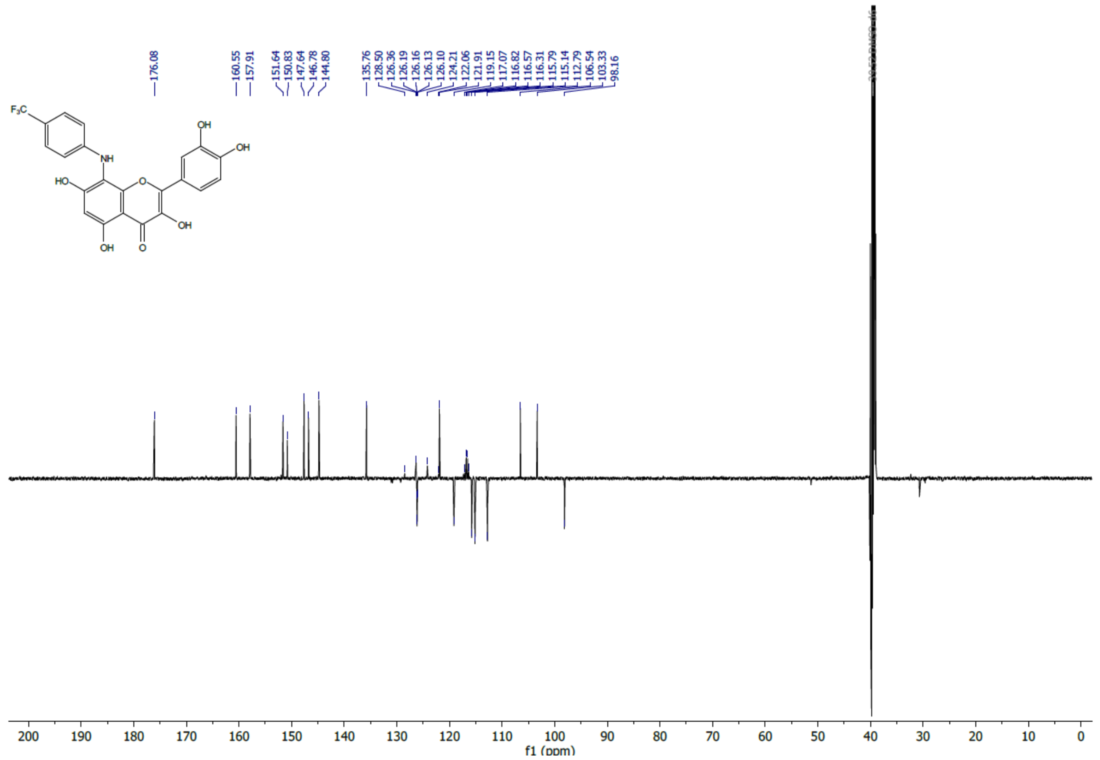

**Figure S15.** $^{13}$C{$^1$H} APT NMR (126 MHz, DMSO-$d_6$, 25 °C) spectrum of 8-(4-(trifluoromethyl)anilino) quercetin (**8**).

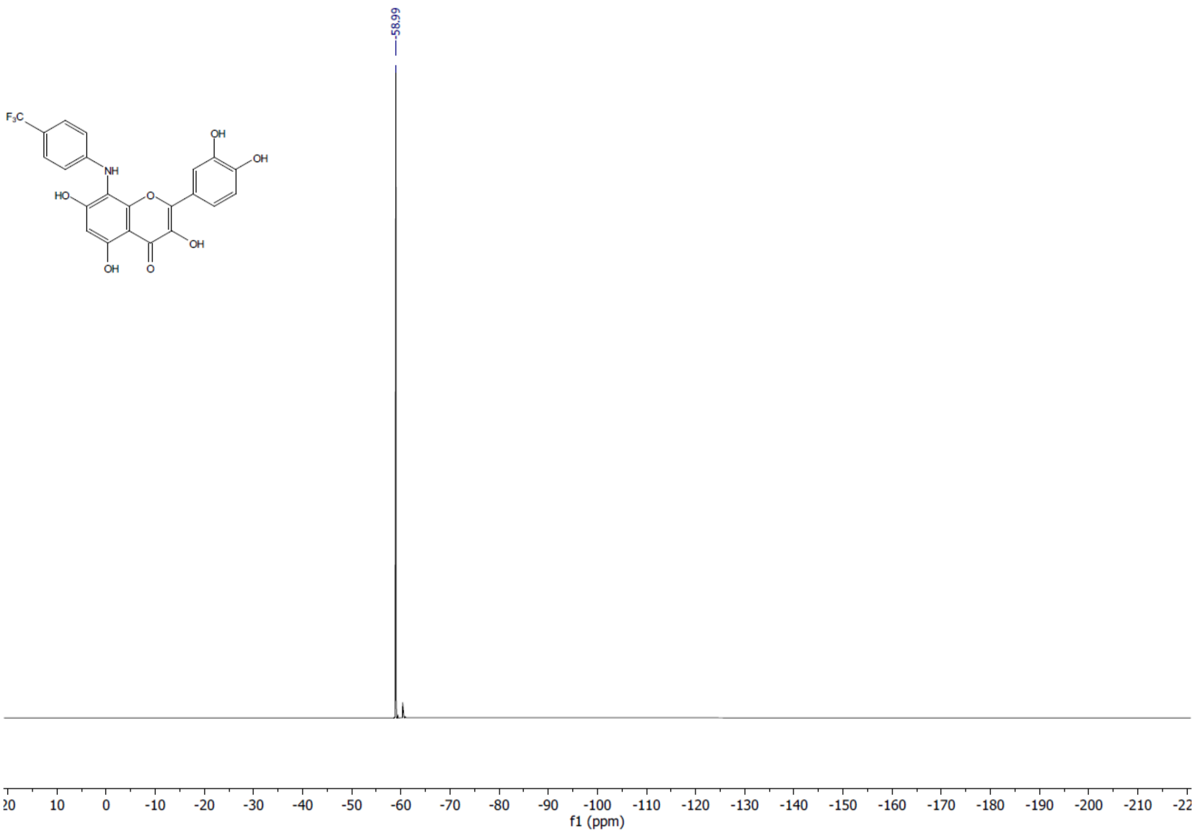


**Figure S16.** $^{19}$F NMR (470 MHz, DMSO-$d_6$, 25 °C) spectrum of 8-(4-(trifluoromethyl)anilino) quercetin (**8**).

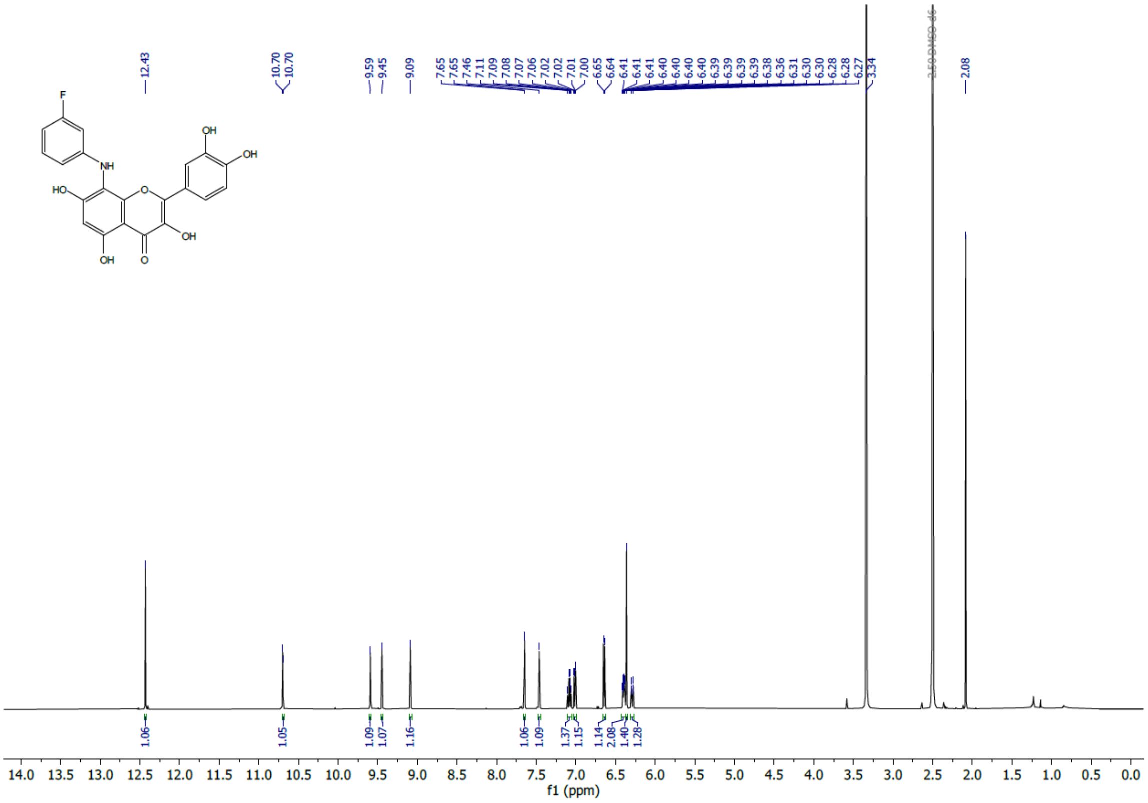

**Figure S17.** $^{1}H$ NMR (500 MHz, DMSO-$d_6$, 25 °C) spectrum of 8-(3-fluoroanilino) quercetin (**17**).

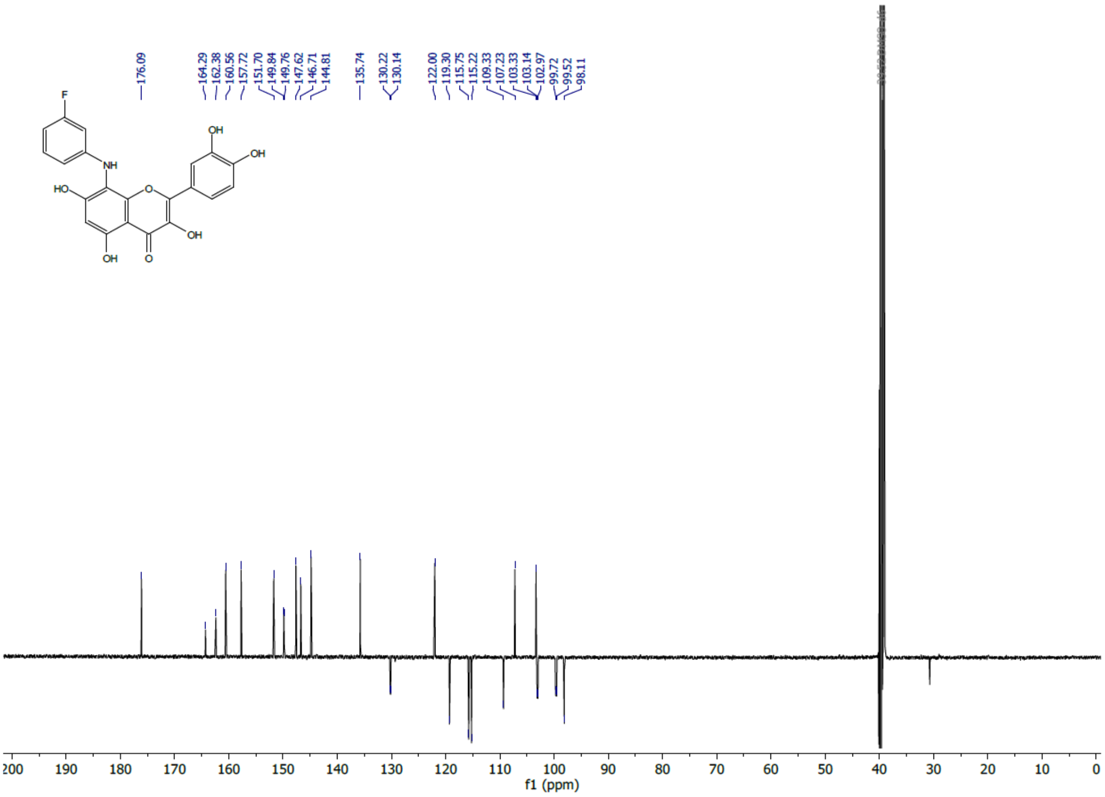

**Figure S18.** $^{13}C\{^{1}H\}$ APT NMR (126 MHz, DMSO-$d_6$, 25 °C) spectrum of 8-(3-fluoroanilino) quercetin (**17**).

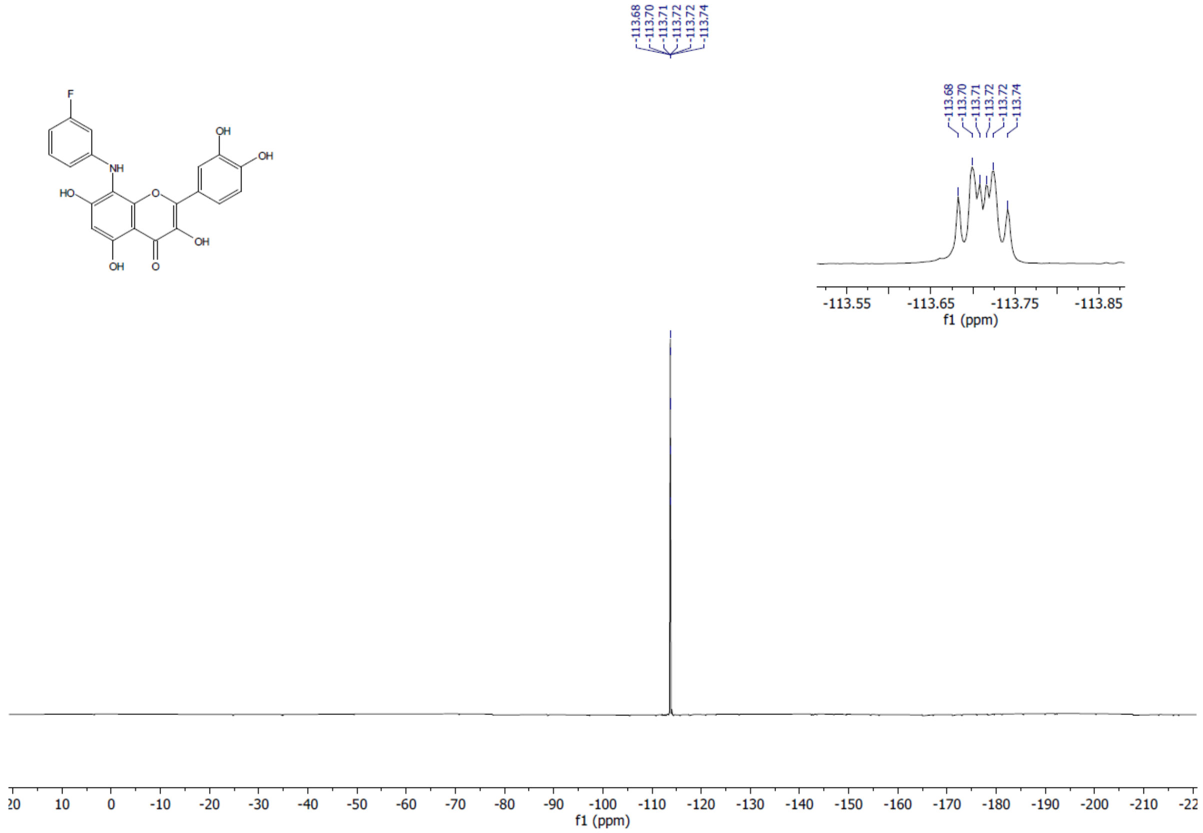


**Figure S19.** $^{19}$F NMR (470 MHz, DMSO-$d_6$, 25 °C) spectrum of 8-(3-fluoroanilino) quercetin (**17**).

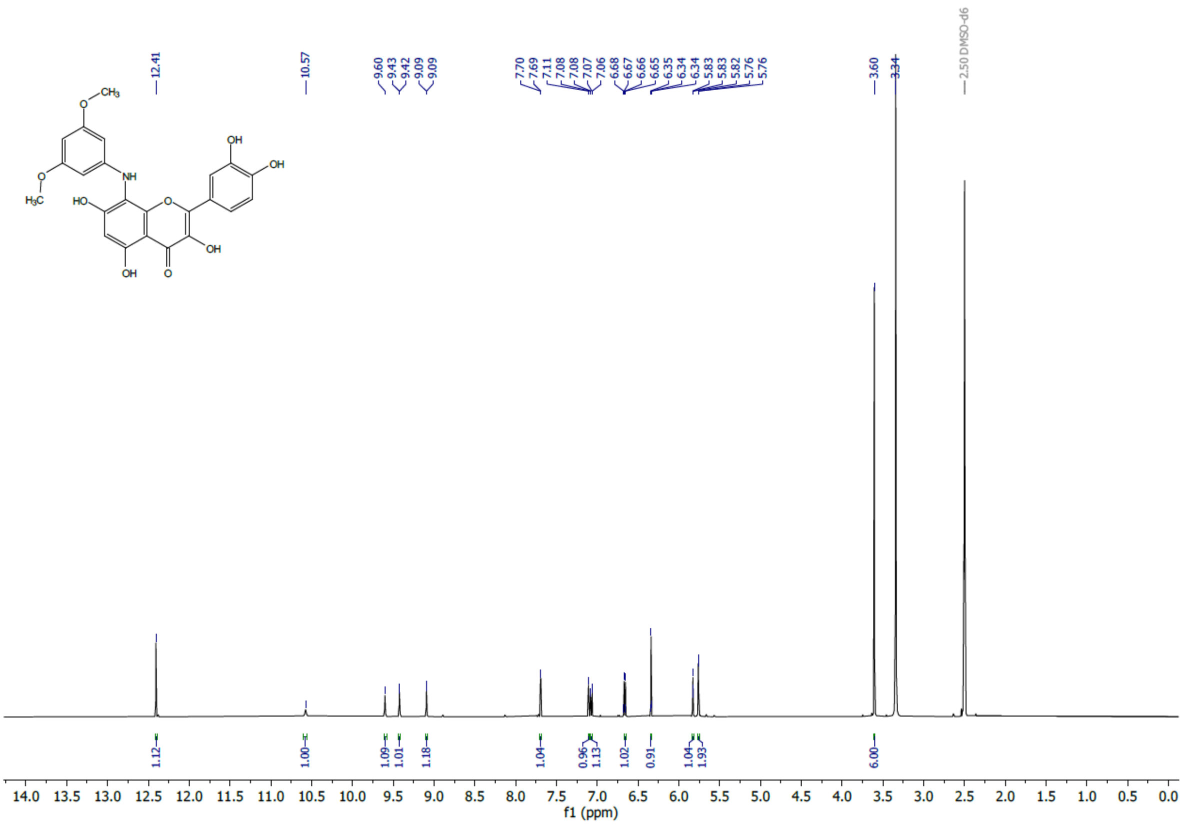

**Figure S20.** $^{1}H$ NMR (500 MHz, DMSO-$d_6$, 25 °C) spectrum of 8-(3,5-dimethoxyanilino) quercetin (**9**).

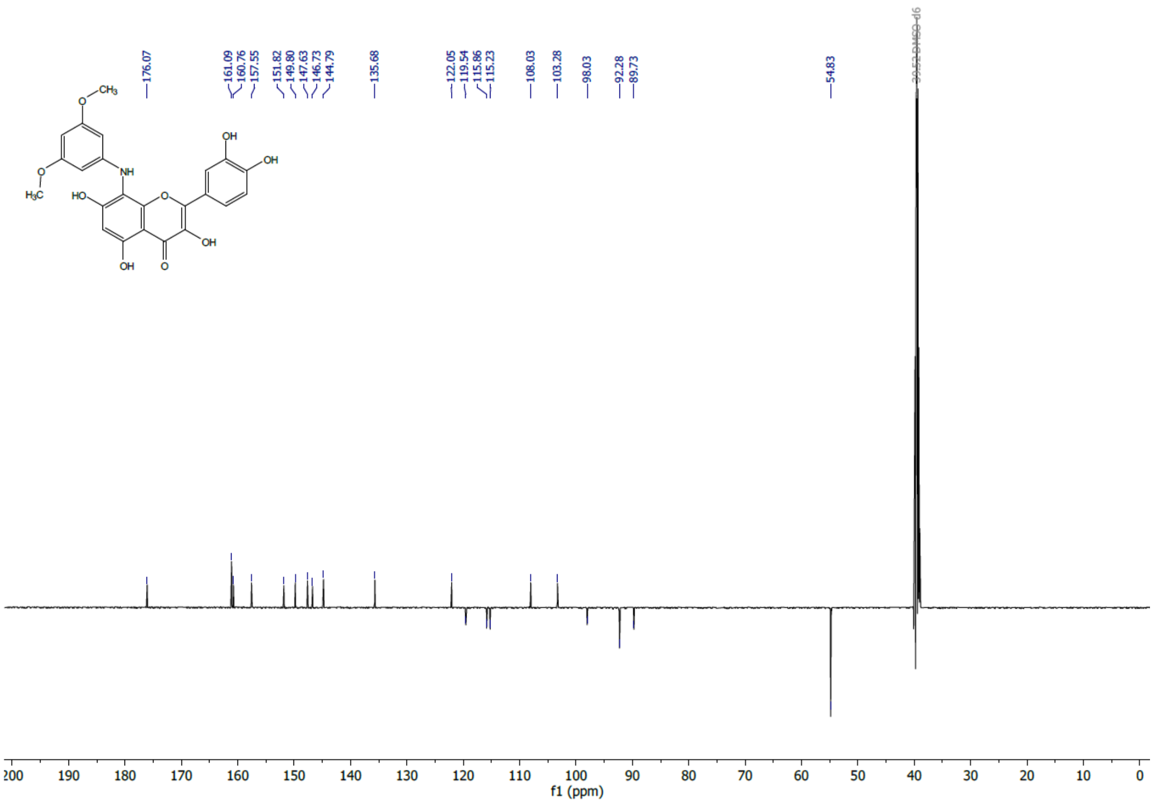

**Figure S21.** $^{13}C\{^{1}H\}$ APT NMR (126 MHz, DMSO-$d_6$, 25 °C) spectrum of 8-(3,5-dimethoxyanilino) quercetin (**9**).

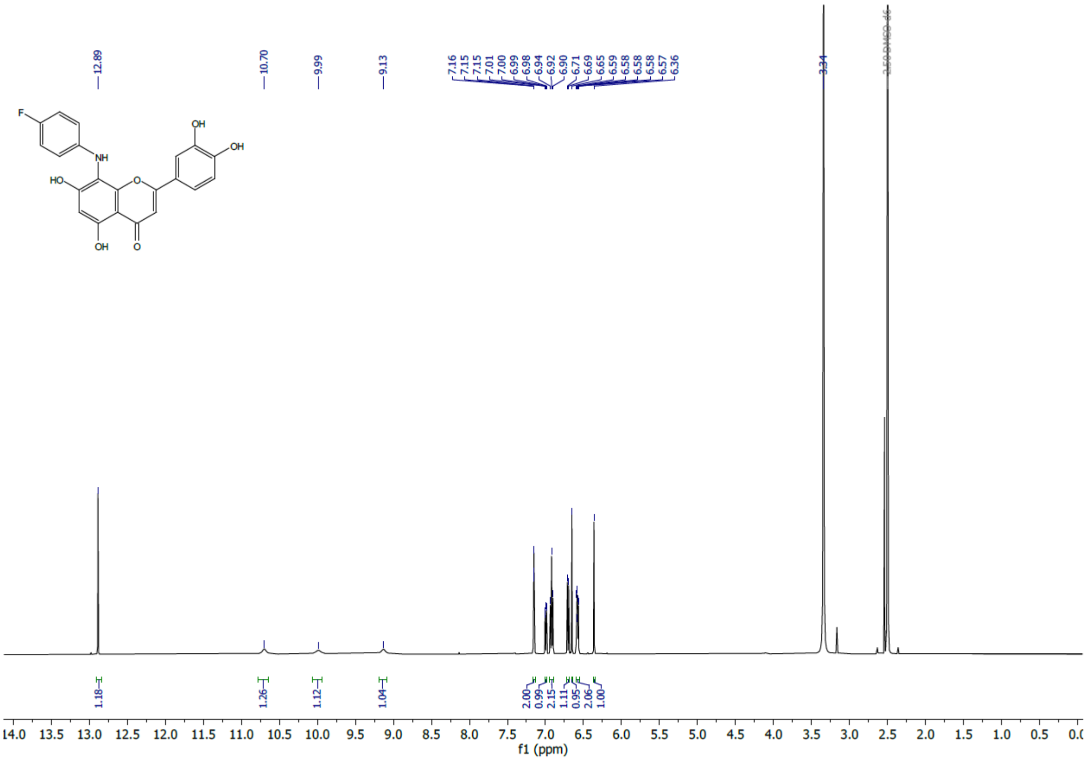


**Figure S22.** $^{1}$H NMR (500 MHz, DMSO-$d_6$, 25 °C) spectrum of 8-(4-fluoroanilino) luteolin (**11**).

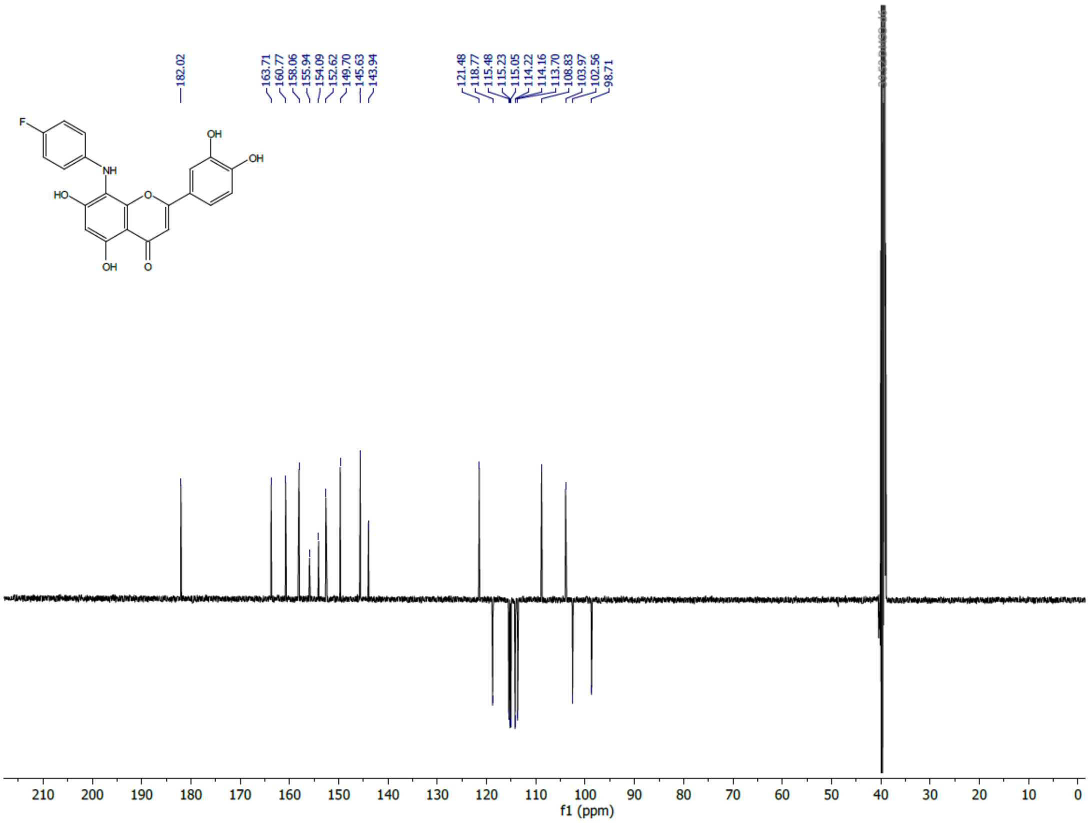


**Figure S23.** $^{13}$C{$^{1}$H} APT NMR (126 MHz, DMSO-$d_6$, 25 °C) spectrum of 8-(4-fluoroanilino) luteolin (**11**).

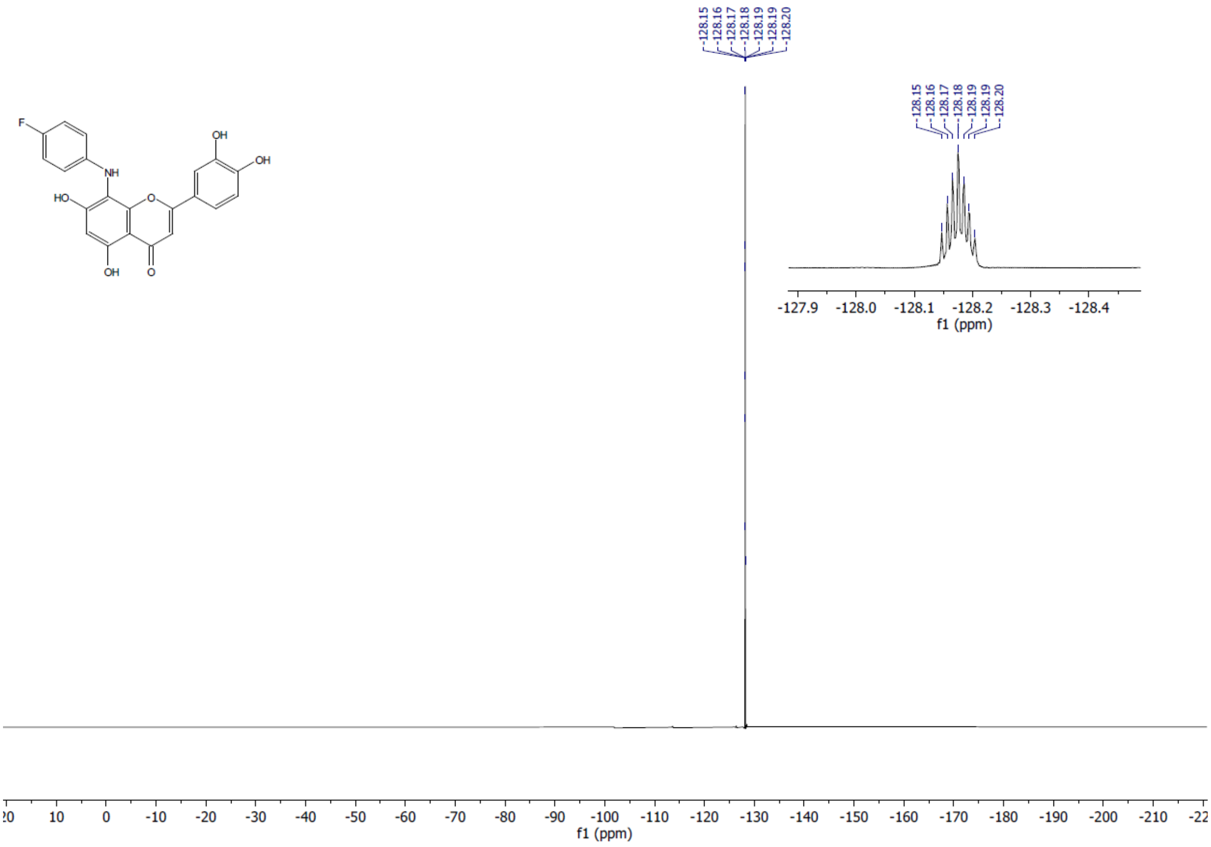


**Figure S24.** $^{19}$F NMR (470 MHz, DMSO-$d_6$, 25 °C) spectrum of 8-(4-fluoroanilino) luteolin (**11**).

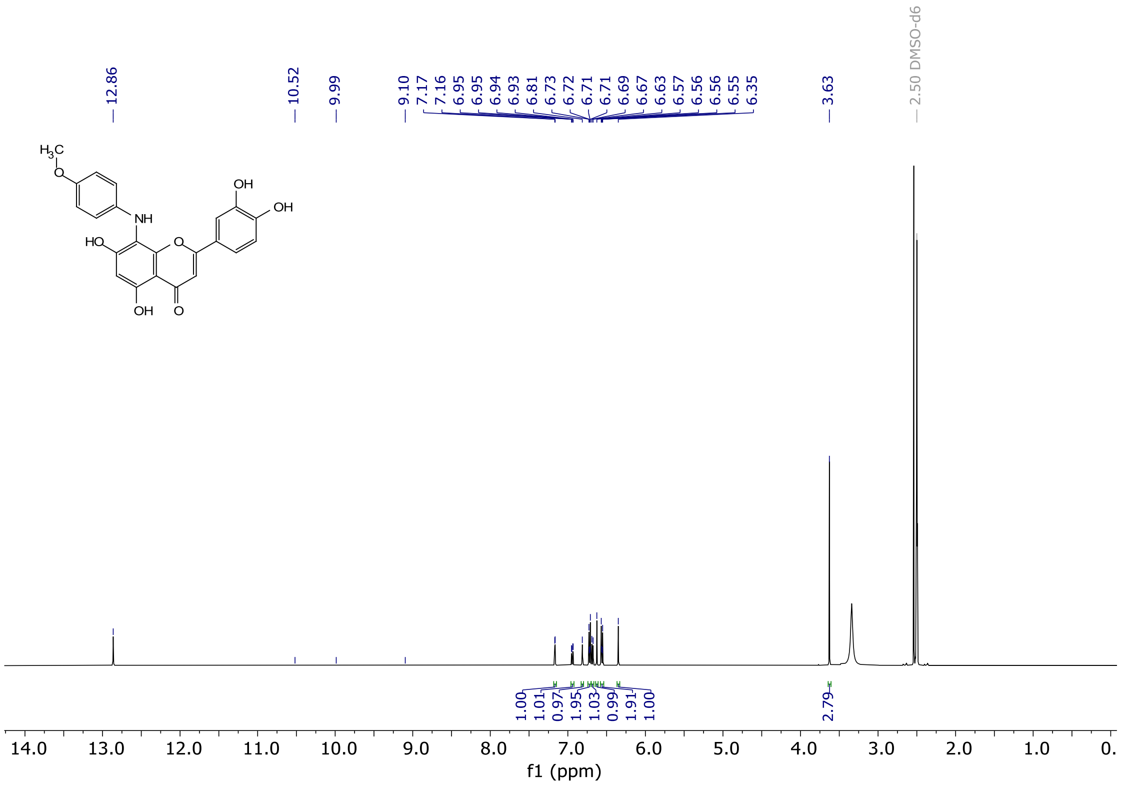


**Figure S25.** $^{1}$H NMR (500 MHz, DMSO-$d_6$, 25 °C) spectrum of 8-(4-methoxyanilino) luteolin (**12**).

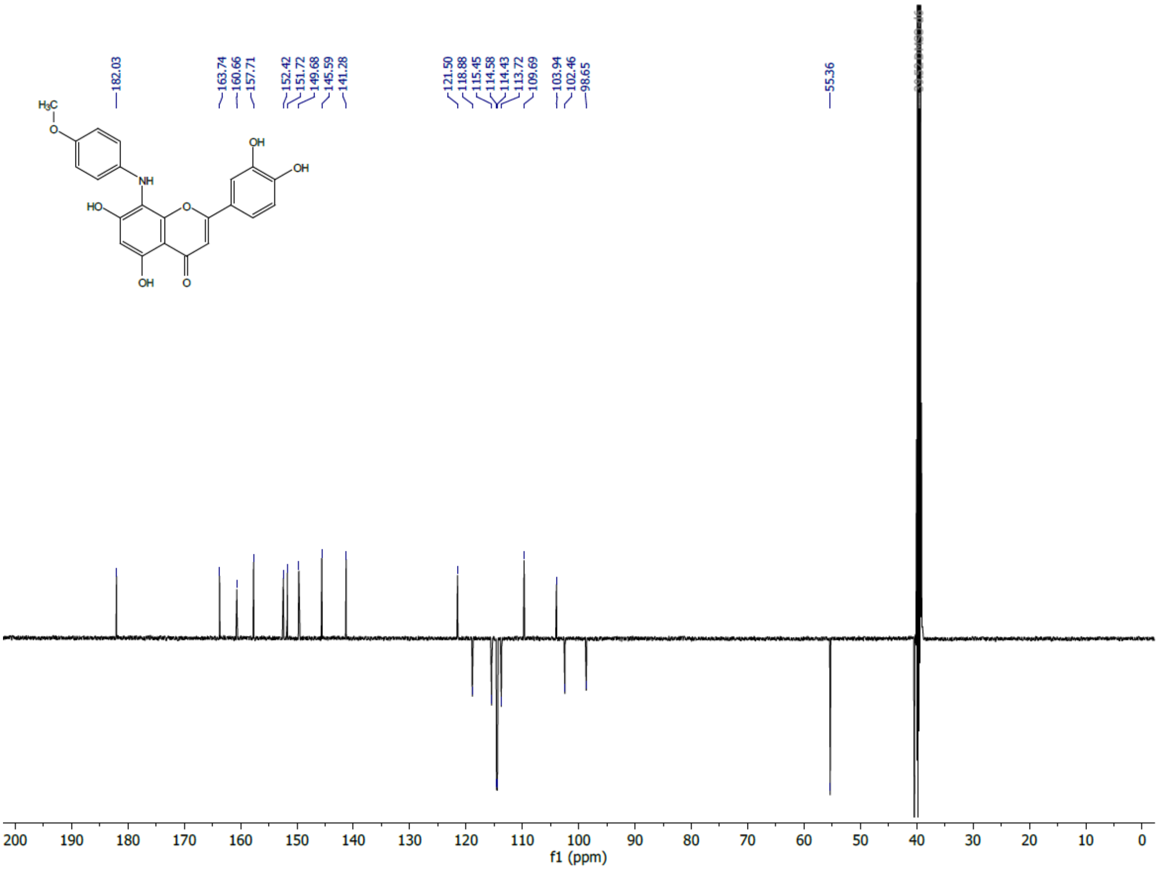


**Figure S26.** $^{13}C\{^{1}H\}$ APT NMR (126 MHz, DMSO-$d_6$, 25 °C) spectrum of 8-(4-methoxyanilino) luteolin (**12**).

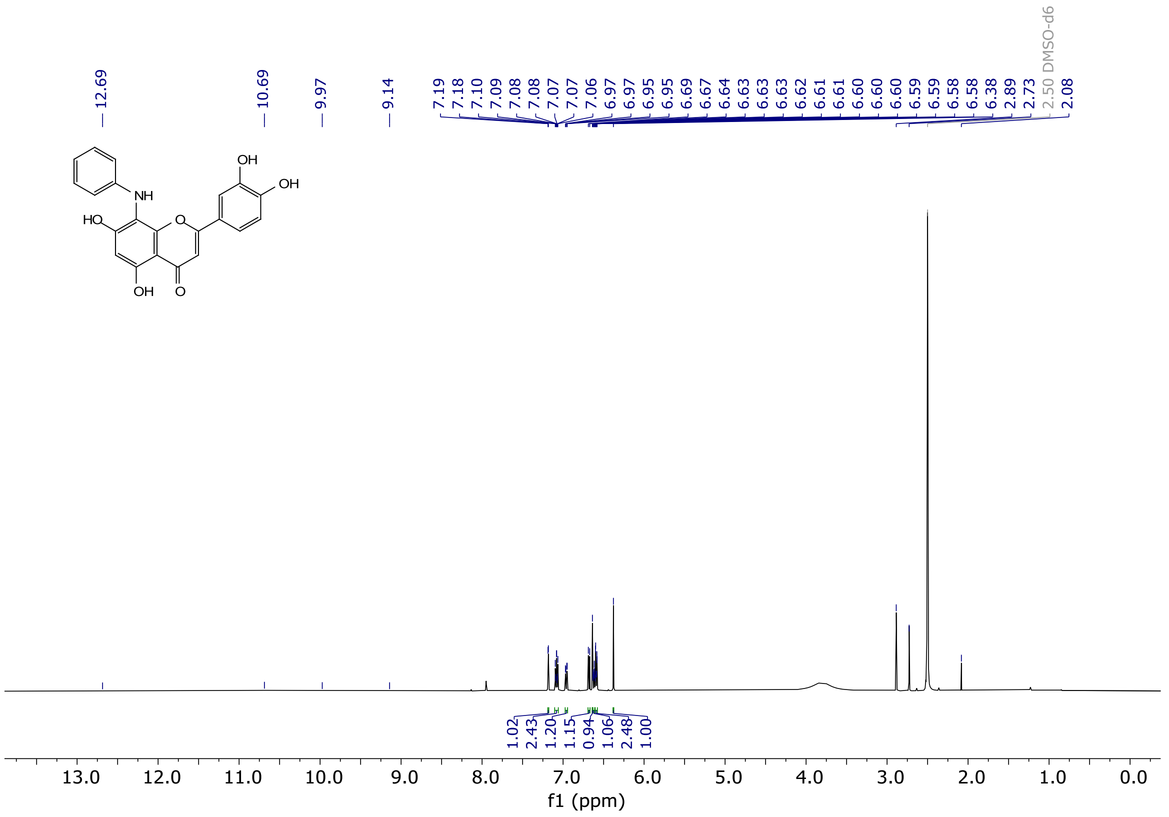

**Figure S27.** $^{1}H$ NMR (500 MHz, DMSO-$d_6$, 25 °C) spectrum of 8-(anilino) luteolin (**13**).

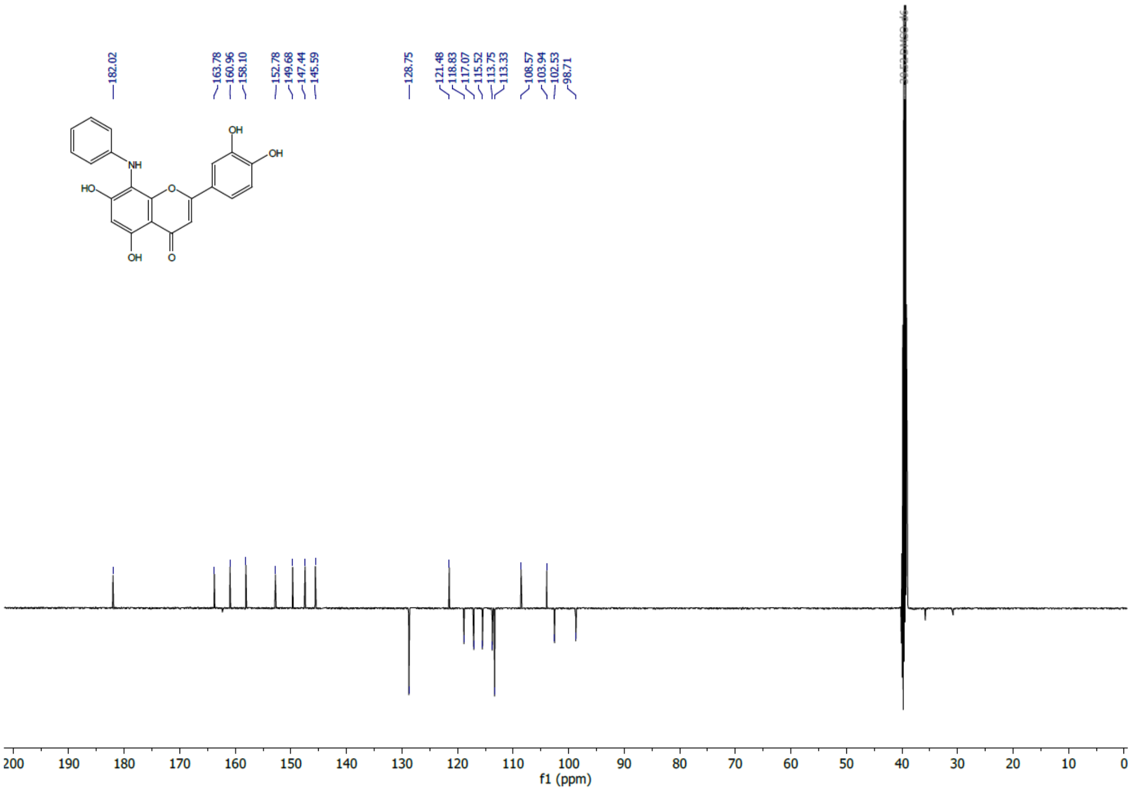

**Figure S28.** $^{13}C\{^{1}H\}$ APT NMR (126 MHz, DMSO-$d_6$, 25 °C) spectrum of 8-(anilino) luteolin (**13**).

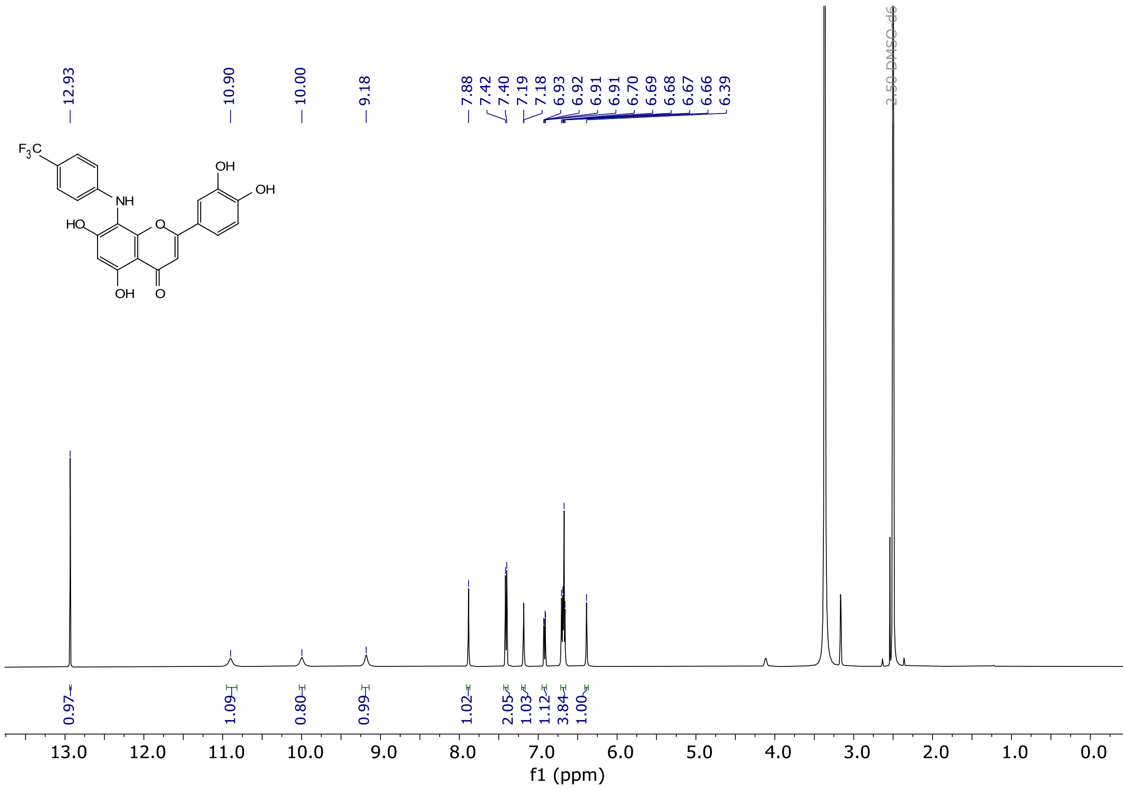

**Figure S29.** $^{1}H$ NMR (500 MHz, DMSO-$d_6$, 25 °C) spectrum of 8-(4-(trifluoromethyl)anilino) luteolin (**14**).

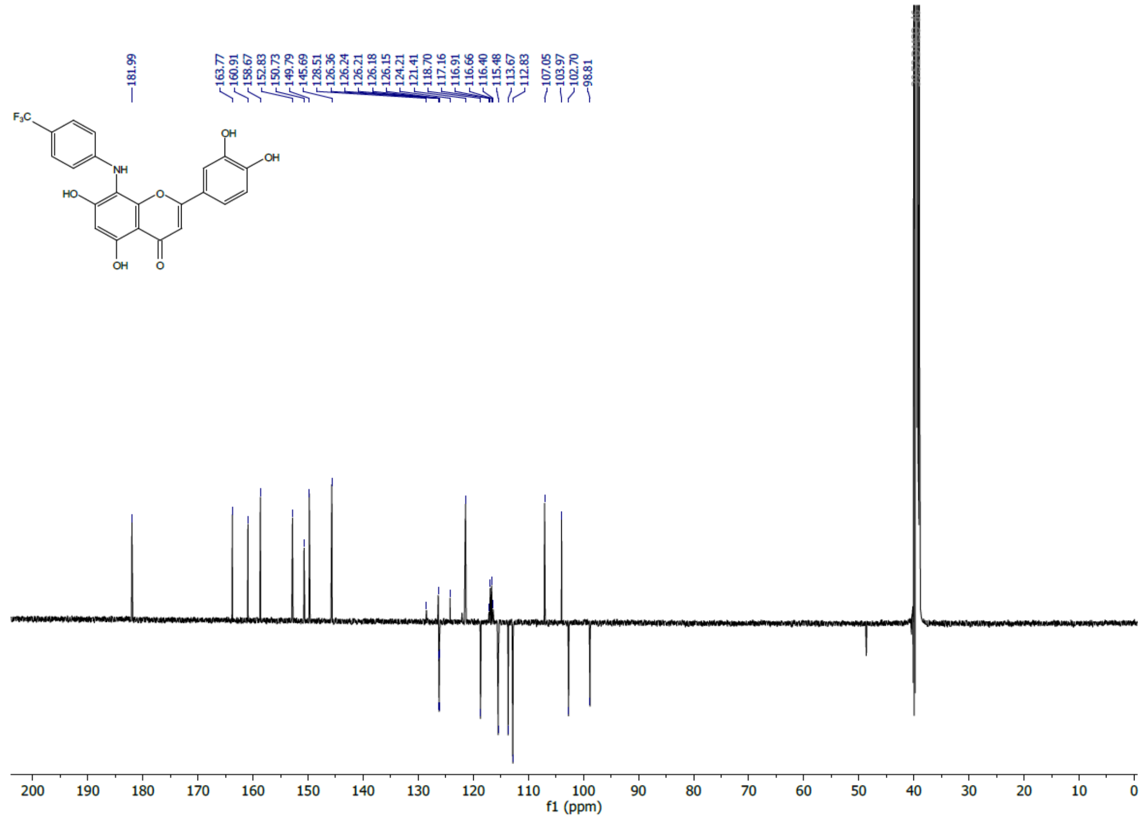

**Figure S30.** $^{13}C\{^{1}H\}$ APT NMR (126 MHz, DMSO-$d_6$, 25 °C) spectrum of 8-(4-(trifluoromethyl)anilino) luteolin (**14**).

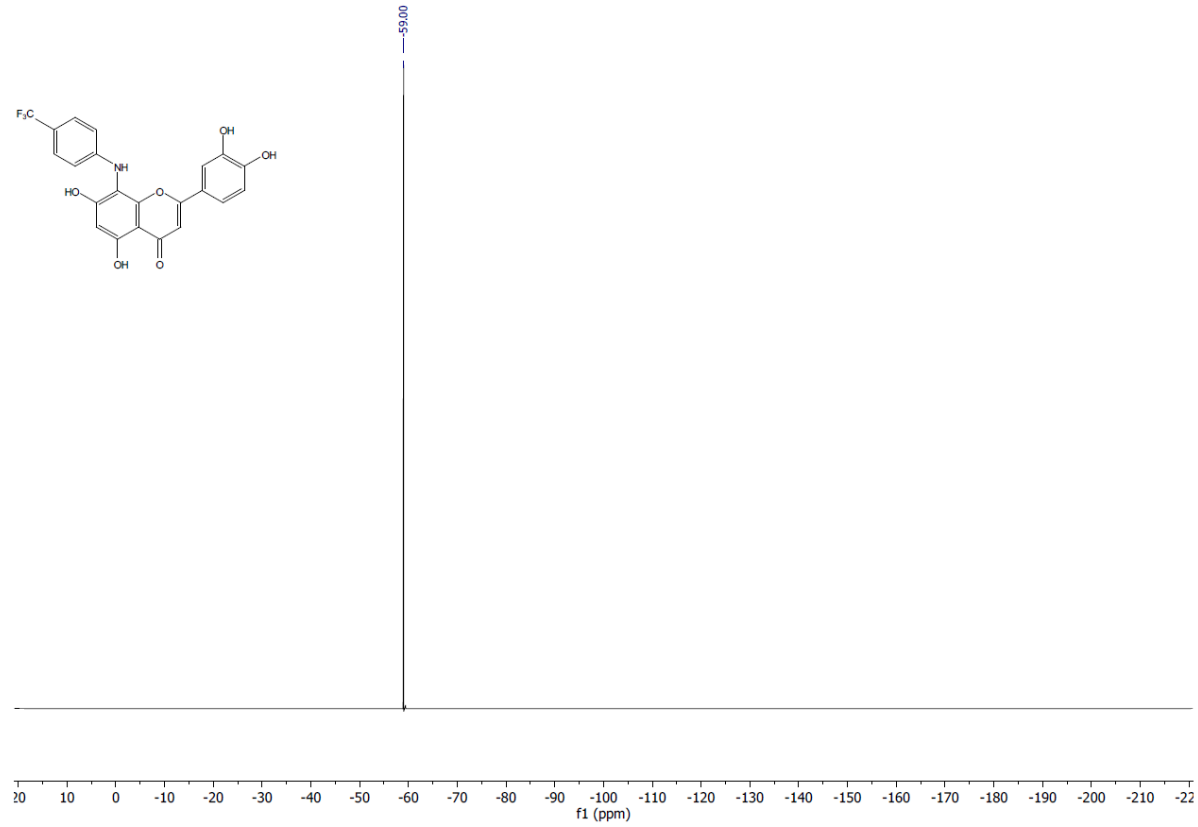


**Figure S31.** $^{19}$F NMR (470 MHz, DMSO-$d_6$, 25 °C) spectrum of 8-(4-(trifluoromethyl)anilino) luteolin (**14**).

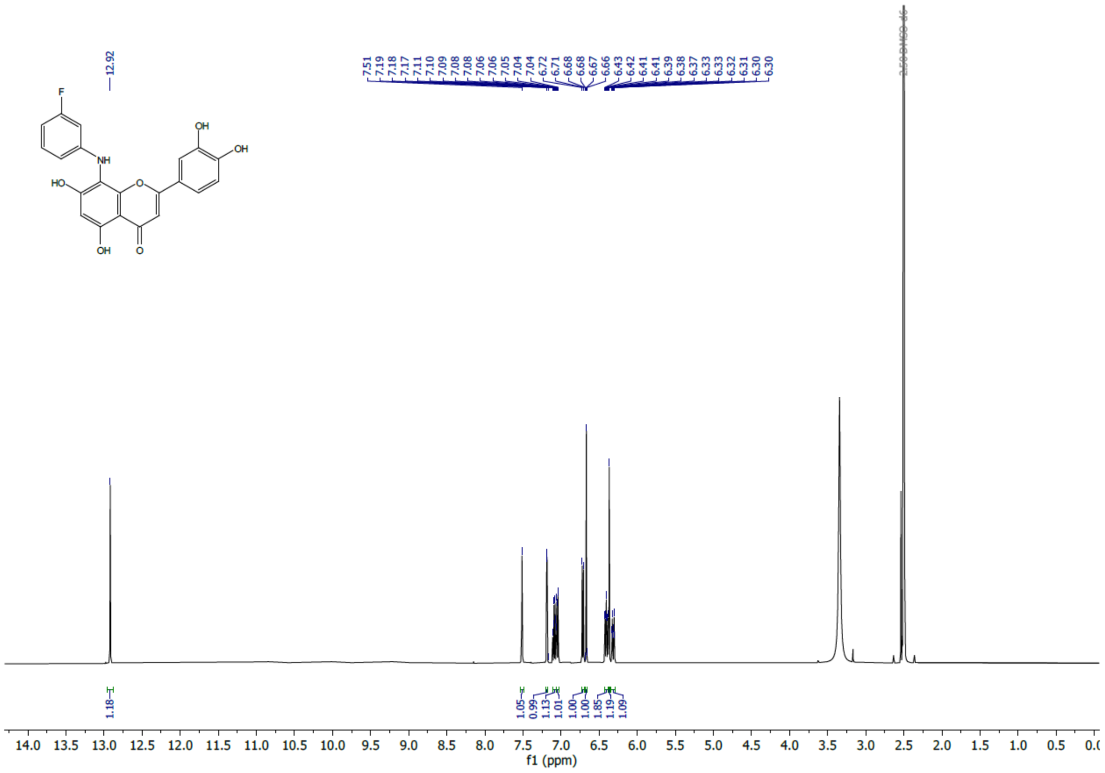

**Figure S32.** $^{1}H$ NMR (500 MHz, DMSO-$d_6$, 25 °C) spectrum of 8-(3-fluoroanilino) luteolin (**15**).

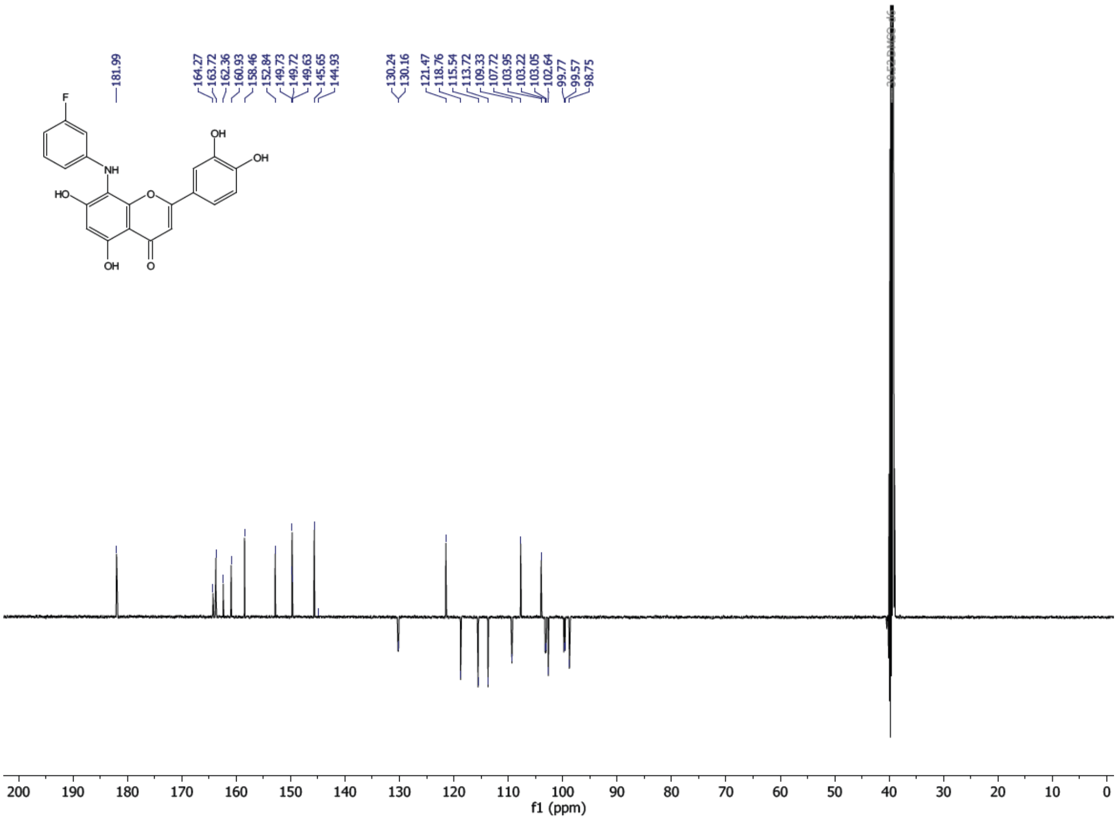

**Figure S33.** $^{13}C\{^{1}H\}$ APT NMR (126 MHz, DMSO-$d_6$, 25 °C) spectrum of 8-(3-fluoroanilino) luteolin (**15**).

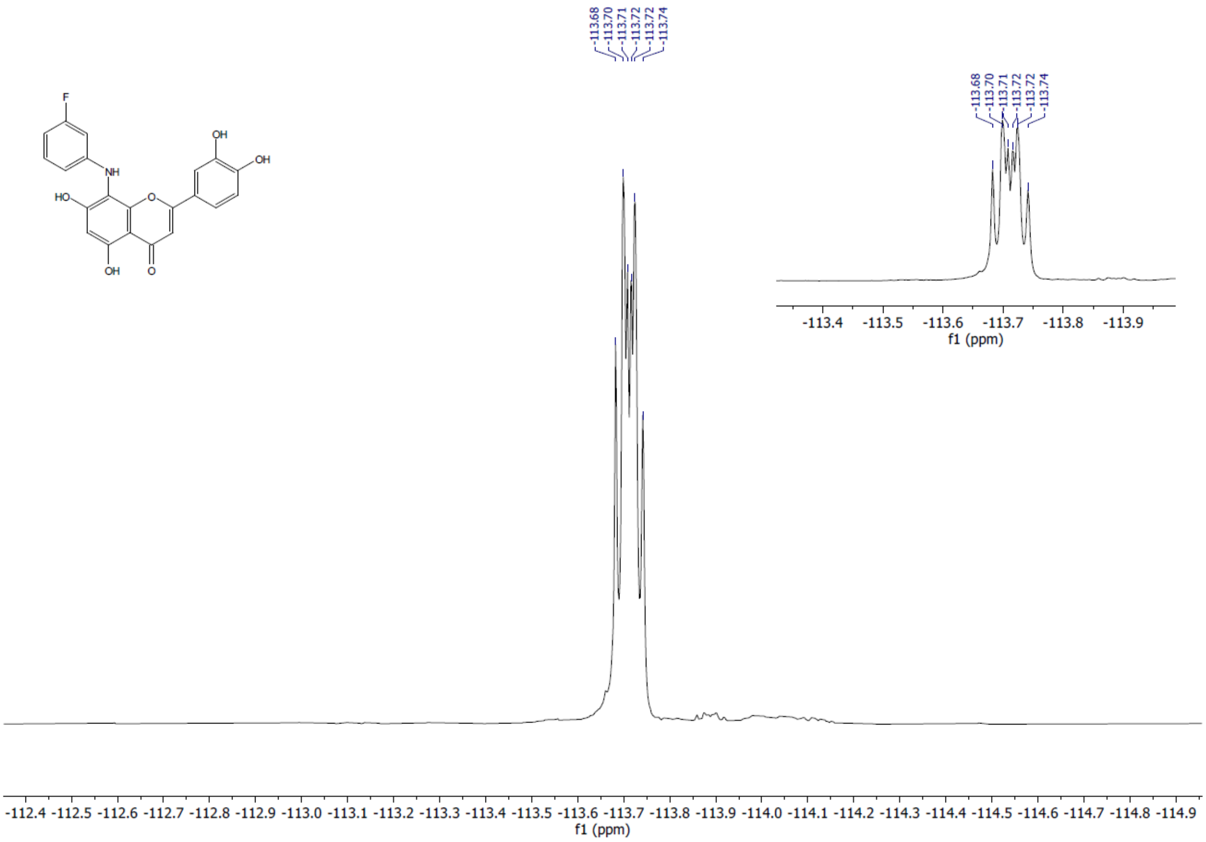


**Figure S34.** $^{19}$F NMR (470 MHz, DMSO-$d_6$, 25 °C) spectrum of 8-(3-fluoroanilino) luteolin (**15**).

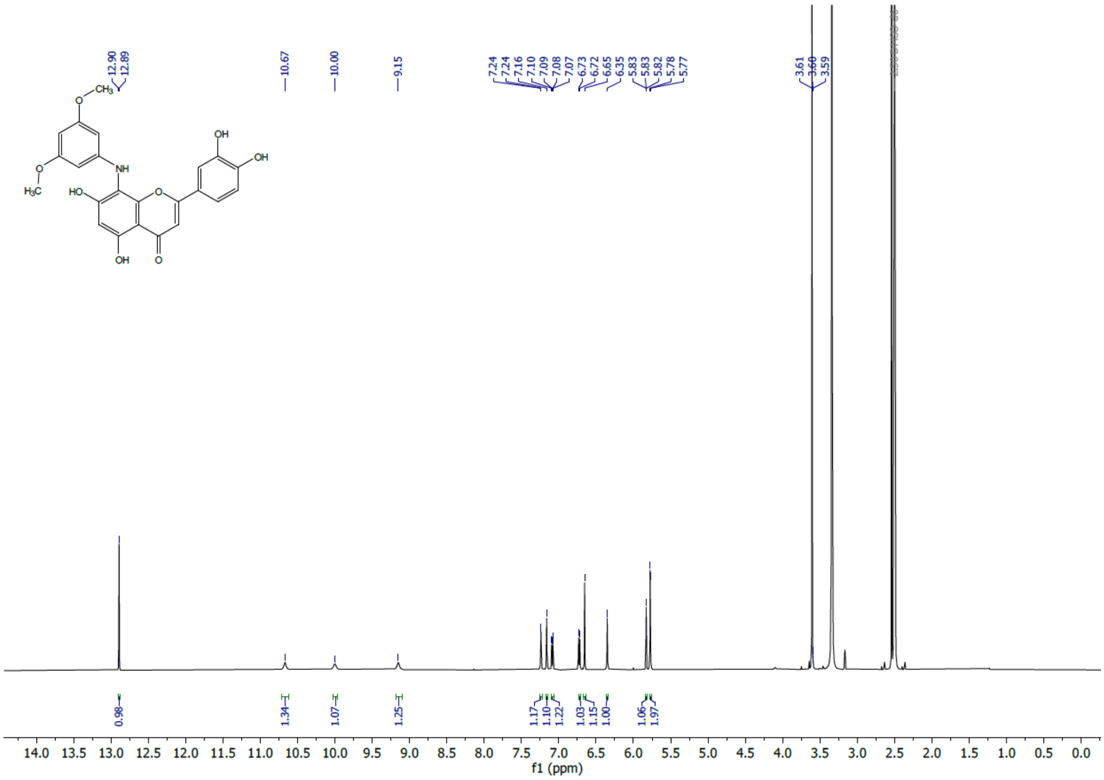


**Figure S35.** $^{1}H$ NMR (500 MHz, DMSO-$d_6$, 25 °C) spectrum of 8-(3,5-dimethoxyanilino) luteolin (**16**).

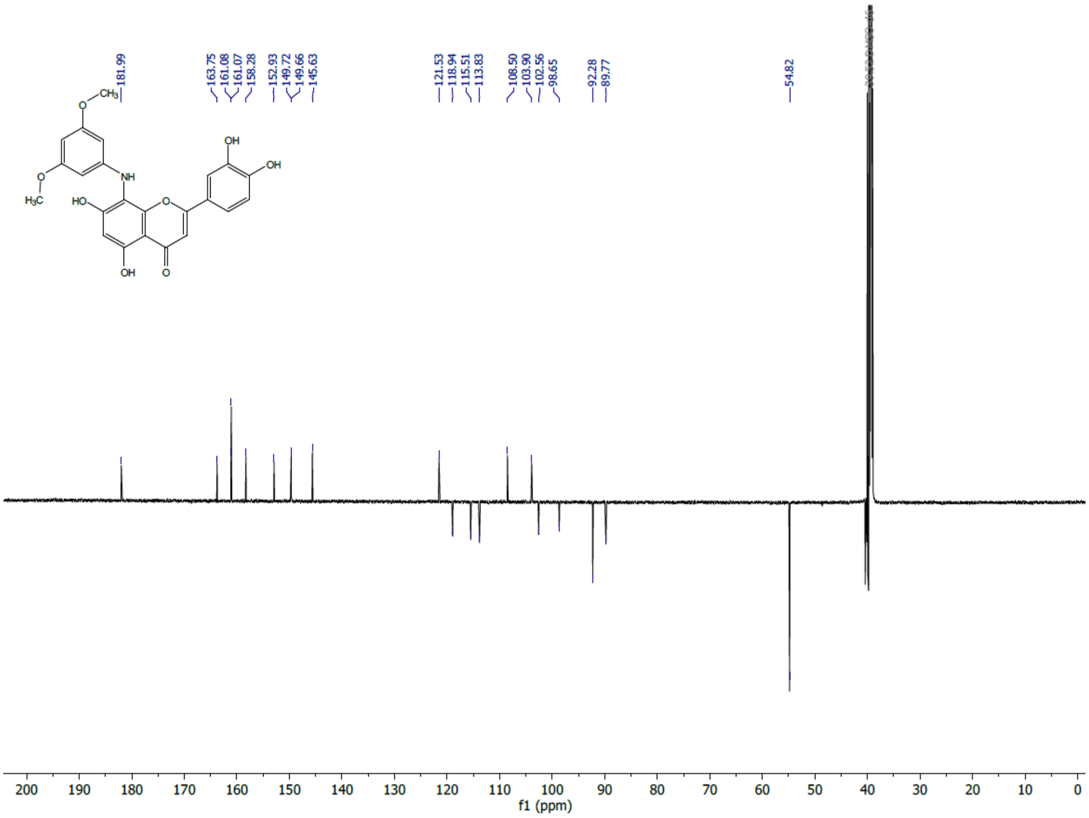


**Figure S36.** $^{13}C\{^{1}H\}$ APT NMR (126 MHz, DMSO-$d_6$, 25 °C) spectrum of 8-(3,5-dimethoxyanilino) luteolin (**16**).

## 4. HPLC ANALYSES

<Chromatogram>

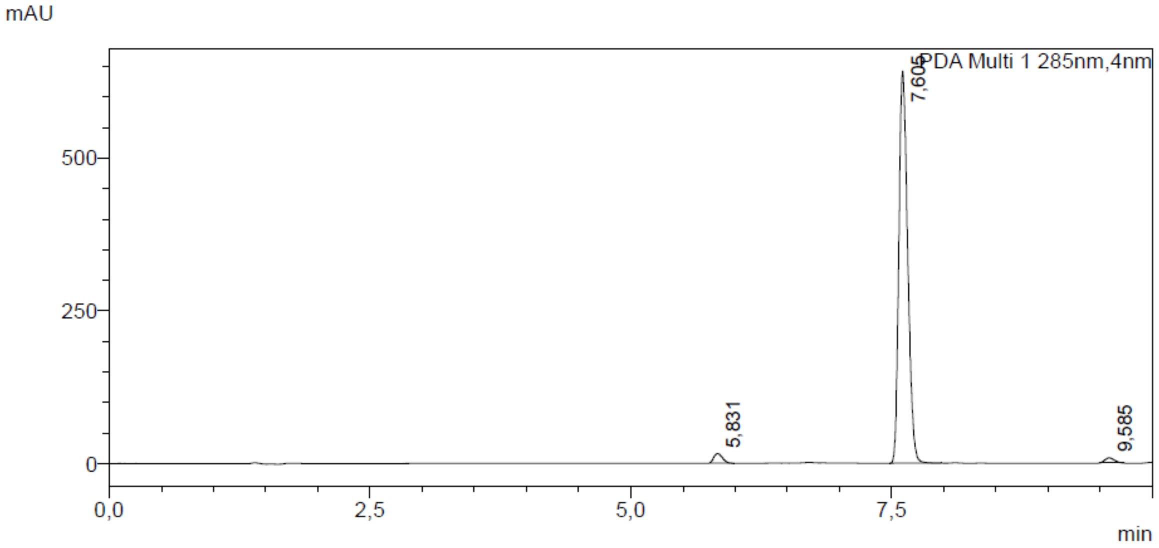


Peak Table

PDA Ch1 285nm

| Peak# | Ret. Time | Area | Height | Area% |
|---|---|---|---|---|
| 1 | 5,831 | 86168 | 15222 | 2,203 |
| 2 | 7,605 | 3775712 | 639780 | 96,512 |
| 3 | 9,585 | 50295 | 8170 | 1,286 |
| Total | | 3912175 | 663171 | 100,000 |

**Figure S37.** HPLC chromatogram of 6-hexylamino flavone (**2**) detected at 285 nm. Arion Polar C18, 0.4 mL/min, 25 °C. Product peak at 7.605 min, purity 97%.

<Chromatogram>

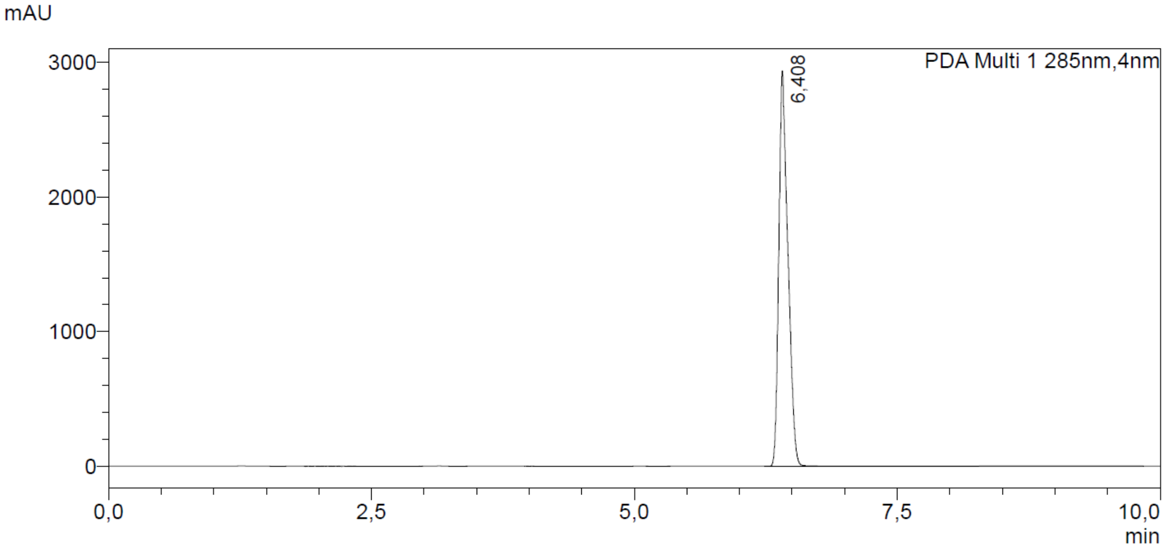


Peak Table

PDA Ch1 285nm

| Peak# | Ret. Time | Area | Height | Area% |
|---|---|---|---|---|
| 1 | 6,408 | 17675832 | 2919866 | 100,000 |
| Total | | 17675832 | 2919866 | 100,000 |

**Figure S38.** HPLC chromatogram of 8-(4-methoxyanilino) flavone (**3**) detected at 285 nm. Arion Polar C18, 0.4 mL/min, 25 °C. Product peak at 6.408 min, purity 100%.

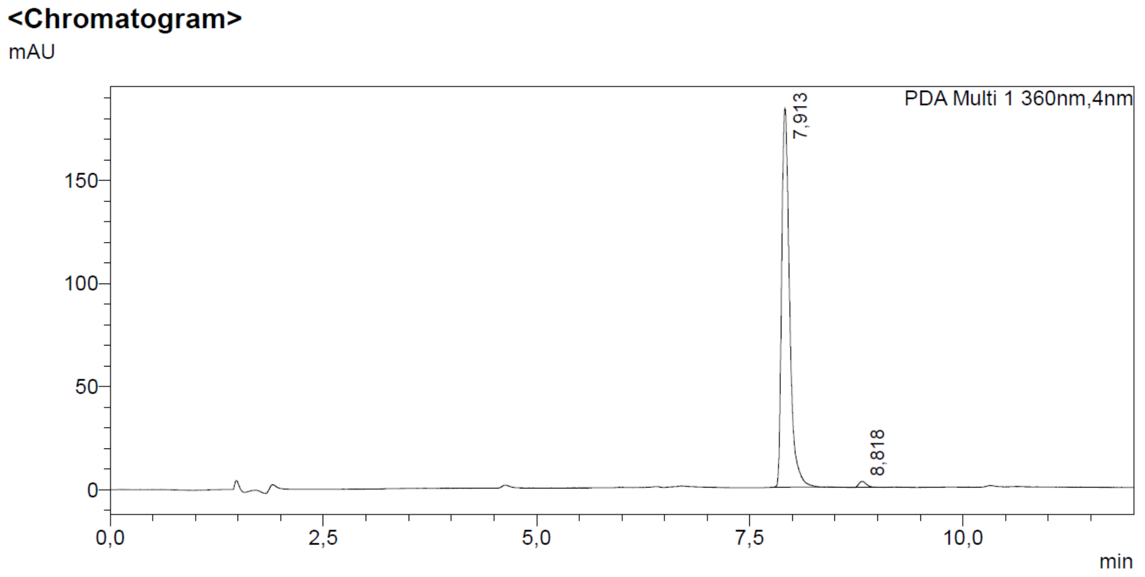


Peak Table

PDA Ch1 360nm

| Peak# | Ret. Time | Area | Height | Area% |
|---|---|---|---|---|
| 1 | 7.913 | 1190805 | 183083 | 98.575 |
| 2 | 8.818 | 17209 | 2846 | 1.425 |
| Total | | 1208014 | 185929 | 100.000 |

**Figure S39.** HPLC chromatogram of 8-(4-fluoroanilino) quercetin (**5**) detected at 360 nm. Arion Polar C18, 0.4 mL/min, 25 °C. Product peak at 7.913 min, purity 99%.

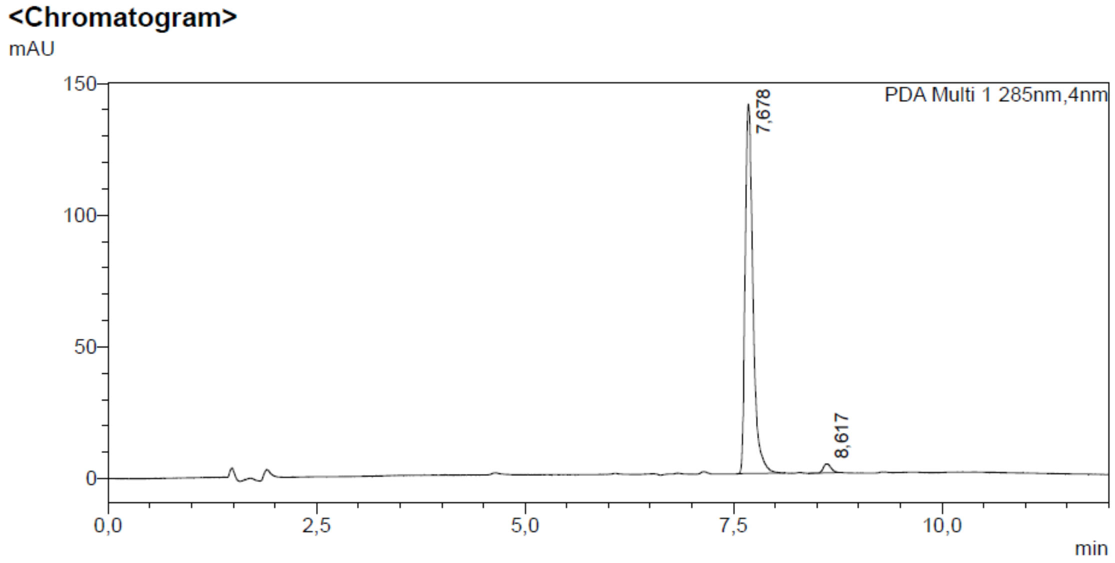


Peak Table

PDA Ch1 285nm

| Peak# | Ret. Time | Area | Height | Area% |
|---|---|---|---|---|
| 1 | 7.678 | 913678 | 139839 | 97.768 |
| 2 | 8.617 | 20863 | 3354 | 2.232 |
| Total | | 934541 | 143192 | 100.000 |

**Figure S40.** HPLC chromatogram of 8-(4-methoxyanilino) quercetin (**6**) detected at 285 nm. Arion Polar C18, 0.4 mL/min, 25 °C. Product peak at 7.678 min, purity 98%.

**<Chromatogram>**

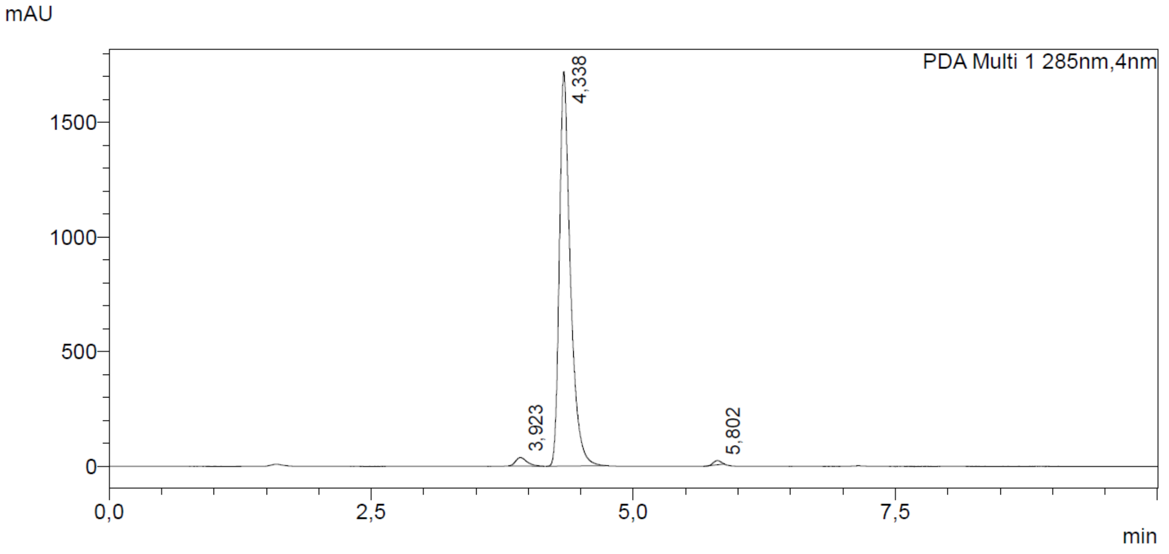


Peak Table

PDA Ch1 285nm

| Peak# | Ret. Time | Area | Height | Area% |
|---|---|---|---|---|
| 1 | 3,923 | 273044 | 36632 | 2,111 |
| 2 | 4,338 | 12567926 | 1717705 | 97,176 |
| 3 | 5,802 | 92141 | 18706 | 0,712 |
| Total | | 12933111 | 1773043 | 100,000 |

**Figure S41.** HPLC chromatogram of 8-(anilino) quercetin (**7**) detected at 285 nm. Arion Polar C18, 0.4 mL/min, 25 °C. Product peak at 4.338, purity 97%.

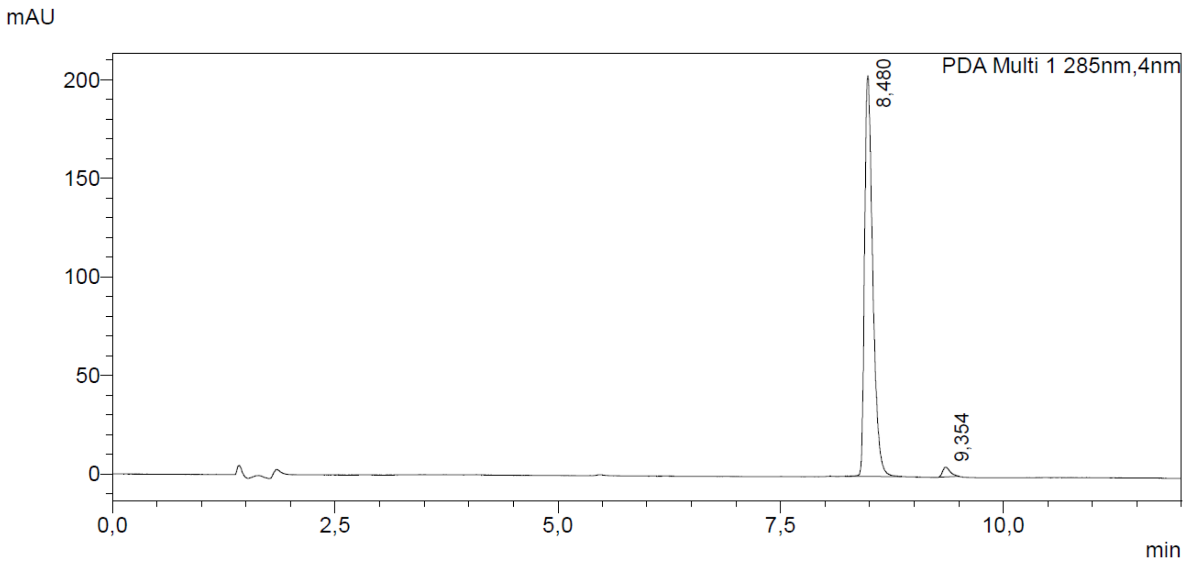


Peak Table

PDA Ch1 285nm

| Peak# | Ret. Time | Area | Height | Area% |
|---|---|---|---|---|
| 1 | 8,480 | 1291370 | 202526 | 97,845 |
| 2 | 9,354 | 28448 | 4829 | 2,155 |
| Total | | 1319817 | 207356 | 100,000 |

**Figure S42.** HPLC chromatogram of 8-(4-(trifluoromethyl)anilino) quercetin (**8**) detected at 285 nm. Arion Polar C18, 0.4 mL/min, 25 °C. Product peak at 8.480, purity 98%.

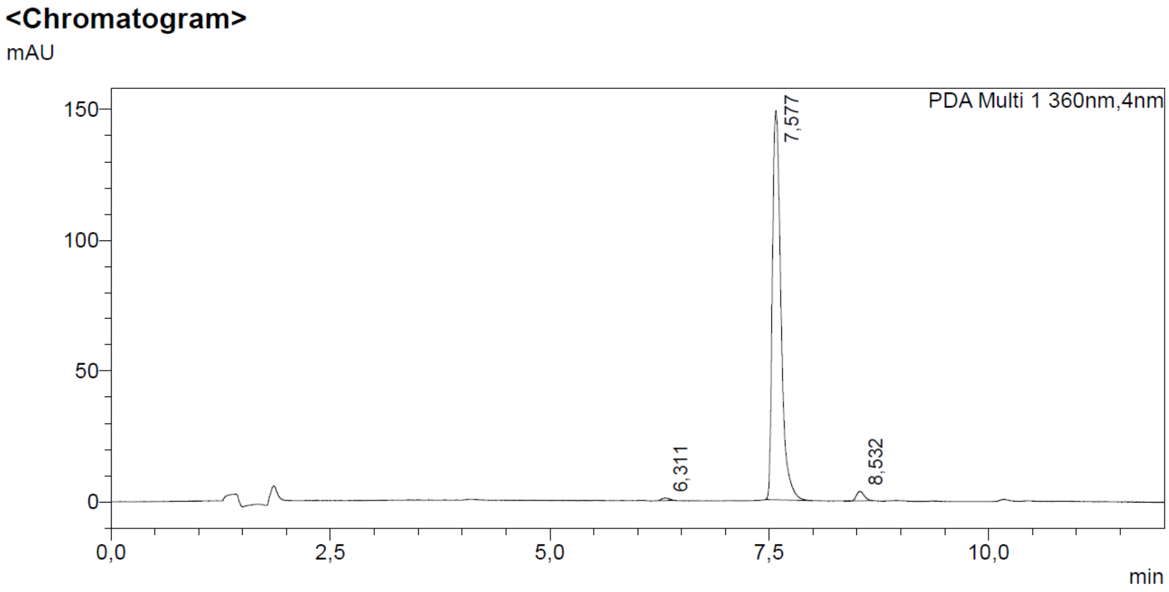


Peak Table

PDA Ch1 360nm

| Peak# | Ret. Time | Area | Height | Area% |
|---|---|---|---|---|
| 1 | 6,311 | 5733 | 976 | 0,550 |
| 2 | 7,577 | 1014435 | 148550 | 97,363 |
| 3 | 8,532 | 21738 | 3585 | 2,086 |
| Total | | 1041907 | 153112 | 100,000 |

**Figure S43.** HPLC chromatogram of 8-(3-fluoromethylanilino) quercetin (**17**) detected at 360 nm. Arion Polar C18, 0.4 mL/min, 25 °C. Product peak at 7.577 min, purity 97%.

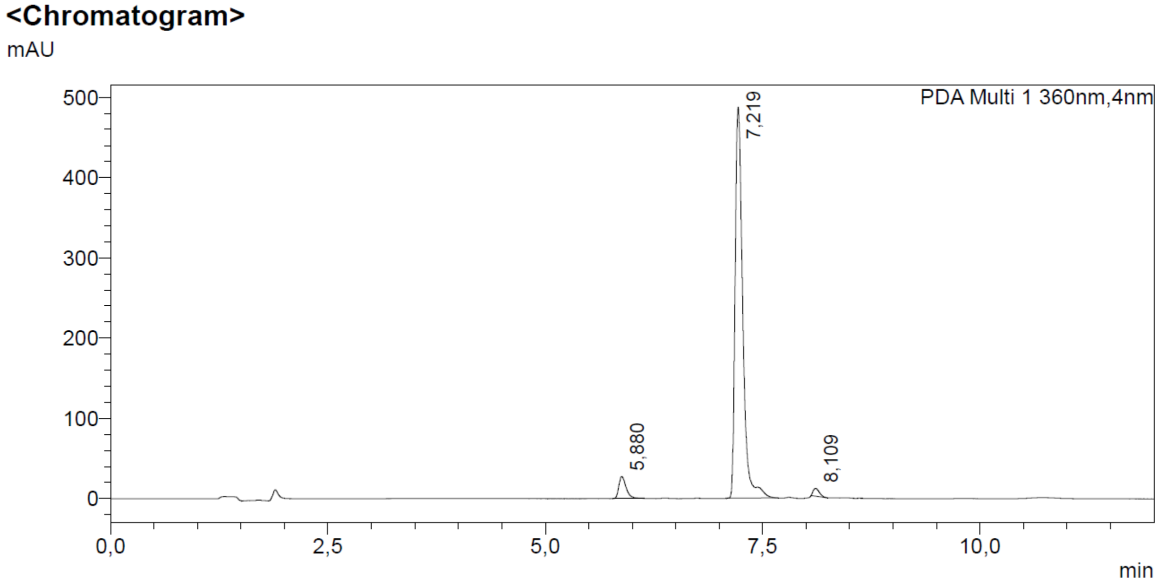


Peak Table

PDA Ch1 360nm

| Peak# | Ret. Time | Area | Height | Area% |
|---|---|---|---|---|
| 1 | 5,880 | 157944 | 27321 | 4,975 |
| 2 | 7,219 | 2969497 | 485690 | 93,539 |
| 3 | 8,109 | 47153 | 9673 | 1,485 |
| Total | | 3174594 | 522685 | 100,000 |

**Figure S44.** HPLC chromatogram of 8-(3,5-dimethoxyanilino) quercetin (**9**) detected at 360 nm. Arion Polar C18, 0.4 mL/min, 25 °C. Product peak at 7.219 min, purity 94%.

<Chromatogram>

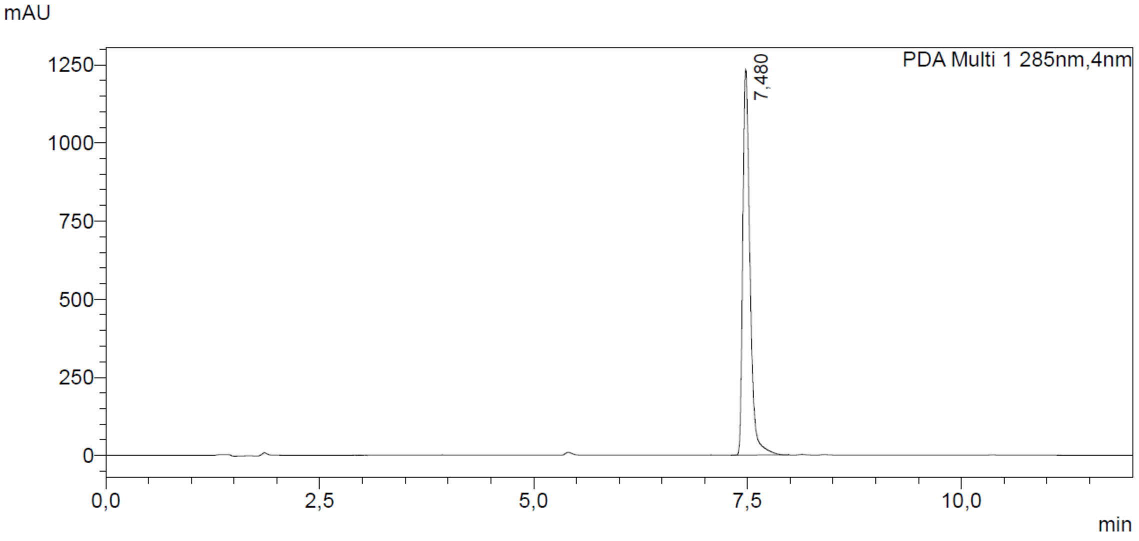


Peak Table

PDA Ch1 285nm

| Peak# | Ret. Time | Area | Height | Area% |
|---|---|---|---|---|
| 1 | 7.480 | 7236826 | 1228119 | 100.000 |
| Total | | 7236826 | 1228119 | 100.000 |

**Figure S45.** HPLC chromatogram of 8-(4-fluoroanilino) luteolin (**11**) detected at 285 nm. Arion Polar C18, 0.4 mL/min, 25 °C. Product peak at 7.480 min, purity 100%.

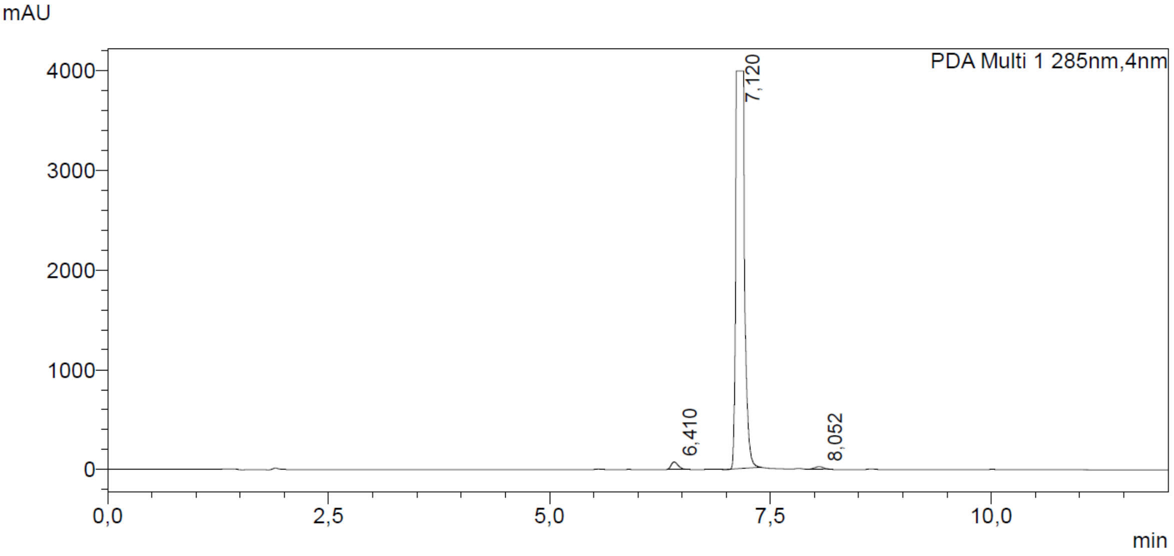


Peak Table

PDA Ch1 285nm

| Peak# | Ret. Time | Area | Height | Area% |
|---|---|---|---|---|
| 1 | 6.410 | 407602 | 74559 | 1.445 |
| 2 | 7.120 | 27628068 | 3992373 | 97.978 |
| 3 | 8.052 | 162562 | 24912 | 0.576 |
| Total | | 28198231 | 4091844 | 100.000 |

**Figure S46.** HPLC chromatogram of 8-(4-methoxyanilino) luteolin (**12**) detected at 285 nm. Arion Polar C18, 0.4 mL/min, 25 °C. Product peak at 7.120 min, purity 98%.

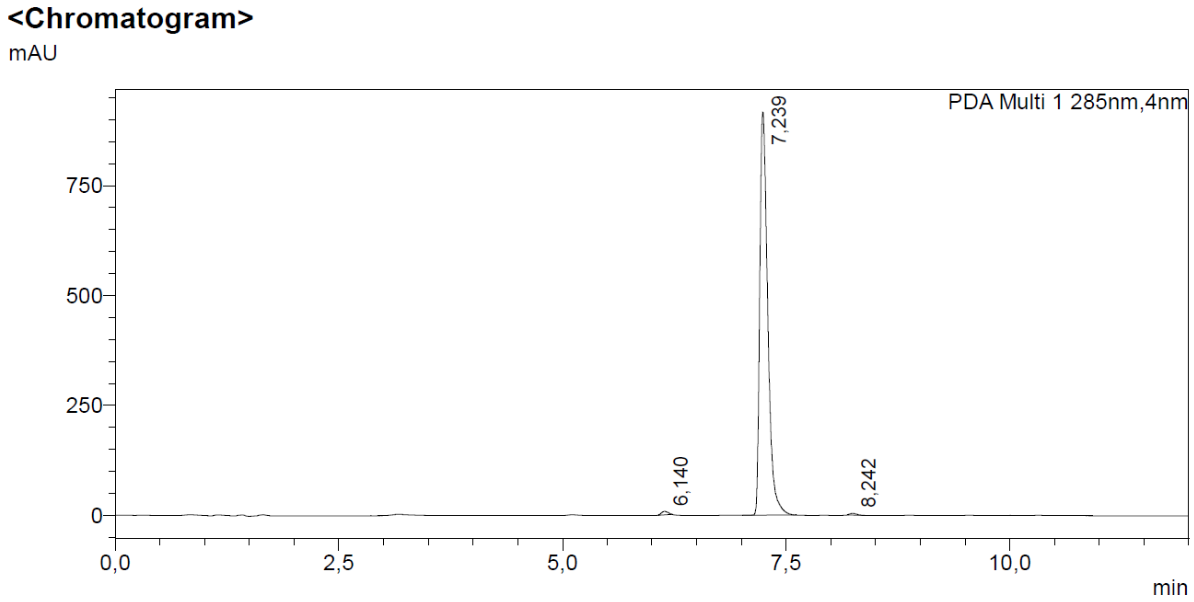


Peak Table

PDA Ch1 285nm

| Peak# | Ret. Time | Area | Height | Area% |
|---|---|---|---|---|
| 1 | 6,140 | 39029 | 7618 | 0,678 |
| 2 | 7,239 | 5696253 | 914196 | 99,011 |
| 3 | 8,242 | 17892 | 3547 | 0,311 |
| Total | | 5753174 | 925361 | 100,000 |

**Figure S47.** HPLC chromatogram of 8-(anilino) luteolin (**13**) detected at 285 nm. Arion Polar C18, 0.4 mL/min, 25 °C. Product peak at 7.239 min, purity 99%.

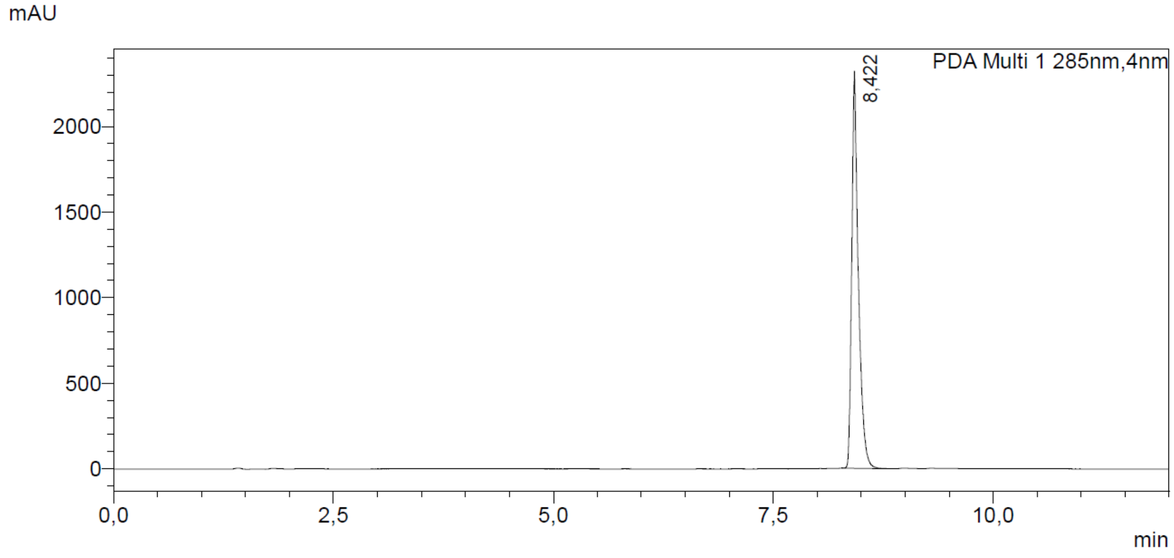


Peak Table

PDA Ch1 285nm

| Peak# | Ret. Time | Area | Height | Area% |
|---|---|---|---|---|
| 1 | 8,422 | 12074833 | 2290705 | 100,000 |
| Total | | 12074833 | 2290705 | 100,000 |

**Figure S48.** HPLC chromatogram of 8-(4-(trifluoromethyl)anilino) luteolin (**14**) detected at 285 nm. Arion Polar C18, 0.4 mL/min, 25 °C. Product peak at 8.422 min, purity 100%.

**<Chromatogram>**

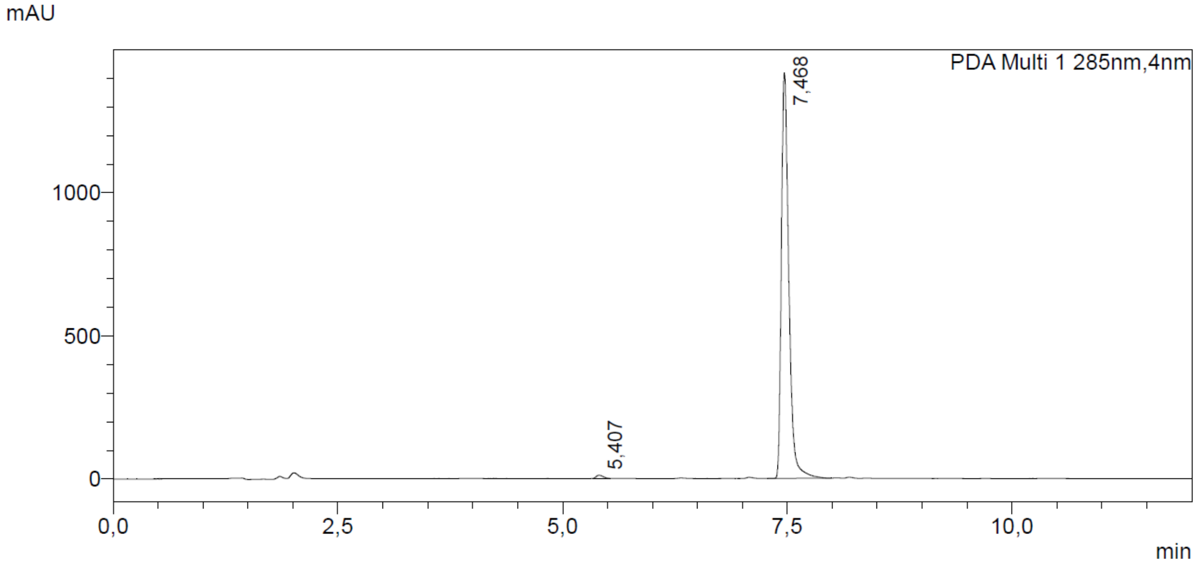


Peak Table

PDA Ch1 285nm

| Peak# | Ret. Time | Area | Height | Area% |
|---|---|---|---|---|
| 1 | 5,407 | 56740 | 10778 | 0,689 |
| 2 | 7,468 | 8174190 | 1412785 | 99,311 |
| Total | | 8230930 | 1423563 | 100,000 |

**Figure S49.** HPLC chromatogram of 8-(3-fluoroanilino) luteolin (**15**) detected at 285 nm. Arion Polar C18, 0.4 mL/min, 25 °C. Product peak at 7.468 min, purity 99%.

**<Chromatogram>**

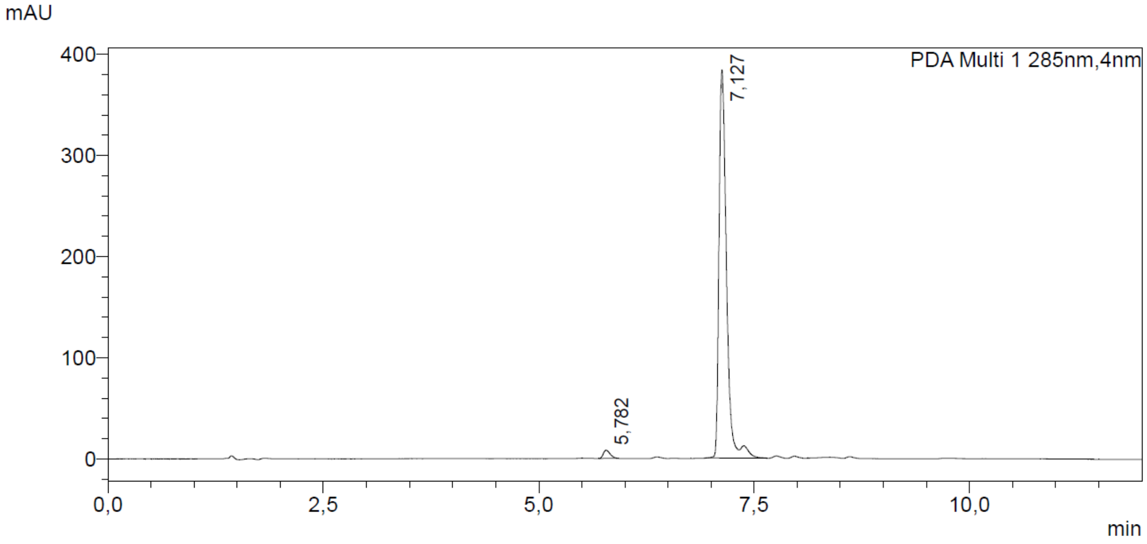


Peak Table

PDA Ch1 285nm

| Peak# | Ret. Time | Area | Height | Area% |
|---|---|---|---|---|
| 1 | 5,782 | 43689 | 8022 | 1,843 |
| 2 | 7,127 | 2326910 | 381826 | 98,157 |
| Total | | 2370598 | 389848 | 100,000 |

**Figure S50.** HPLC chromatogram of 8-(3,5-dimethoxyanilino) luteolin (**16**) detected at 285 nm. Arion Polar C18, 0.4 mL/min, 25 °C. Product peak at 7.127 min, purity 98%.

## 5. HRMS

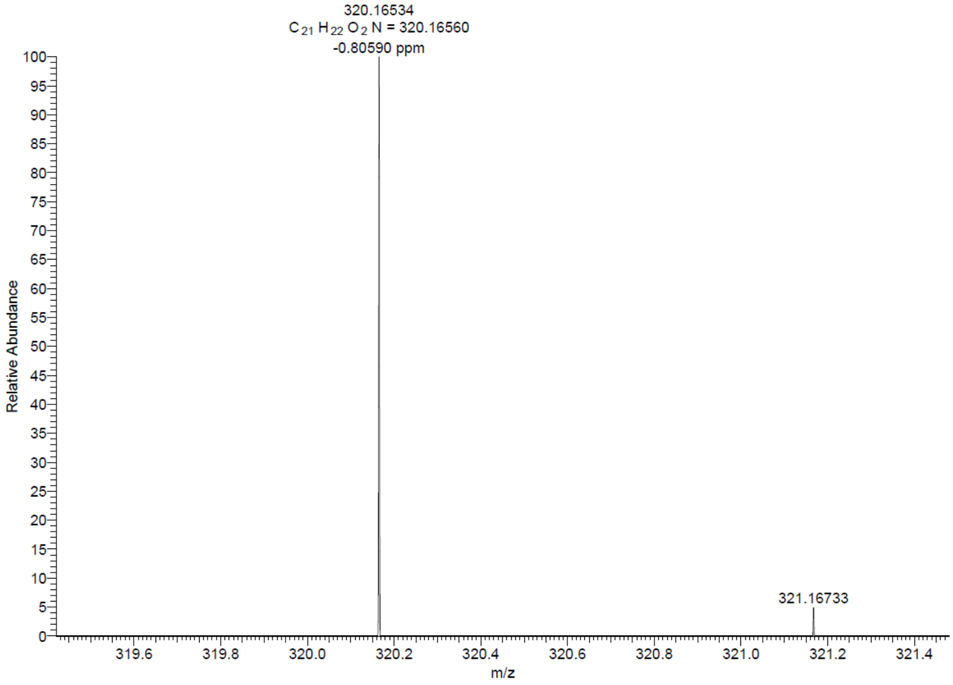


**Figure S51.** HRMS (ESI$^-$) analysis of 6-hexylamino flavone (**2**). Calculated for $C_{21}H_{22}O_2N$ [M – H]$^-$ 320.16560, measured 320.16534.

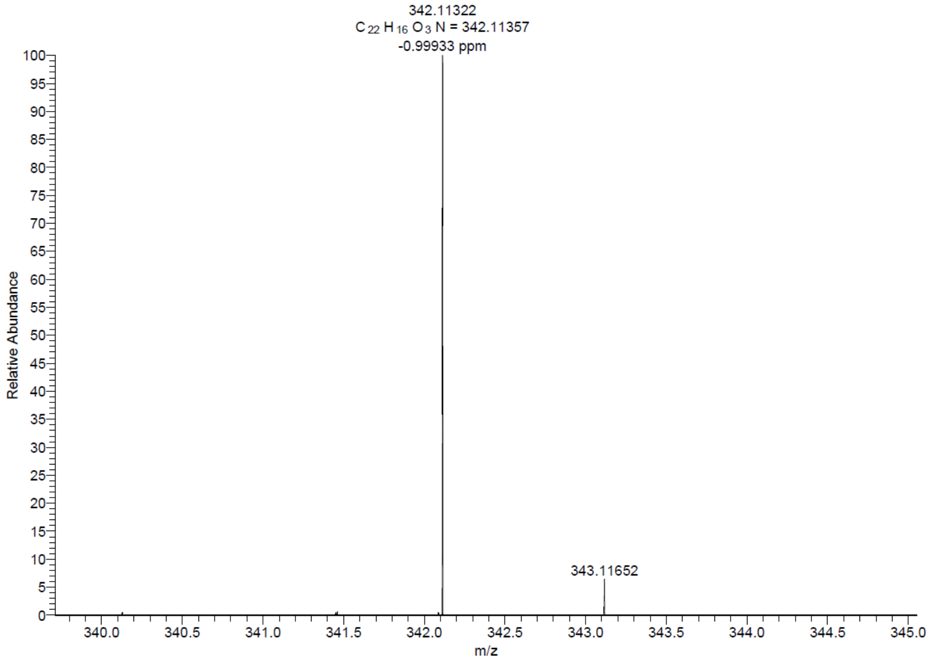


**Figure S52.** HRMS (ESI$^-$) analysis of 6-(4-methoxyanilino) flavone (**3**). Calculated for $C_{22}H_{16}O_3N$ [M – H]$^-$ 342.11357, measured 342.11322.

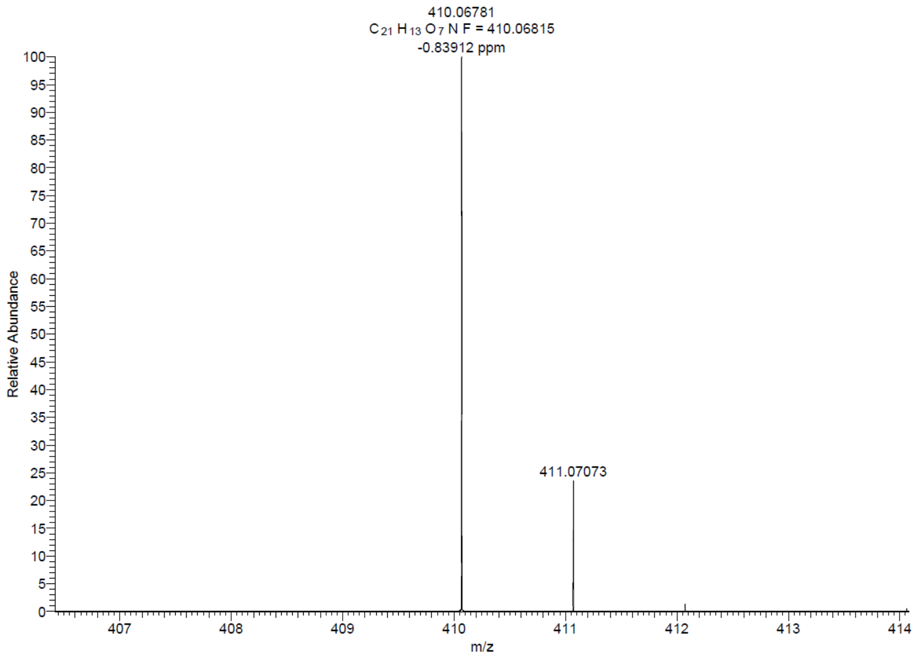


**Figure S53.** HRMS (ESI$^-$) analysis of 8-(4-fluoroanilino) quercetin (**5**). Calculated for $C_{21}H_{13}O_7F$ $[M-H]^-$ 410.06815, measured 410.06781.

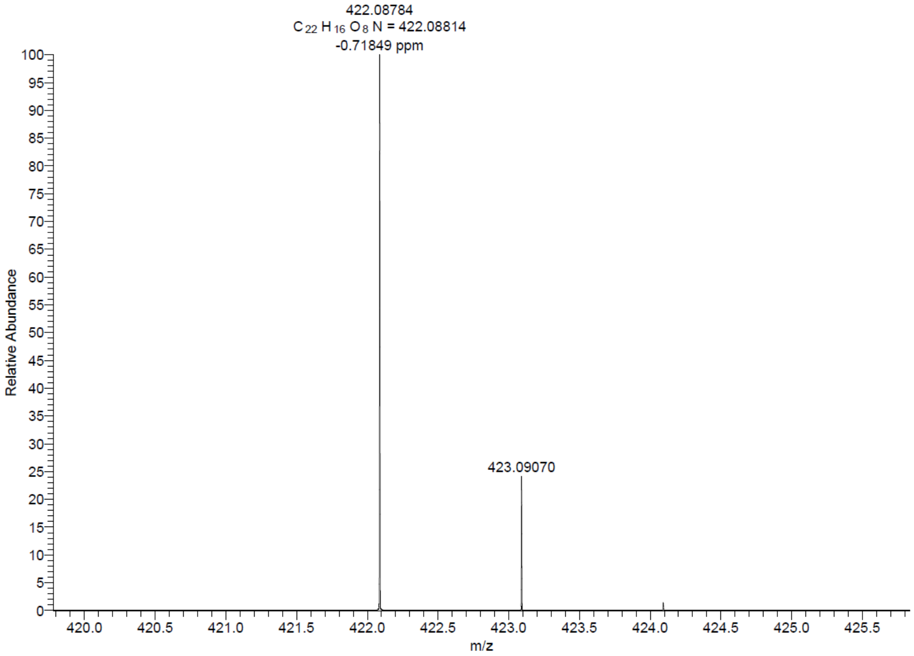


**Figure S54.** HRMS (ESI$^-$) analysis of 8-(4-methoxyanilino) quercetin (**6**). Calculated for $C_{22}H_{16}O_8N$ $[M-H]^-$ 422.08814, measured 422.08784.

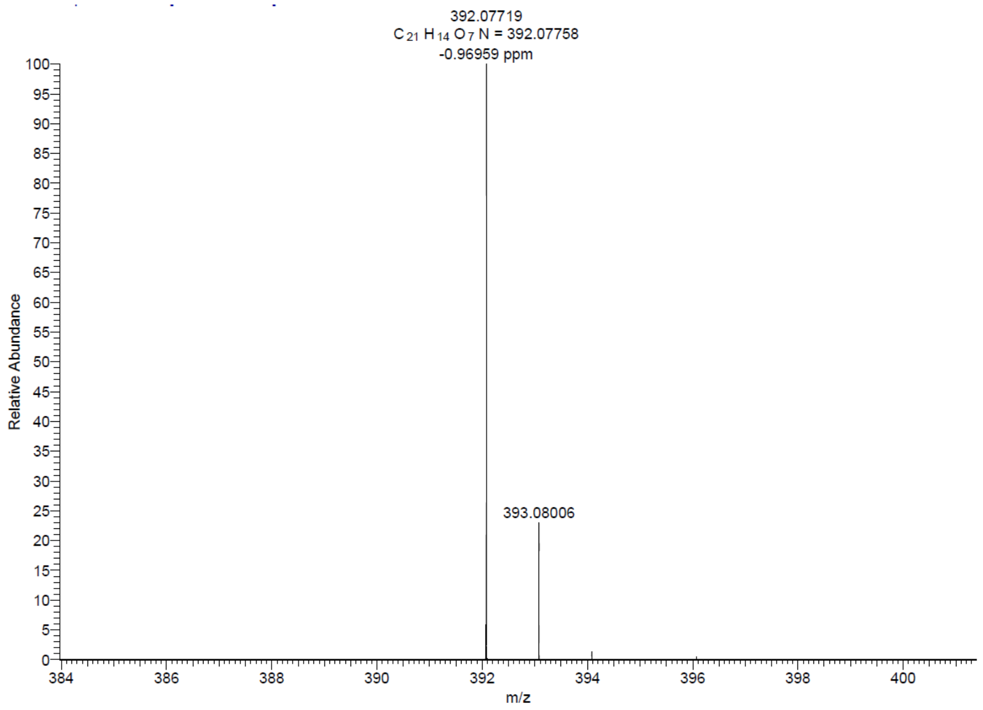


**Figure S55.** HRMS (ESI$^-$) analysis of 8-(anilino) quercetin (**7**). Calculated for $C_{21}H_{14}O_7N$ [M – H]$^-$ 392.07758, measured 392.07719.

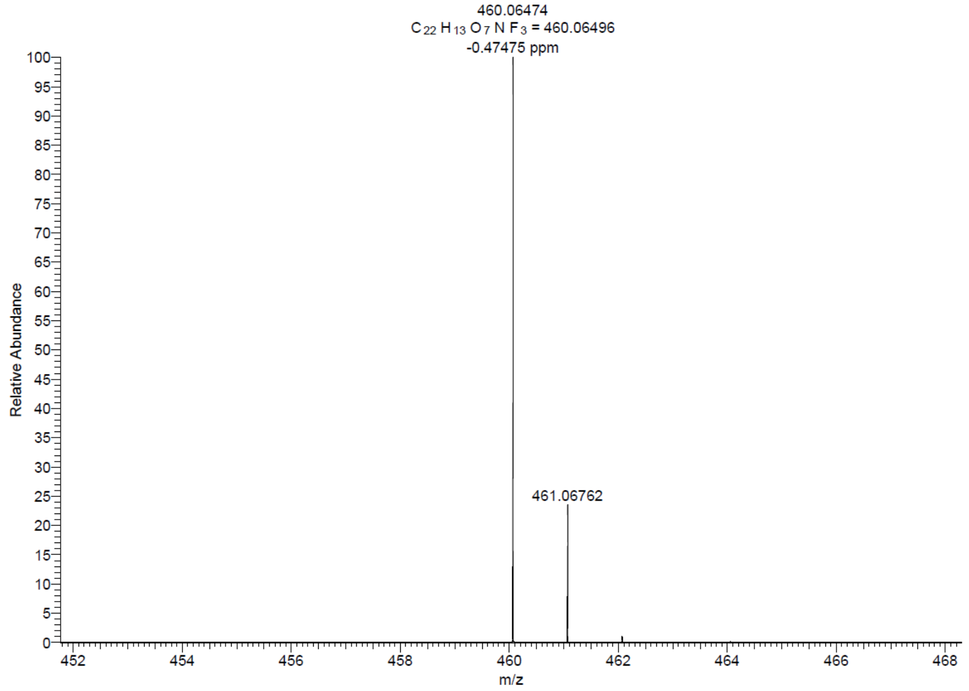


**Figure S56.** HRMS (ESI$^-$) analysis of 8-(4-(trifluoromethyl)anilino) quercetin (**8**). Calculated for $C_{22}H_{13}O_7NF_3$ [M – H]$^-$ 460.06496, measured 460.06474.

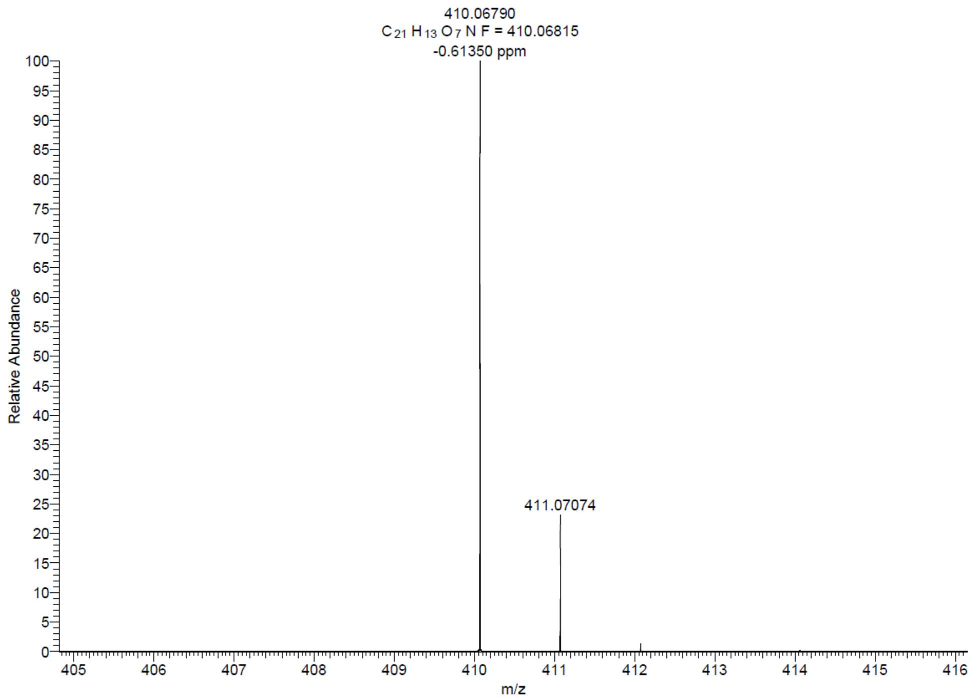


**Figure S57.** HRMS (ESI$^{-}$) analysis of 8-(3-fluoroanilino) quercetin (**17**). Calculated for $C_{21}H_{13}O_7NF$ [M – H]$^{-}$ 410.06815, measured 410.06790.

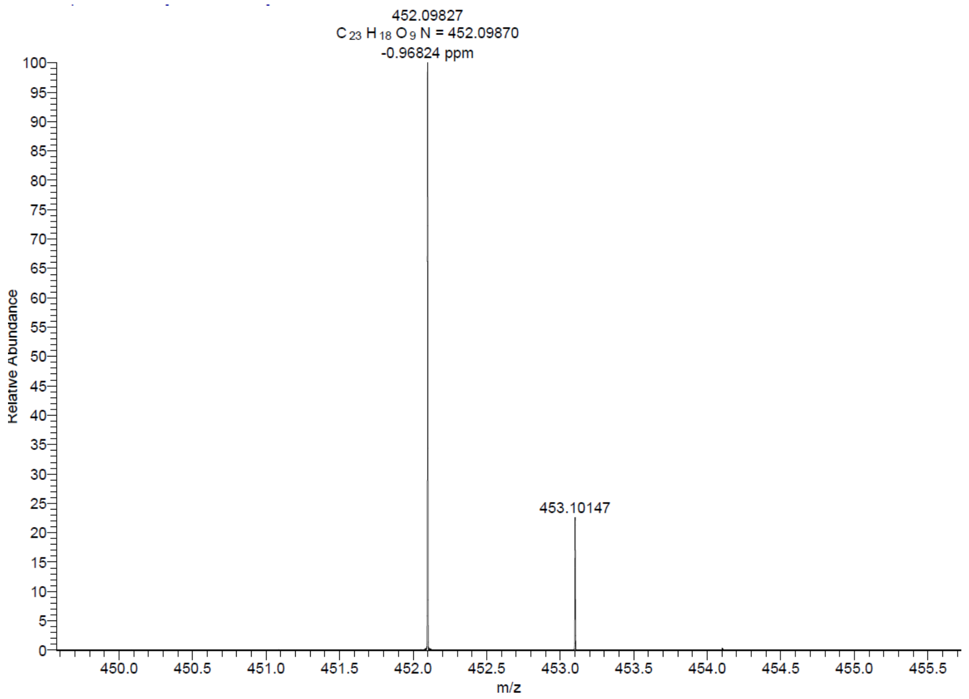


**Figure S58.** HRMS (ESI$^{-}$) analysis of 8-(3,5-dimethoxyanilino) quercetin (**9**). Calculated for $C_{23}H_{18}O_9N$ [M – H]$^{-}$ 452.09870, measured 452.09827.

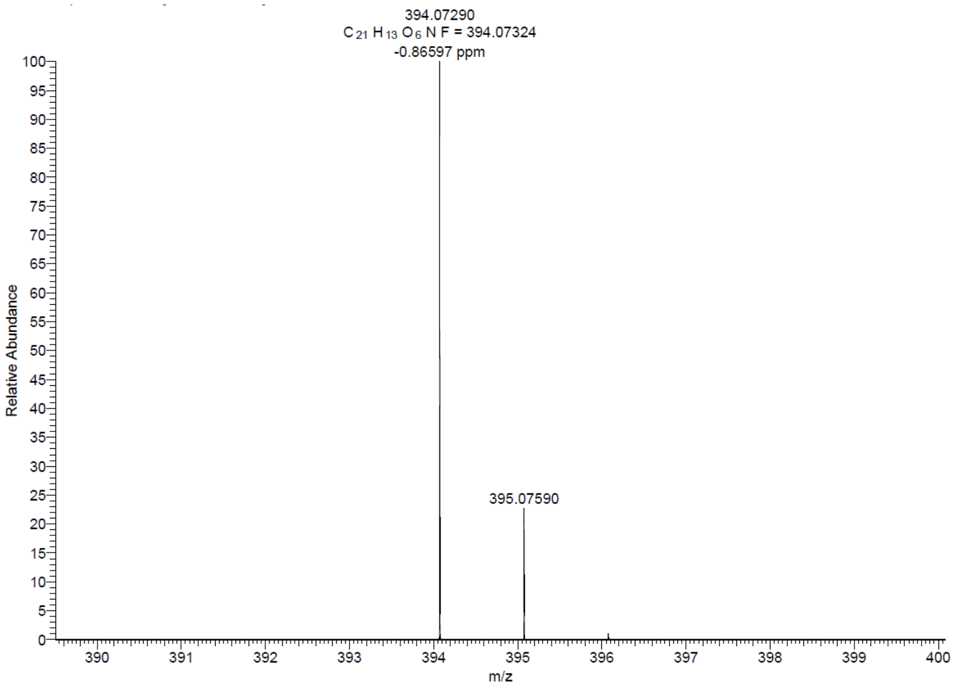


**Figure S59.** HRMS (ESI$^-$) analysis of 8-(4-fluoroanilino) luteolin (**11**). Calculated for $C_{21}H_{13}O_6NF$ [M – H]$^-$ 394.07324, measured 394.07290.

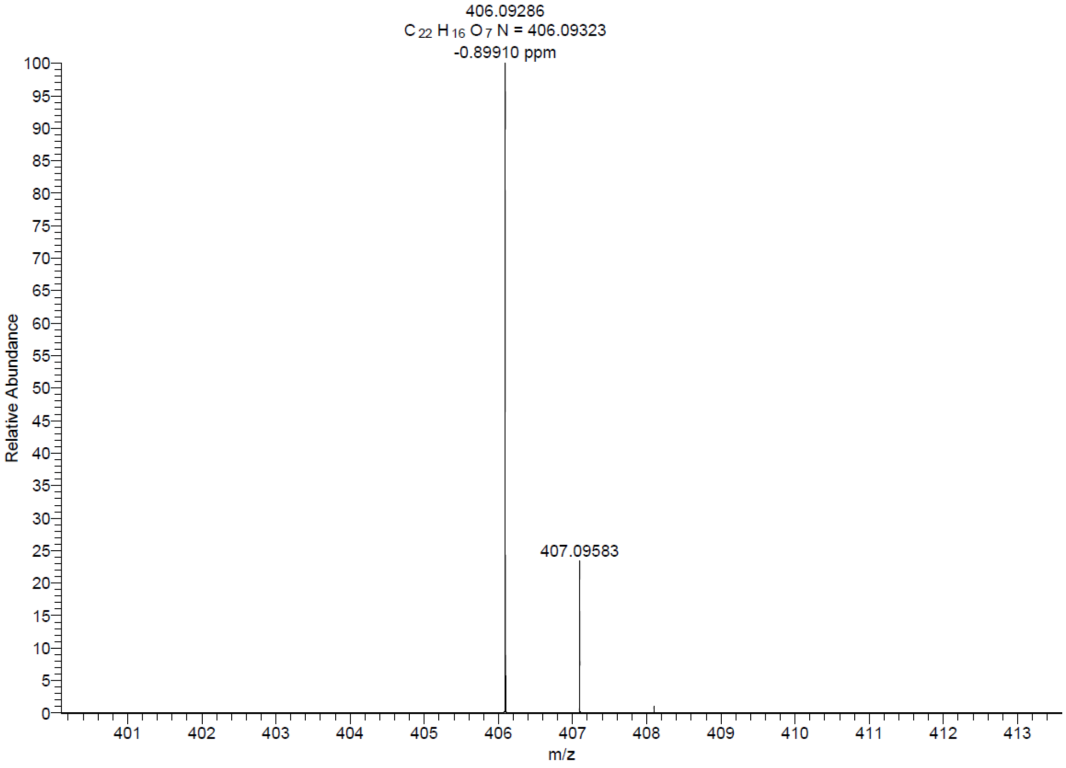


**Figure S60.** HRMS (ESI$^-$) analysis of 8-(4-methoxyanilino) luteolin (**12**). Calculated for $C_{22}H_{16}O_7N$ [M – H]$^-$ 406.09323, measured 406.09286.

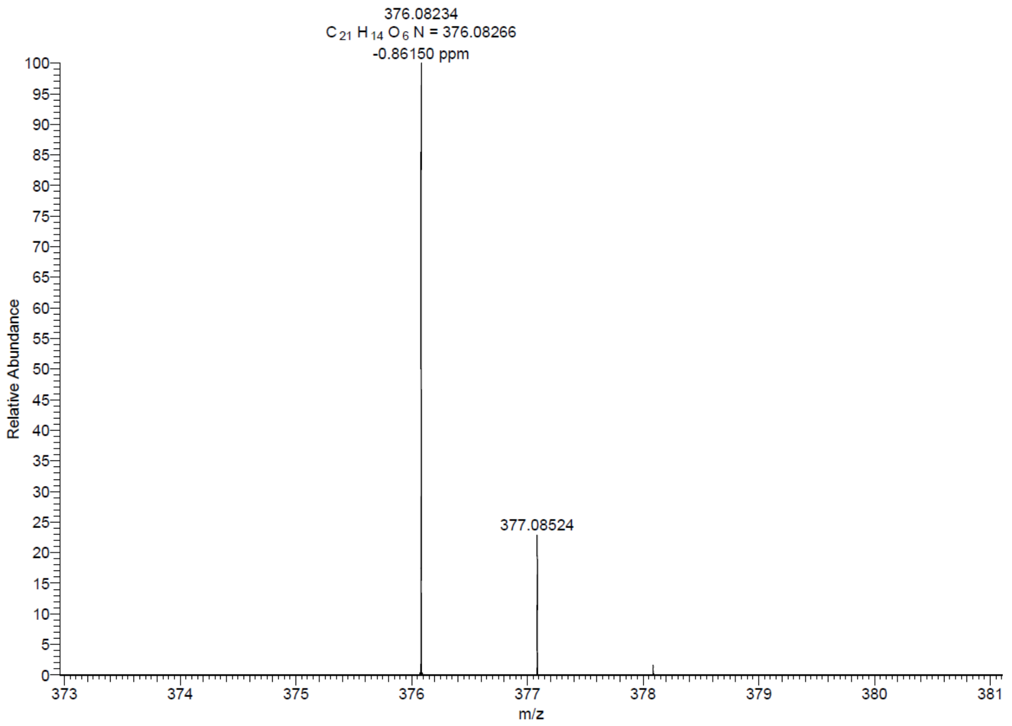


**Figure S61.** HRMS (ESI$^-$) analysis of 8-(anilino) luteolin (**13**). Calculated for $C_{21}H_{14}O_6N$ [M – H]$^-$ 376.08266, measured 376.08234.

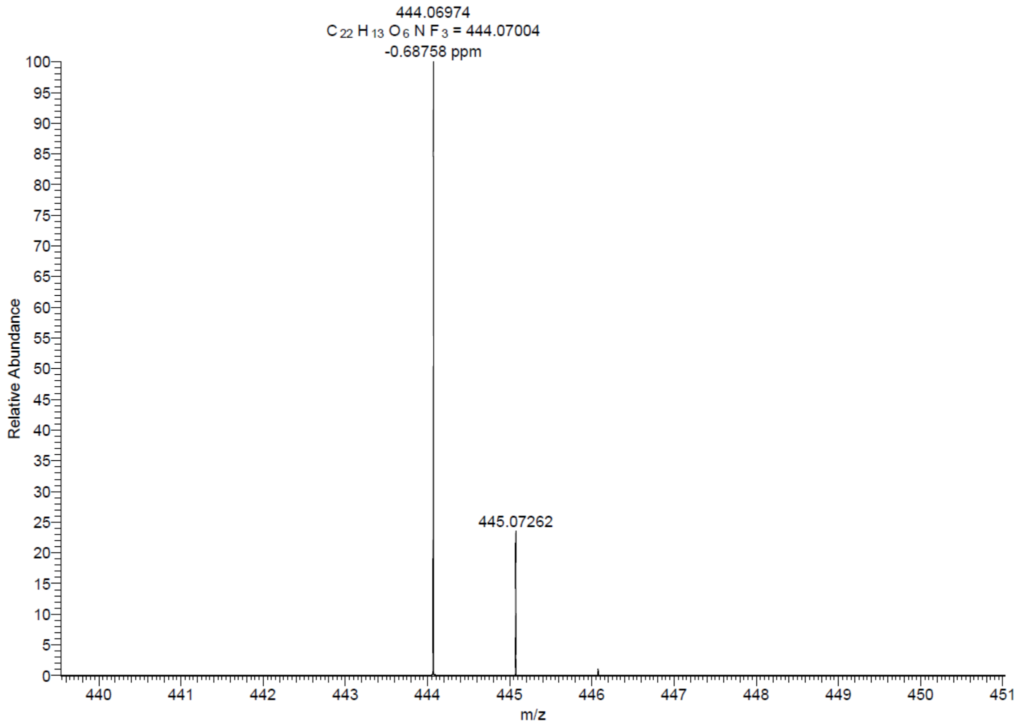


**Figure S62.** HRMS (ESI$^-$) analysis of 8-(4-(trifluoromethyl)anilino) luteolin (**14**). Calculated for $C_{22}H_{13}O_6NF_3$ [M – H]$^-$ 444.07004, measured 444.06974.

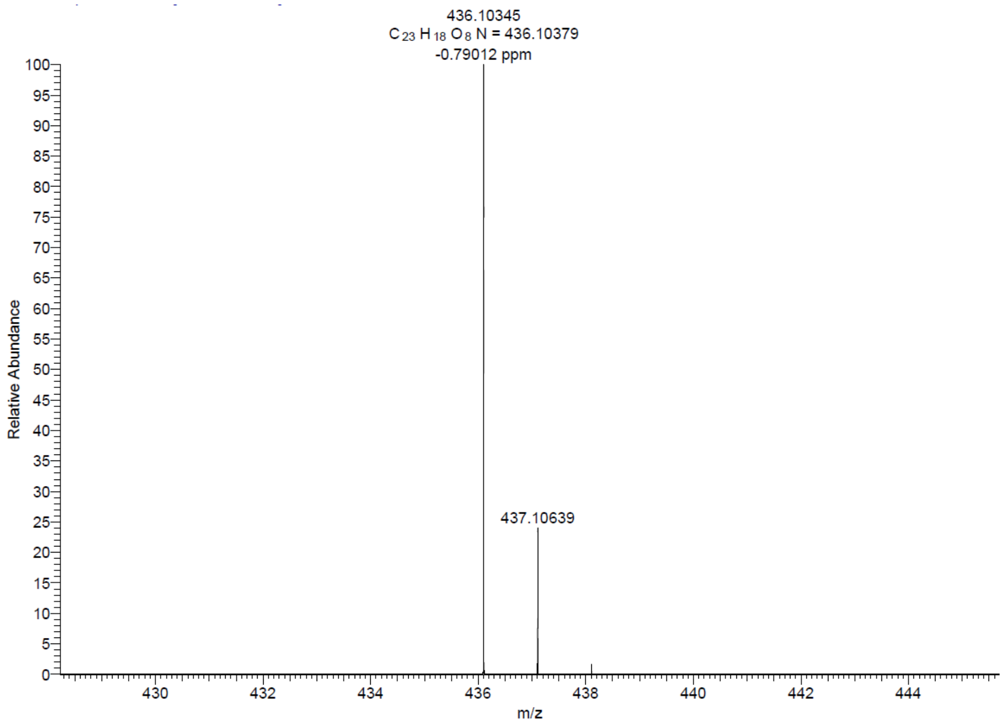


**Figure S63.** HRMS (ESI$^-$) analysis of 8-(3-fluoroanilino) luteolin (**15**). Calculated for $C_{21}H_{15}O_6NF$ [M + H]$^+$ 396.08779, measured 396.08757.

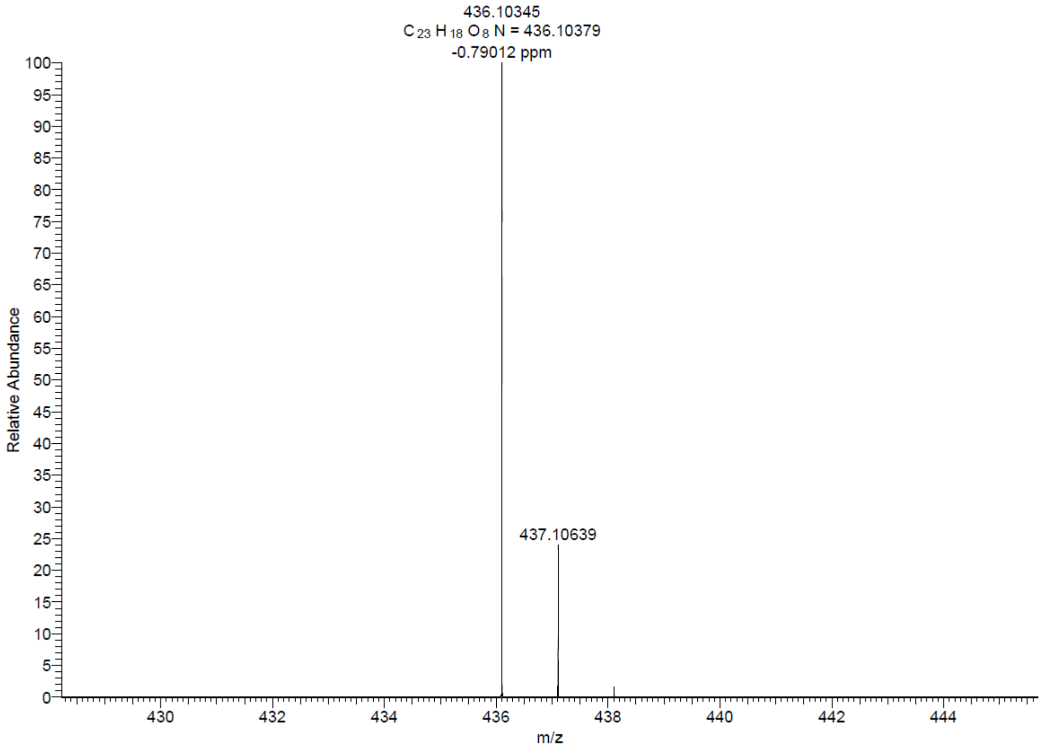


**Figure S64.** HRMS (ESI$^-$) analysis of 8-(3,5-dimethoxyanilino) luteolin (**16**). Calculated for $C_{23}H_{18}O_8N$ [M – H]$^–$ 436.10379, measured 436.10345.
